# Enhanced Proton-Coupled Electron-Transfer Reactivity by a Mononuclear Nickel(II) Hydroxide Radical Complex

**DOI:** 10.1021/acs.inorgchem.4c03370

**Published:** 2024-12-16

**Authors:** Daniel Ye, Tong Wu, Ankita Puri, David D. Hebert, Maxime A. Siegler, Michael P. Hendrich, Marcel Swart, Isaac Garcia-Bosch

**Affiliations:** † Department of Chemistry, Carnegie Mellon University, Pittsburgh, Pennsylvania 15213, United States; ‡ Johns Hopkins University, Baltimore, Maryland 21218, United States; § University of Girona, Campus Montilivi (Ciències), IQCC, 17004 Girona, Spain; ⊥ ICREA, Pg. Lluís Companys 23, 08010 Barcelona, Spain

## Abstract

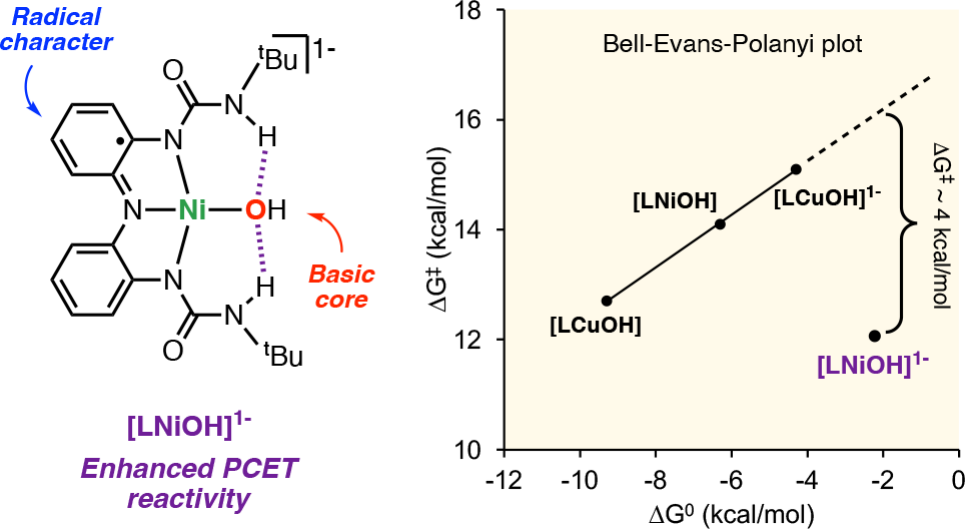

The synthesis, characterization, and reactivity of a NiOH core bearing a tridentate redox-active ligand capable of reaching three molecular oxidation states is presented in this paper. The reduced complex [LNiOH]^2–^ was characterized by single-crystal X-ray diffraction analysis, depicting a square-planar NiOH core stabilized by intramolecular H-bonding interactions. Cyclic voltammetry measurements indicated that [LNiOH]^2–^ can be reversibly oxidized to [LNiOH]^−^ and [LNiOH] at very negative reduction potentials (−1.13 and −0.39 V vs ferrocene, respectively). The oxidation of [LNiOH]^2–^ to [LNiOH]^−^ and [LNiOH] was accomplished using 1 and 2 equiv of ferrocenium, respectively. Spectroscopic and computational characterization suggest that [LNiOH]^2–^, [LNiOH]^−^, and [LNiOH] are all Ni^II^ species in which the redox-active ligand adopts different oxidation states (catecholate-like, semiquinone-like, and quinone-like, respectively). The NiOH species were found to promote H-atom abstraction from organic substrates, with [LNiOH]^−^ acting as a 1H^+^/1e^–^ oxidant and [LNiOH] as a 2H^+^/2e^–^ oxidant. Thermochemical analysis indicated that [LNiOH] was capable of abstracting H atoms from stronger O–H bonds than [LNiOH]^−^. However, the greater thermochemical tendency of [LNiOH] reactivity toward H atoms did not align with the kinetics of the PCET reaction, where [LNiOH]^−^ reacted with H-atom donors much faster than [LNiOH]. The unique stereoelectronic structure of [LNiOH]^−^ (radical character combined with a basic NiOH core) might account for its enhanced PCET reactivity.

## Abstract 2

Herein, we describe the synthesis, characterization, and reactivity of a NiOH core bearing a tridentate redox-active ligand capable of reaching three molecular oxidation states. Spectroscopic and computational characterization suggest that the NiOH complexes are all Ni^II^ species in which the redox-active ligand adopts different oxidation states (catecholate-like, semiquinone-like, and quinone-like, respectively). The unique stereoelectronic structure of the NiOH semiquinone-like species (radical character and basicity) is proposed to enhance its PCET reactivity.

## Introduction

**Figure 1:**
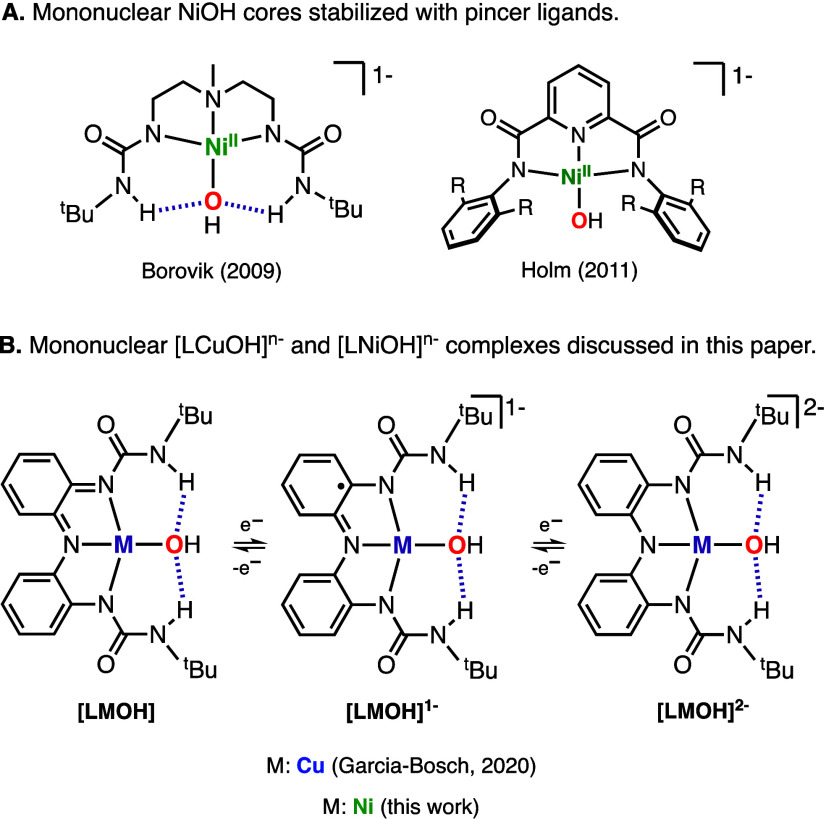
(A) Selected examples of mononuclear NiOH cores stabilized by pincer ligands. (B) Mononuclear CuOH and NiOH complexes discussed in this paper.

Mononuclear Ni hydroxide species (e.g., Ni^II^OH and Ni^III^OH) are invoked as intermediates in multiple organic transformations, including CO_2_ fixation,^1,2^ coupling reactions,^3^ and C–H bond activation.^4^ Despite their relevance, only a handful of mononuclear NiOH complexes have been synthesized and characterized (see some selected examples in Figure 1A).^5−7^ It is believed that this is due to the tendency of the hydroxide ligand to bridge between late 3d transition metals and produce species of higher nuclearity [e.g., Ni^II^_2_(OH)_2_ and Cu^II^_2_(OH)_2_ cores].^8^ The formation of multinuclear species can be avoided by using pincer ligand scaffolds with bulky substituents and/or by utilizing ligands with H-bonding donors to stabilize mononuclear M–OH cores.^9^ With this in mind, we recently utilized a tridentate redox-active ligand containing ureanyl H-bond donors to generate a mononuclear CuOH complex capable of reaching three molecular oxidation states (Figure 1B).^10,11^ Structural and spectroscopic characterization of the CuOH species (namely, [LCuOH]^2–^, [LCuOH]^−^, and [LCuOH]) suggested that these are formulated as Cu^II^OH cores with the ligand adopting different oxidation states (catecholate-like, semiquinone-like and quinone-like, respectively). In this paper, we report the synthesis, characterization, and reactivity of the analogous NiOH system.

**Figure 2:**
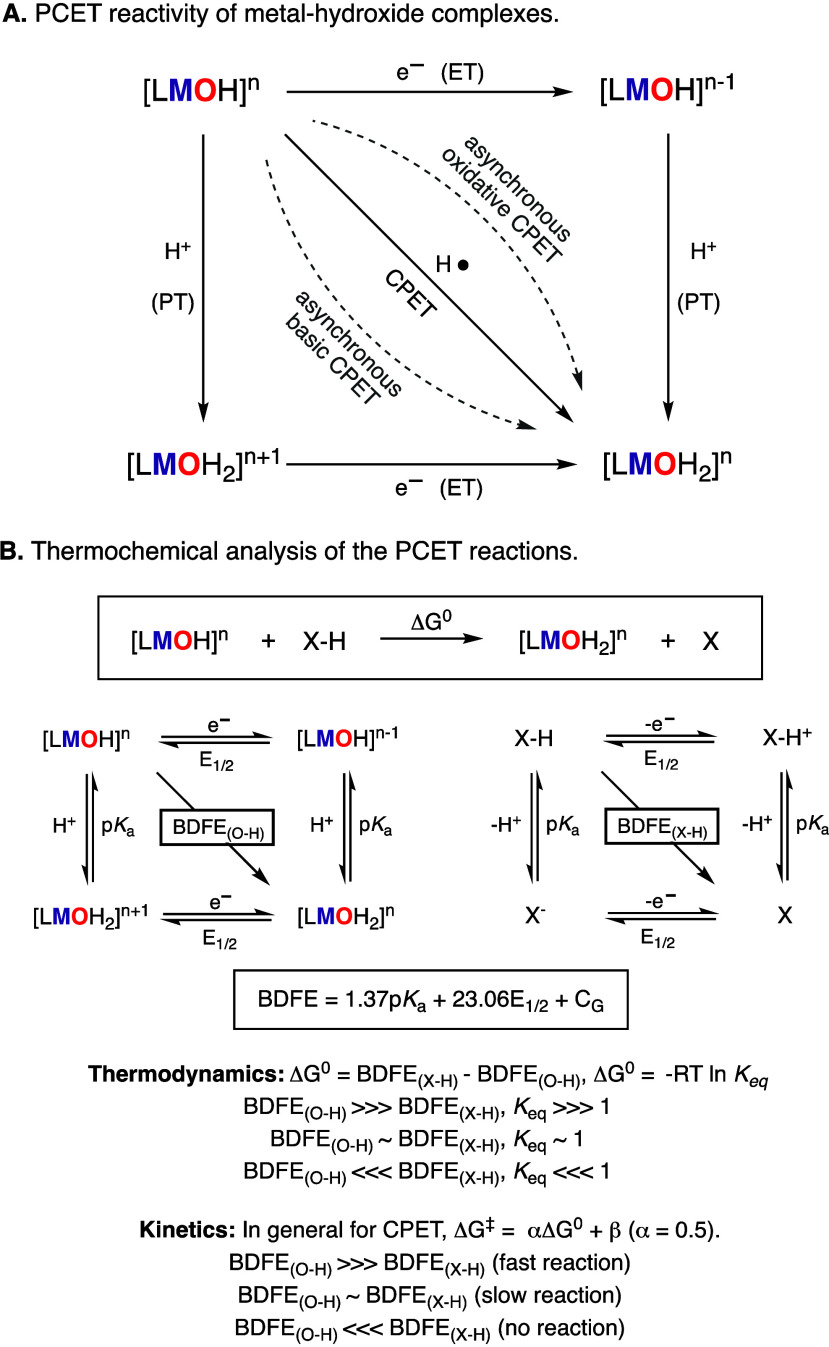
Reaction pathways (A) and thermochemical analysis (B) of the PCET reactivity of metal hydroxide complexes.

The oxidative reactivity of metal oxo and metal hydroxide species usually entails a proton-coupled electron-transfer (PCET) key step, involving the transfer of a proton (PT) and an electron (ET) from an organic substrate (C–H, N–H, and O–H bonds) to the M–O(H) species (Figure 2A).^12,13^ Classically, it is proposed that these PCET processes can occur in a concerted fashion (coupled-proton electron transfer, CPET) or stepwise fashion (proton transfer followed by electron transfer, PT–ET, or electron transfer followed by proton transfer, ET–PT). Very recently, a third category of PCET transformation has been described (asynchronous CPET) that differs from the limiting extremes mentioned above, in which the one-step CPET transformation occurs via the formation of a sole transition state with more proton-transfer character (asynchronous basic CPET) or more electron-transfer character (asynchronous oxidative CPET).^14,15^

The thermochemistry and kinetics of PCET reactions can be studied by determining the bond dissociation free energy (BDFE) of the bonds broken and formed during the reaction (Figure 2B).^12^ For example, the ability of a metal hydroxide species to accept H atoms is driven by the BDFE of the O–H bond of the resulting M-aqua complex. In general, the higher the difference between the BDFE_O–H_ of the M-aqua complex and the BDFE_X–H_ of the PCET reagent (BDFE_O–H_ ≫ BDFE_X–H_), the higher the driving force of the PCET reaction will be, which usually correlates with faster reaction rates.^13^ However, several reports have shown that the kinetics of PCET reactions depend not only on the thermochemistry of the reaction but also on the spin state of the H-atom-abstracting metal species (e.g., two-state reactivity in nonheme Fe-oxo complexes) or the mechanism by which the metal species reacts (e.g., synchronous vs asynchronous CPET in Co-oxo species or PT–ET vs CPET in Mn-oxo).^16,17^

**Figure 3:**
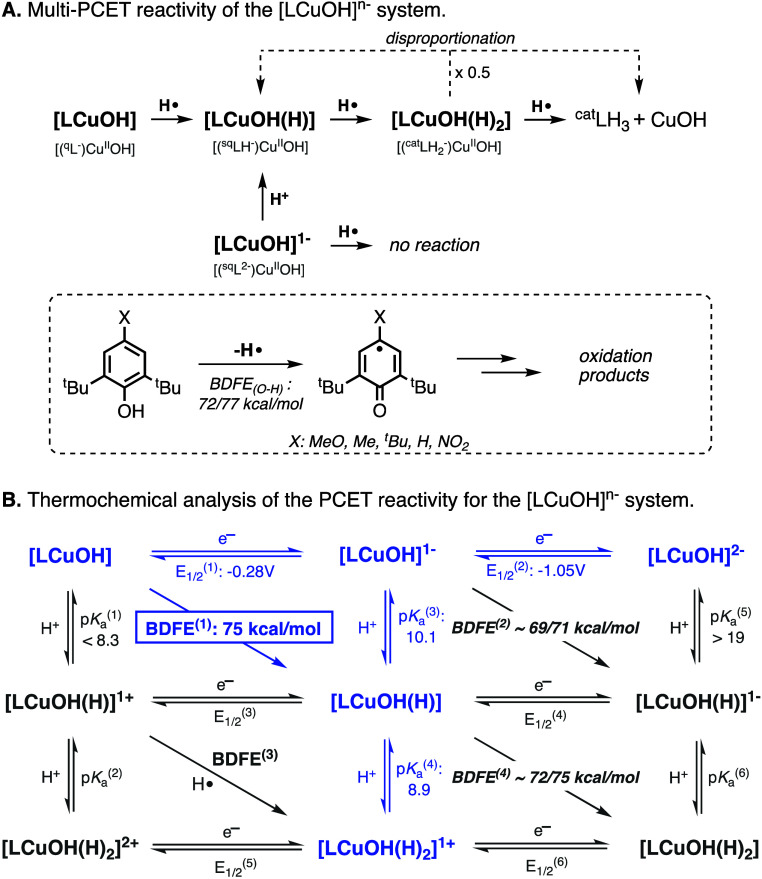
Stoichiometry (A) and thermochemical analysis (B) of the multi-PCET transformations promoted by the [LCuOH]^*n*−^ systems.

For the [LCuOH]^*n*−^ system, we have shown that the CuOH species abstracted H atoms from organic substrates containing weak O–H and N–H bonds in a multiproton multielectron fashion (Figure 3A).^10^ Our studies also indicated that the thermochemical driving force of the 1H^+^/1e^–^ reduction of the CuOH cores depended on the oxidation state of the ligand scaffold (i.e., the BDFE of the [LCuOH]/[LCuOH(H)] couple was higher than the BDFE of the [LCuOH]^−^/[LCuOH(H)]^−^ couple; Figure 3B), which impacted the reactivity of the CuOH cores, as [LCuOH] reacted with phenols while [LCuOH]^−^ did not. In this paper, we show that the PCET reactivity of the [LNiOH]^*n*−^ species also depends on the oxidation state of the complex. The PCET reactions between TEMPOH and [LNiOH]^−^ were substantially faster than the analogous reaction involving [LNiOH], despite the superior thermochemical driving force of [LNiOH] to accept H atom from organic substrates.^18,19^

## Results and Discussion

### Synthesis and Characterization of the NiOH Complex

**Figure 4:**
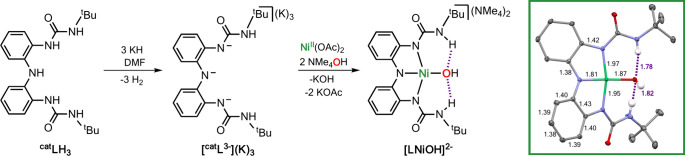
Synthesis and SC-XRD characterization of [LNiOH]^2–^. Note that the NMe_4_^+^ countercations, lattice DMF solvent molecules, and most H atoms were omitted for clarity.

The [LNiOH]^2–^ complex was obtained following a protocol similar to the one used for the synthesis of [LCuOH]^2–^ (Figure 4). In the glovebox, 3 equiv of KH was added to a DMF solution of the tridentate ligand (LH_3_), after which 1 equiv of Ni(OAc)_2_ and 2 equiv of NMe_4_OH·5H_2_O were stirred to form the complex [LNiOH](NMe_4_)_2_ [see the Supporting Information (SI) for further details].

Crystalline material suitable for single-crystal X-ray diffraction (SC-XRD) analysis was obtained by layering a DMF solution of [LNiOH]^2–^ with diethyl ether. The Ni complex exhibited a slightly distorted square-planar geometry around the nickel center, typical of d^8^ 4-coordinated Ni^II^ complexes (τ_4_ value of 0.15; for square-planar structures, a τ_4_ value of 0.0 is expected, whereas for tetrahedral structures, a τ_4_ value of 1.0 is expected^20^). Compared to the Cu complex (τ_4_ value of 0.2), the Ni system adopts a higher degree of square-planarity. The Ni–N bond distances averaged 1.91 Å, whereas the Cu–N bond averaged 1.98 Å. With respect to the metal hydroxide bond, the Ni–O bond was found to be 0.14 Å shorter than the Cu–O bond (1.88 and 2.02 Å respectively). These bond lengths, in tandem with shorter intramolecular H-bond interactions (1.80 Å for [LNiOH]^2–^ and 1.90 Å for [LCuOH]^2–^), indicated the formation of a more compact square-planar structure for the NiOH complex.

Analysis of the C_Ar_–N_α_ and C_Ar_–C_Ar_ distances (∼1.42 and ∼1.40 Å, respectively) indicated that the ligand scaffold in [LNiOH]^2–^ adopted the catecholate-like form.^10,21^^1^H NMR and EPR measurements confirmed the diamagnetic nature of [LNiOH]^2–^ (*S* = 0), which was formulated as a low-spin Ni^II^OH core bound by the fully reduced trianionic form of the ligand (see the SI).^11^

### Electrochemistry

**Figure 5:**
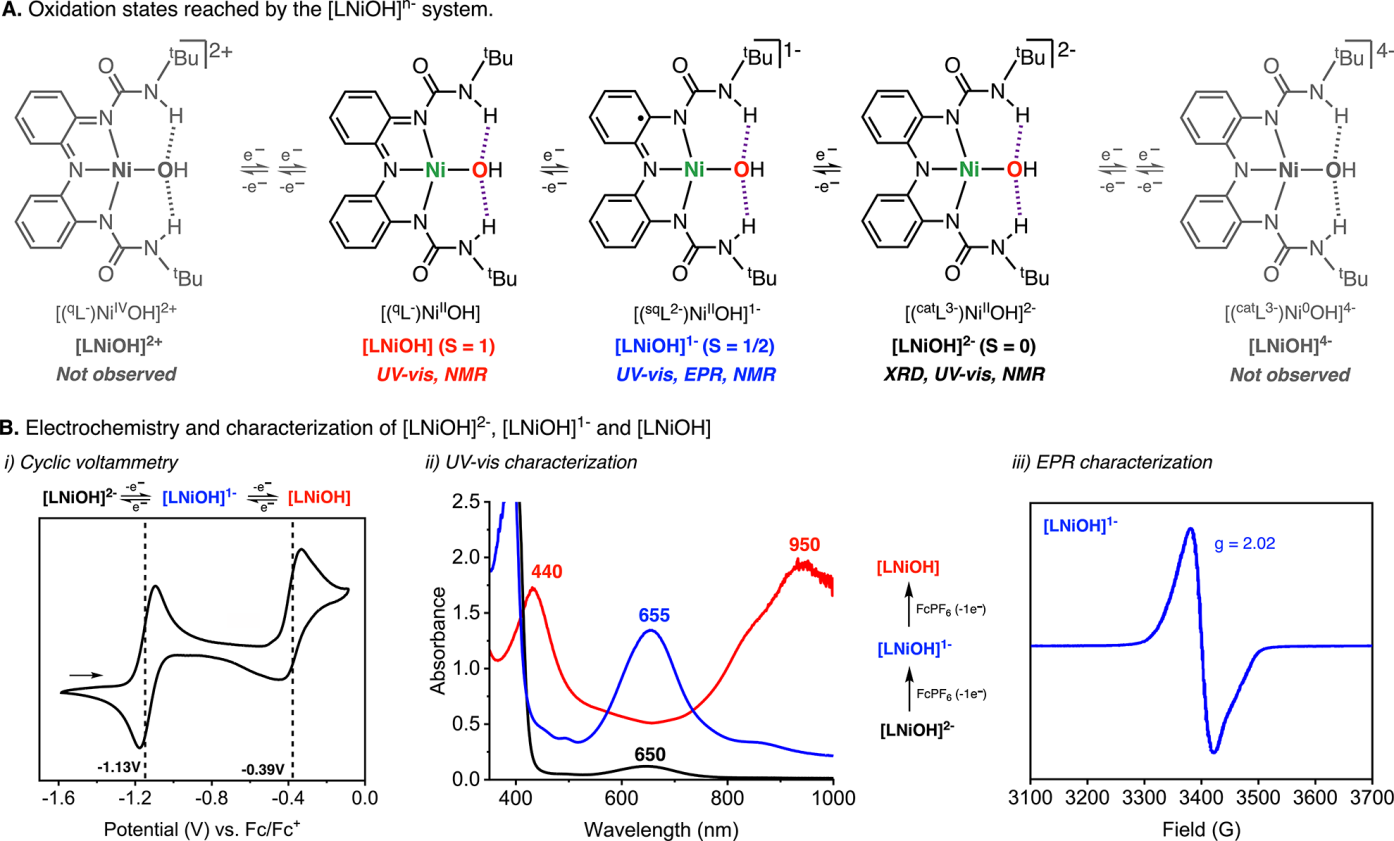
(A) Possible oxidation states that can be reached in the oxidation/reduction of the [LNiOH]^2–^ complex. (B) Electrochemical and spectroscopic characterization of [LNiOH]^2–^, [LNiOH]^−^, and [LNiOH]. Note: Only 3 of the 7 possible oxidation states ([LNiOH]^2–^, [LNiOH]^−^, and [LNiOH]) were observed by CV. See the SI for further details.

The hallmark of metal complexes bearing redox-active ligands is the propensity for these complexes to reach multiple oxidation states via ligand and/or metal reduction/oxidation events.^22−24^ In 2020, we reported three reversible, ligand-based redox events in [LCuOH]^2–^, where the ligand was reversibly oxidized from the catecholate-like (L^3–^) form to the semiquinone-like (L^•2–^), and quinone-like (L^–^) forms, and the metal center remained a Cu^II^ ion.^10^ In the current Ni system, seven oxidation states are possible, ranging from a Ni^0^ complexed with the catecholate-like form of the ligand ([LNiOH]^4–^) to a Ni^IV^ complexed by the quinone-like form of the ligand ([LNiOH]^2+^; Figure 5). However, cyclic voltammetry experiments showed three observable states, where [LNiOH]^2–^ can be reversibly oxidized to [LNiOH]^−^ and [LNiOH] at −1.13 and −0.39 V, respectively (V vs ferrocene, Fc^0/+^). Both of these redox events are comparatively more reductive than the Cu system, which undergoes redox events at −1.05 and −0.28 V, correspondingly.

The oxidation of [LNiOH]^2–^ to the “high-valent” oxidation states [LNiOH]^−^ and [LNiOH] can also be accomplished by stoichiometric additions of an oxidant such as ferrocenium hexafluorophosphate (FcPF_6_; Figure 5B). The addition of 1 equiv of FcPF_6_ to [LNiOH]^2–^ (650 nm, ε = 600 M^–1^ cm^–1^) generated a new species that absorbed at 655 nm (ε = 5600 M^–1^ cm^–1^), which was assigned as [LNiOH]^−^. This species was stable at room temperature (*t*_1/2_ > 24 h). However, attempts to crystallize this complex were futile at both room and low temperatures. The subsequent addition of 1 equiv of FcPF_6_ generated a species with absorbances at 440 nm (ε = 7200 M^–1^ cm^–1^) and 950 nm (ε = 8000 M^–1^ cm^–1^), which was assigned as [LNiOH]. This species was metastable at room temperature (*t*_1/2_ ∼ 40 min). After the formation of [LNiOH] from FcPF_6_, stoichiometric additions of the 1e^–^ reductant cobaltocene reduced the complex to regenerate [LNiOH]^−^ and [LNiOH]^2–^, respectively (see the SI).

Due to the ambiguous electronic nature of the metal complexes bound by redox-active ligands (noninnocent ligands), [LNiOH]^−^ can be formulated as a Ni^II^ ion bound by the semiquinone-like form of the ligand or a Ni^III^ bound by the catecholate-like form, among other possibilities. Evidence for the formulation of [LNiOH]^−^ as a semiquinone-like Ni^II^OH species was obtained by EPR, in which a spectrum characteristic of a species containing a ligand-centered radical (i.e., semiquinone-like ligand) was observed (Figure 5B). These results contrast with the EPR spectra of known Ni^III^ complexes.^25^ The magnetic susceptibility of [LNiOH]^−^ was measured at room temperature using the Evans method (see the SI). The μ_eff_ obtained (1.77 μ_B_) was consistent with the formation of a low-spin *S* = ^1^/_2_ complex, in agreement with the EPR measurements.

Complex [LNiOH] was also characterized by UV–vis, EPR, and NMR spectroscopy. The absorption spectrum of [LNiOH] was similar to the one observed for [LCuOH], which is characteristic of metal complexes bound by tridentate quinone-like ligands.^21^ The formulation of [LNiOH] as a paramagnetic Ni^II^ ion bound by a quinone-like ligand was also supported by EPR (no signal in standard perpendicular mode, *S* = 1) and NMR (paramagnetic spectrum; see the SI). Magnetic susceptibility measurements using the Evans method were also consistent with the formulation of [LNiOH] as a high-spin triplet paramagnetic species (2.81 μ_B_).

### DFT Calculations

**Figure 6:**
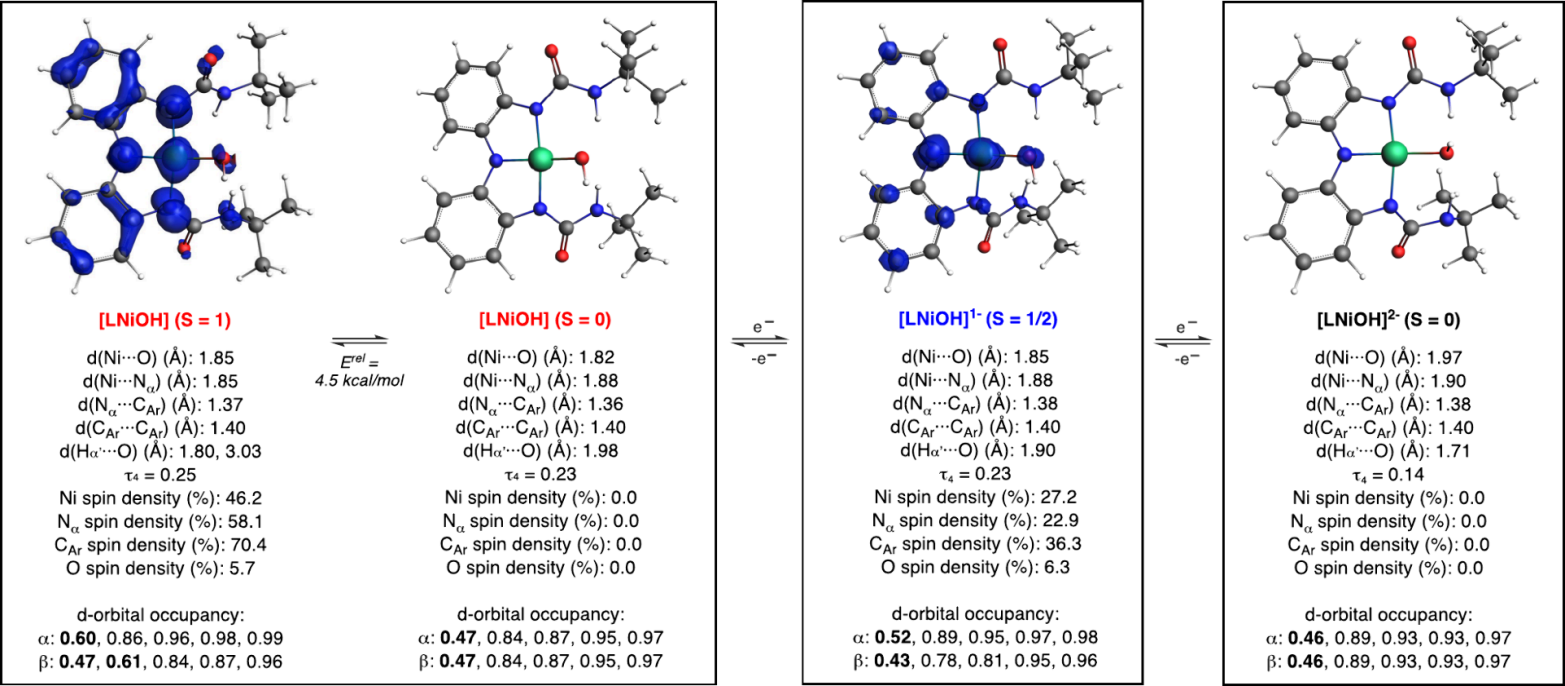
DFT calculations on the electronic structure of the [LNiOH]^*n*−^ systems. Note: see the SI for details on the DFT computations, including the structure, spin density plots, and geometric parameters of the other spin isomers of the complexes (e.g., the isomer in the triplet spin state of [LNiOH]^2–^).

Computations provided additional evidence on the geometry and electronic structure of the [LNiOH]^*n*−^ complexes (Figure 6). For each species, we optimized the geometry of the complex in two possible spin states and computed the difference in energy between the two isomers. For example, [LNiOH]^2–^ could adopt two different spin states, a diamagnetic isomer (*S* = 0) or a paramagnetic isomer (*S* = 1). Computations on [LNiOH]^2–^ suggested that the isomer in the singlet spin state (*S* = 0) was ∼8 kcal/mol lower than the isomer in the triplet state (*S* = 1). Hence, DFT calculations agreed with the experimental evidence (NMR, magnetic moment measurements) that formulated [LNiOH]^2–^ as a diamagnetic species (see the sections above). Computation of the d-orbital occupancy in [LNiOH]^2–^ indicated that the Ni ion was a d^8^ metal (8 of out of 10 of the electrons in the d orbitals had a higher occupancy than 0.7^26^), implying that the ligand scaffold was in the catecholate-like form.

Computations on [LNiOH]^−^ found that the isomer in the doublet spin state (*S* = ^1^/_2_) was ∼13 kcal/mol lower than the isomer in the quadruplet spin state (*S* = ^3^/_2_). Like in [LNiOH]^2–^, the d-orbital occupancy calculations on [LNiOH]^−^ (*S* = ^1^/_2_) indicated that the Ni ion is a d^8^ metal, suggesting that the ligand scaffold was in the semiquinone-like form and in agreement with the EPR measurements. This hypothesis was further confirmed with the spin density plot (visualization of the “electron density of unpaired electrons”), in which substantial spin in the N and C atoms of the redox-active ligand was observed (Figure 6).

For [LNiOH], our DFT calculations suggested that the isomer in the singlet spin state (*S* = 0) was ∼4.5 kcal/mol lower in energy than the isomer in the triplet spin state (*S* = 1). The small energy gap between the two isomers is somewhat in agreement with paramagnetism recorded, suggesting that the triplet spin state is significantly populated at room temperature. Based on the d-orbital occupancy, the singlet spin state isomer was formulated as a Ni^II^ complex (d^8^) bound by the quinone-like form of the ligand. Computations on the isomer in the triplet spin state were consistent with a Ni^III^OH core bound by a semiquinone-like ligand, in which the unpaired metal-based electron is ferromagnetically coupled with the ligand-centered radical. As previously stated, UV–vis and NMR measurements suggest that [LNiOH] is formulated as a quinone-like Ni^II^ paramagnetic complex. The origin for the discrepancy between the experiments and the computations is unclear, but it could be due to a change in the geometry of the Ni ion (e.g., formation of tetrahedral Ni species, which could favor the high-spin configuration^27^), solvent coordination (inducing the formation of a pseudo-octahedral Ni^II^ paramagnetic complex^28^), or other changes (e.g., ureanyl reorientation to bind with either NH or carbonyl or keto–enol urea isomerization^29^). Calculations on [LNiOH] coordinated by two molecules of DMF found that both isomers (singlet and triplet spin state) were formulated as Ni^II^OH quinone-like species, in agreement with the spectroscopic data (see the SI).

Our computations indicate that the oxidation of the ligand from the catecholate-like form in [LNiOH]^2–^ to the semiquinone-like form in [LNiOH]^−^ and quinone-like form in [LNiOH] entailed shortening of the C···N_α_ bond (from 1.38 to 1.36 Å). The sequential oxidation of [LNiOH]^2–^ to [LNiOH]^−^ and [LNiOH] also led to shortening of the Ni–O bond (from 1.97 to 1.82 Å), elongation of the intramolecular H-bonding interactions (H_α′_···O, from 1.71 to 1.98 Å), and a slight increase of the τ_4_ value (from 0.14 to 0.23).

### PCET Reactivity of the [LNiOH]^*n*−^ Complexes: Scope and Stoichiometry

**Figure 7:**
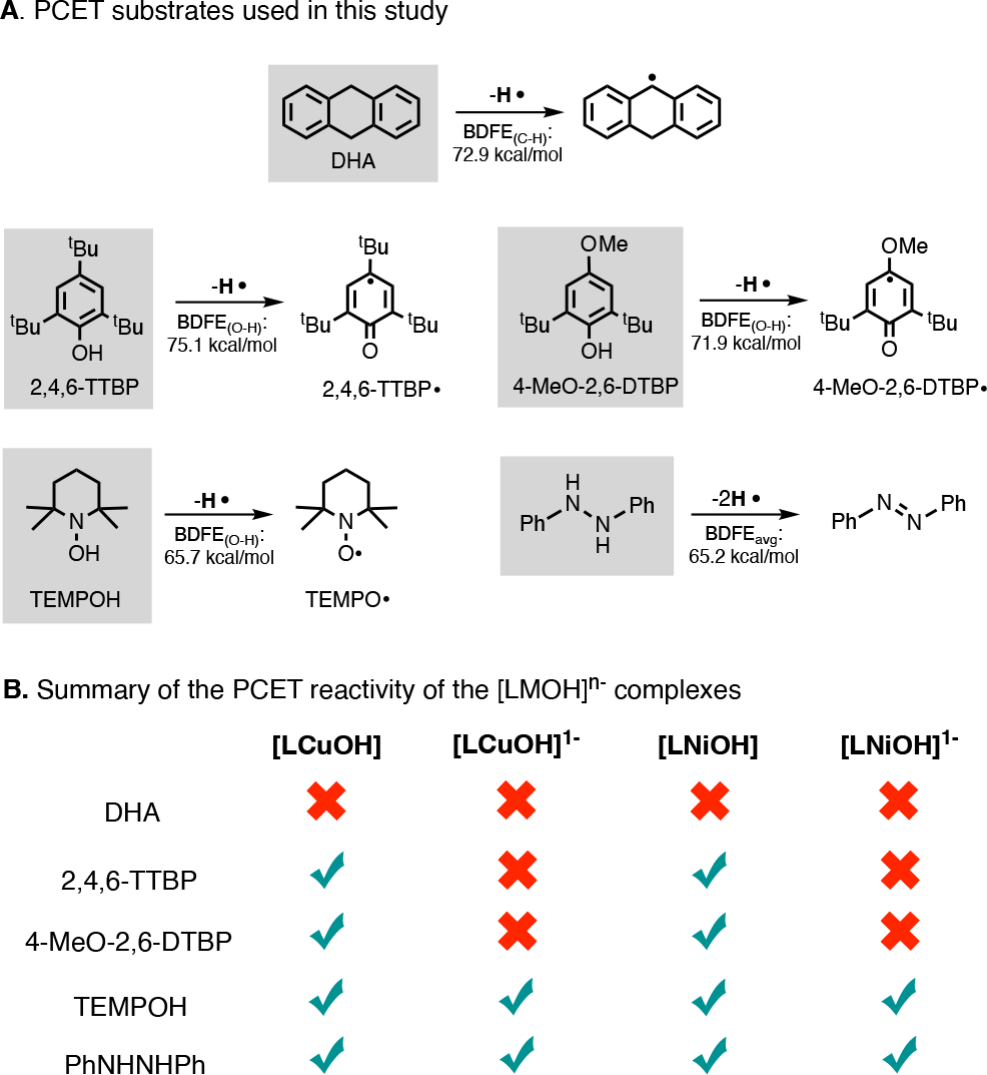
Reactivity of [LMOH]^*n*−^, including the substrates utilized (A) and variations on the reactivity depending on the oxidation state of the complex (B).

The H-atom-abstraction reactivity of [LNiOH]^−^ and [LNiOH] with PCET reagents was studied in detail (Figure 7). Similar to the CuOH system, none of the NiOH complexes were able to react with organic substrates with weak C–H bonds (e.g., dihydroanthracene, DHA). The NiOH systems can, however, perform H-atom abstractions on substrates containing weak O–H and N–H bonds. [LNiOH]^−^ reacted with 1-hydroxy-2,2,6,6-tetramethylpiperidine (TEMPOH; BDFE_O–H_ = 65.7 kcal/mol) and with diphenylhydrazine (PhNHNHPh; BDFE_avg_ = 65.2 kcal/mol) but not with 4-substituted 2,6-di-*tert*-butylphenols such as 4-methoxy-2,6-*tert*-butylphenol (4-MeO-2,6-DTBP; BDFE_O–H_ = 71.9 kcal/mol) and 2,4,6-tri-*tert*-butylphenol (2,4,6-TTBP; BDFE_O–H_ = 75.1 kcal/mol). [LNiOH], on the other hand, reacted with TEMPOH, PhNHNHPh, and the above-mentioned phenols (note that the BDFE values of the substrates and complexes discussed in this and the following section have been determined experimentally with an uncertainty of 1 kcal/mol unless stated^12,30^).

**Figure 8:**
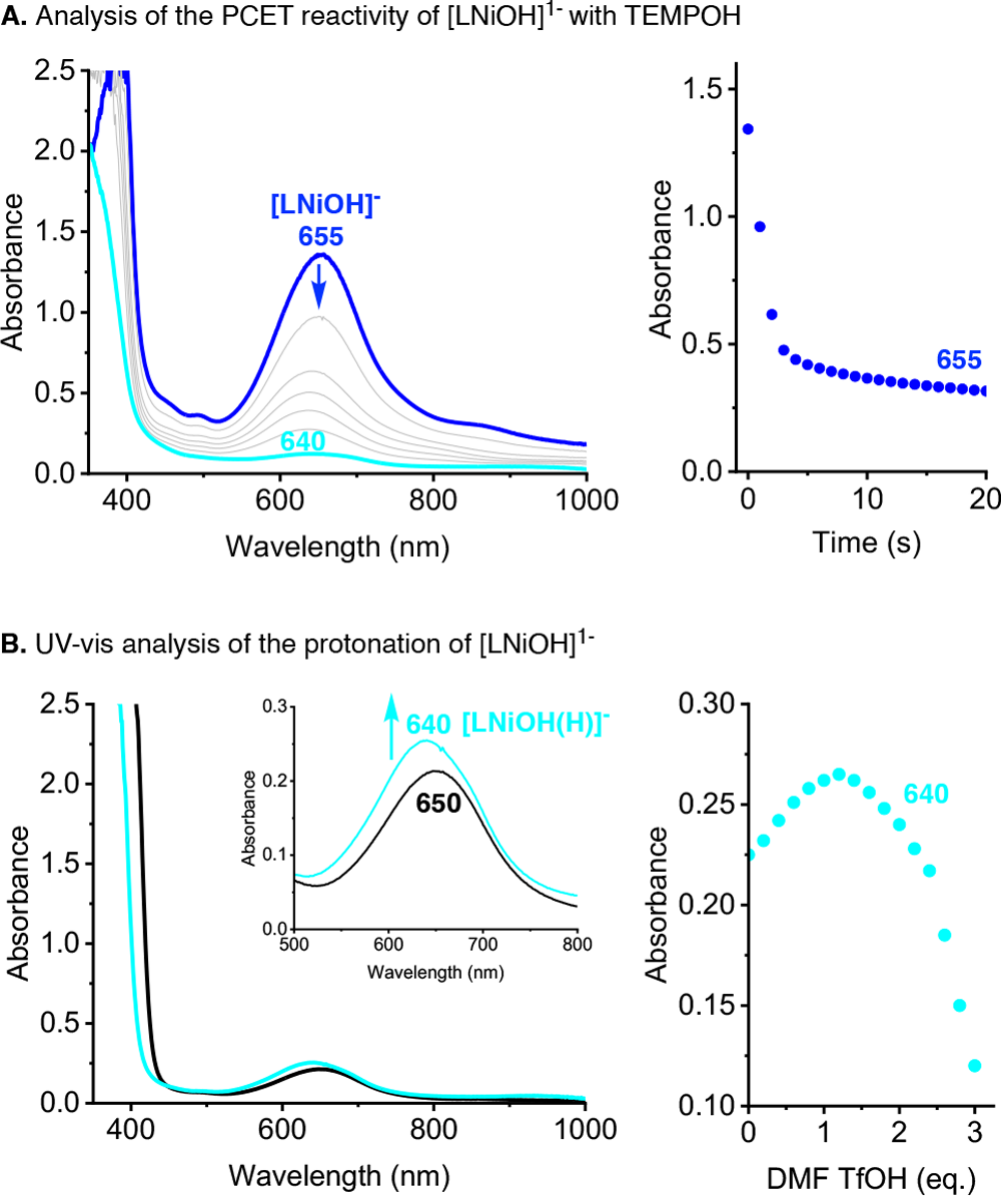
UV–vis analysis of the reaction between [LNiOH]^−^ and TEMPOH (A) and protonation of [LNiOH]^2–^ (B).

The stoichiometry of the reactions between [LNiOH]^−^ and [LNiOH] with PCET reagents was determined by UV–vis, NMR, and EPR (note: see the overall proposed stoichiometry in Figure 10). The UV–vis spectra of the reaction of [LNiOH]^−^ (0.25 mM) with 20 equiv of TEMPOH at room temperature showed fast decay of the features associated with [LNiOH]^−^ (λ_max_ = 655 nm; Abs ∼1.5) to produce a Ni complex with features that resembled [LNiOH]^2–^ (λ_max_ = 640 nm; Abs ∼0.2), suggesting that [LNiOH]^−^ underwent a 1H^+^/1e^–^ reduction (Figure 8A). EPR analysis of the reaction between [LNiOH]^−^ and TEMPOH confirmed the formation of 1 equiv of TEMPO radical (yield ∼80%; see the SI). The addition of 1 equiv of a strong acid (DMF·CF_3_SO_3_H) to [LNiOH]^2–^ produced the same UV–vis spectrum (Figure 8B), suggesting that the final product of the protonation/reduction of [LNiOH]^−^ (namely, [LNiOH(H)]^−^) was a Ni^II^ complex with a catecholate-like form of the ligand (Figure 8B; note that one of the possible structures of [LNiOH(H)]^−^ is depicted in Figure 10; see also the SI). Additional DMF·CF_3_SO_3_H led to a decrease of the UV–vis features of [LNiOH(H)]^−^, suggesting that the catecholate-like complex can be further protonated. NMR analysis of the protonation of [LNiOH]^−^ indicated that the addition of 1 equiv of acid produced the diamagnetic species [LNiOH(H)]^−^, which reacts with additional acid to form 1 equiv of ligand ^cat^LH_3_.

The reaction of [LNiOH]^−^ with PhNHNHPh was followed by UV–vis and NMR (see the SI). Like in the reaction with TEMPOH, the addition of PhNHNHPh to [LNiOH]^−^ caused fast decay of its UV–vis features to produce [LNiOH(H)]^−^. The ^1^H NMR spectrum of the reaction between [LNiOH]^−^ and PhNHNHPh confirmed that [LNiOH]^−^ acted as a 1H^+^/1e^–^ acceptor, producing 0.5 equiv of PhN=NPh and the diamagnetic catecholate-like complex [LNiOH(H)]^−^.

**Figure 9:**
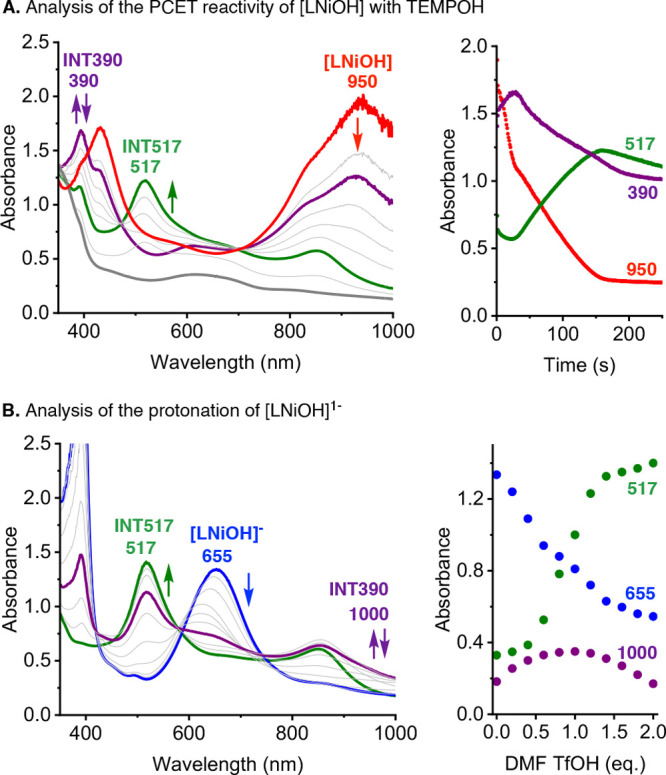
UV–vis analysis of the reaction between [LNiOH] and TEMPOH (A) and protonation of [LNiOH]^−^ (B). See the SI for details.

The reaction of [LNiOH] with TEMPOH was followed by UV–vis and EPR (see Figure 9 and the SI). The addition of excess amounts of TEMPOH to [LNiOH] caused spectral changes characteristic of multistep reactivity. The UV–vis features of [LNiOH] (λ_max_ = 950 nm; Abs ∼2.0) decayed to produce two reaction intermediates (INT390, λ_max_ = 390 nm; INT517, λ_max_ = 517 nm), which sequentially formed and decayed to generate a species with weak absorption (λ_max_ = 640 nm). EPR analysis of the reaction between [LNiOH] with TEMPOH confirmed the formation of 2 equiv of TEMPO radical, suggesting that [LNiOH] acted as a 2H^+^/2e^–^ acceptor. The reaction between [LNiOH] with PhNHNHPh was followed by UV–vis and NMR (see the SI). The UV–vis spectra recorded were similar to that of the stepwise reaction between [LNiOH] with TEMPOH (i.e., formation and decay of intermediate species with λ_max_ = 517 nm; see the SI). NMR analysis indicated that [LNiOH] reacted as the 2H^+^/2e^–^ acceptor, producing 1 equiv of PhN=NPh and 1 equiv of ligand (^cat^LH_3_).

Further evidence on the identity of the intermediates formed in the reductive protonation of [LNiOH] was obtained via protonation of [LNiOH]^−^ (Figure 9B). The reaction was followed by UV–vis, NMR, and EPR (see the SI for details). The addition of stoichiometric amounts of DMF·CF_3_SO_3_H (from 0.2 to 0.4 equiv) led to a slight shift in the UV–vis band associated with [LNiOH] and the formation of a new band at ∼1000 nm, consistent with the formation of INT390. Further addition of acid (from 0.4 to 1 equiv) led to an increase on the UV–vis features associated with the formation of INT517. Full decay of INT390 and full formation of INT517 species were accomplished by adding an additional 1 equiv of acid (total of 2 equiv of DMF·CF_3_SO_3_H; Figure 9B). After protonation of [LNiOH]^−^ with 2 equiv of acid, the addition of excess amounts of strong base did not lead to regeneration of [LNiOH]^−^, which suggests that this process is irreversible. In a separate UV–vis experiment, we reacted [LNiOH]^−^ with 2 equiv of acid (added all at once), and we observed the initial formation of INT390, which evolved to form INT517 (see the SI).

NMR analysis of the protonation of [LNiOH]^−^ with 1 equiv of acid showed the formation of ∼0.5 equiv of free ligand and a diamagnetic Ni species (see the SI). NMR analysis of the protonation of [LNiOH]^−^ with 2 equiv of acid showed a similar spectrum, with slightly more ligand formed. EPR analysis of the reaction after addition of 1 equiv of acid led to a substantial decrease in [LNiOH]^−^(see the SI). The addition of 2 equiv of acid led to quenching of all of the EPR signals, suggesting the formation of an EPR-silent species.

**Figure 10:**
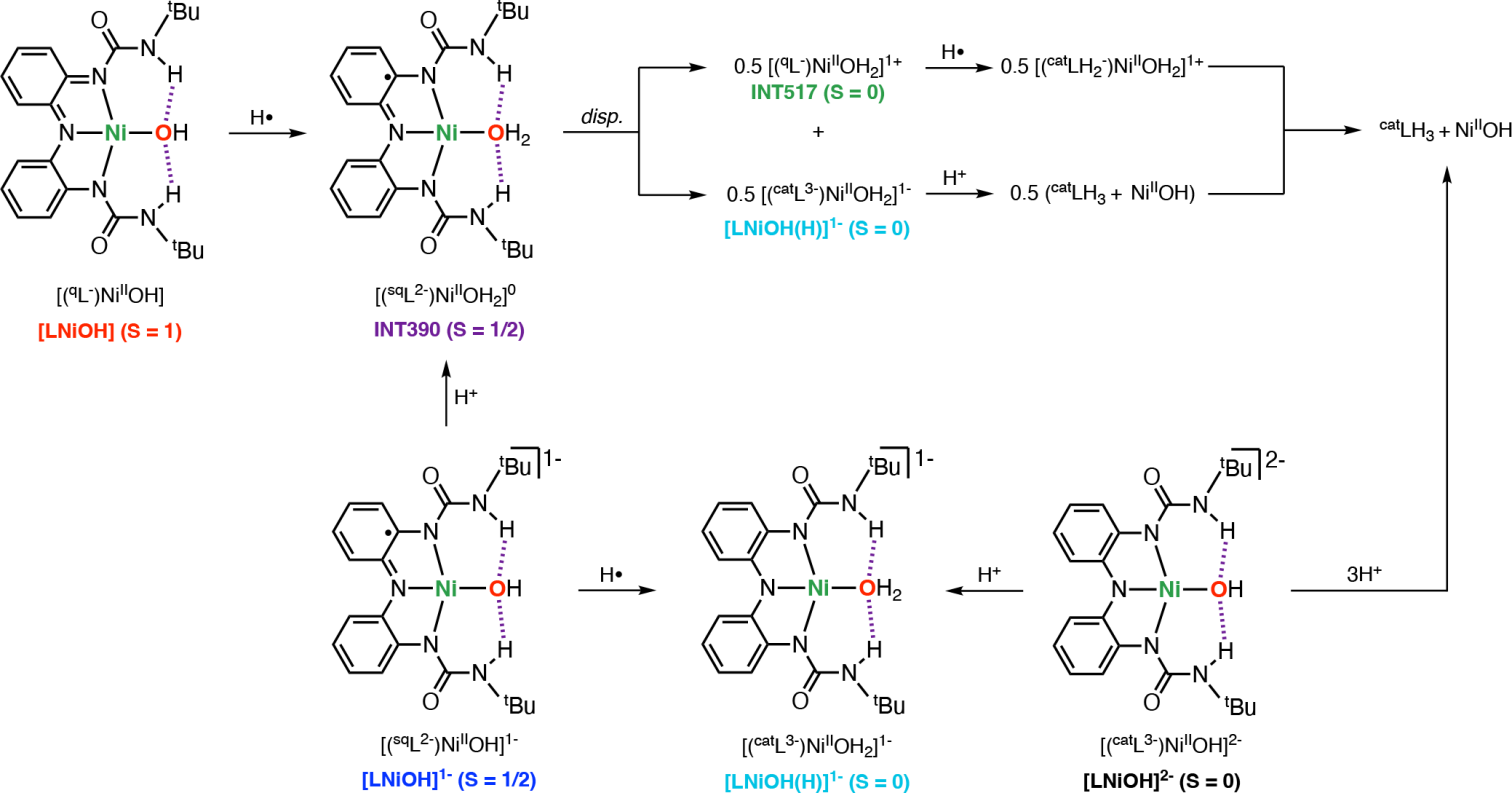
Summary of the stoichiometry and species formed in the reductive protonation of [LNiOH] and [LNiOH]^−^. Note that the structures of INT390, INT517, and [LNiOH(H)]^−^ are ambiguous. One proposed structure is shown in this figure. See the SI for additional structures.

On the basis of all of this evidence, we propose that the 1H^+^/1e^–^ reductive protonation of [LNiOH] and the protonation of [LNiOH]^−^ produce a metastable mononuclear Ni-semiquinone complex (INT390) that undergoes an irreversible disproportionation reaction to generate 0.5 equiv of a catecholate-like complex ([(^cat^L^3–^)NiOH_2_]^−^) and 0.5 equiv of a quinone-like complex (INT517; Figure 10). Our data suggest that once formed, the catecholate-like Ni^II^ species is irreversibly protonated to generate ligand (^cat^LH_3_) and free Ni^II^ in solution, which explains the fact that 2 equiv of acid is required to fully protonate [LNiOH]^−^. In the presence of substrate (e.g., TEMPOH), the diamagnetic quinone-like complex (INT517) undergoes reductive protonation, which also leads to the formation of ^cat^LH_3_ and free Ni^II^ in solution. To corroborate this hypothesis, we carried out the protonation of [LNiOH]^−^, followed by the addition of PCET reagents (see the experimental details and spectra in the SI). EPR analysis of the reaction between [LNiOH]^−^, 2 equiv of acid, and TEMPOH confirmed the formation of 1 equiv of TEMPO radical (∼80%). NMR analysis of the reaction between [LNiOH]^−^, 2 equiv of acid, and PhNHNHPh led to the formation of 0.5 equiv of PhN=NPh and 1 equiv of ligand (^cat^LH_3_). Evidence on the formation of free Ni^II^ in solution after protonation of [LNiOH]^−^ and protonation of [LNiOH]^2–^ was obtained by adding bipyridine ligand, which produced the paramagnetic [Ni^II^(bpy)_3_]^2+^ complex that could be observed by NMR (see the SI).

### PCET Reactivity of the [LNiOH]^*n*−^ Complexes: Thermochemistry

**Figure 11:**
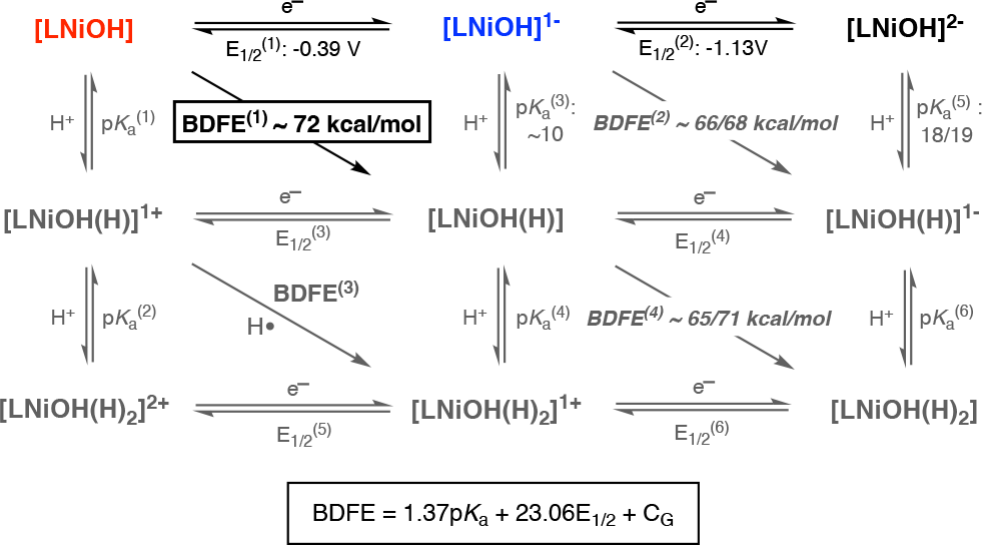
Summary of the BDFE analysis of the species involved in the PCET reactivity of the [LNiOH]^*n*−^ complexes.

In the reaction between [LNiOH] and 2,4,6-TTBP, we observed that the addition of excess amounts of phenolic substrate caused only partial decay of the UV–vis features of [LNiOH]. This behavior is usually observed when species involved in the PCET reaction have similar BDFEs, which leads to a PCET equilibrium. Given the BDFE_O–H_ of 2,4,6-TTBP (75.1 kcal/mol in DMF) and the fact that excess 2,4,6-TTBP (e.g., 100 equiv) is required for [LNiOH] to partially react, we can calculate that the BDFE for the 1H^+^/1e^–^ reductive protonation of [LNiOH] is ∼71 kcal/mol (see the SI for details). As we discussed in the Introduction, the thermochemical driving force (BDFE) for proton–electron acceptors (e.g., metal hydroxide) can also be determined using the Bordwell equation and the corresponding *E*_1/2_ and p*K*_a_ values.^12^ A partial protonation of [LNiOH]^−^ can be accomplished by 4-NO_2_–PhOH (p*K*_a_ = 12.6 in DMF^31^). Thus, the p*K*_a_ of the protonation of [LNiOH]^−^ is calculated to be ∼10 (see the SI for details). By the Bordwell equation, this results in a BDFE of ∼72 kcal/mol, in agreement with the reactivity between [LNiOH] and 2,4,6-TTBP (Figure 11).

Because [LNiOH]^−^ reacts with TEMPOH (BDFE_O–H_ = 65.7 kcal/mol) but not with 4-MeO-2,6-DTBP (BDFE_O–H_ = 71.9 kcal/mol), we can estimate the BDFE for the 1H^+^/1e^–^ reductive protonation [LNiOH]^−^ to be between 65 and 72 kcal/mol. [LNiOH]^2–^ was found to be protonated by 4-NO_2_-2,6-DTBP (BDFE = 79 kcal/mol; p*K*_a_ = 8.3 in DMF^31^) and partially protonated with excess 2,6-DTBP (BDFE = 76.7 kcal/mol; p*K*_a_ = 18.2 in DMF^31^) and 4-MeO-2,6-DTBP (BDFE = 71.9 kcal/mol; p*K*_a_ = 19.0 in DMF^31^), suggesting that the p*K*_a_ value for protonating [LNiOH]^2–^ is ∼18–19. Considering this estimation and the *E*_1/2_ of [LNiOH]^−^/[LNiOH]^2–^ couple (−1.13 V vs Fc^0/+^), we can narrow down the BDFE for the reductive protonation of [LNiOH]^−^ to be between 66 and 68 kcal/mol (Figure 11).

Our data suggest that the BDFE for the reductive protonation of [LNiOH] was slightly lower than that for [LCuOH] (72 vs 75 kcal/mol). In fact, [LCuOH] was fully reduced by 2,4,6-TTBP, while [LNiOH] reacted partially with this PCET substrate. This could be attributed to the lower redox potential of the [LNiOH]/[LNiOH]^−^ couple (−0.39 V vs Fc^0/+^) when compared to the analogous Cu couple (−0.28 V vs Fc^0/+^) because the p*K*_a_ values for the protonation of [LNiOH]^−^ and [LCuOH]^−^ are comparable (∼10). Similarly, the BDFE for the reductive protonation of [LNiOH]^−^ was also slightly lower than that for the Cu counterpart (69–71 vs 66–68 kcal/mol). This could also be explained by the lower *E*_1/2_ of the [LNiOH]^−^/[LNiOH]^2–^ couple (−1.13 V) when compared to Cu (−1.05 V), where both [LMOH]^2–^ systems have similar p*K*a values (∼18/19).

### PCET Reactivity of the [LNiOH]^*n*−^ Complexes: Kinetics

**Figure 12:**
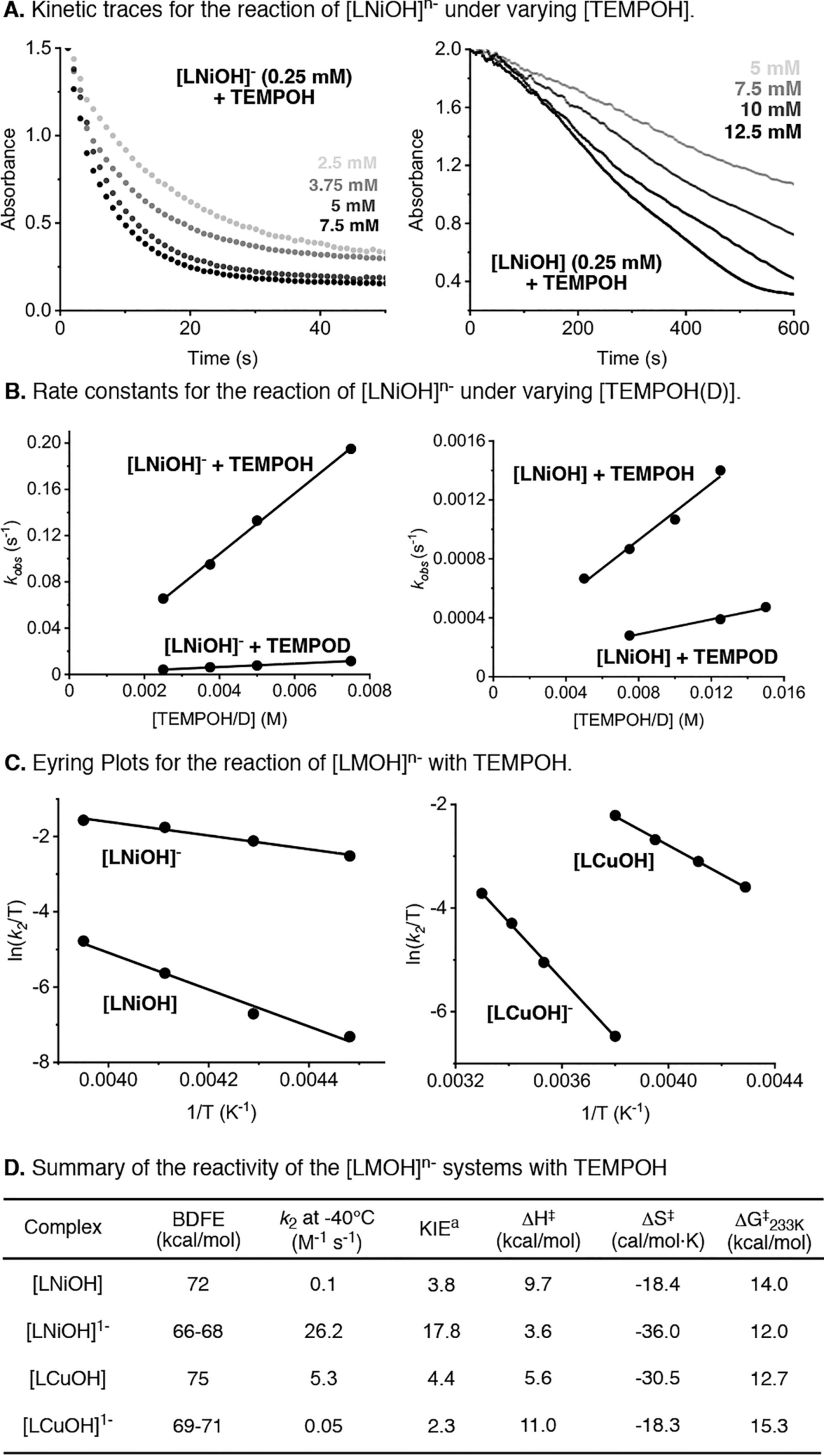
Kinetic analysis of the reaction between [LMOH]^*n*−^ and TEMPOH, including the kinetic traces (A), rate constants at different TEMPOH(D) concentrations (B), Eyring plots (C), and summary table (D). See the SI for further details.

The kinetics of the reaction between the [LNiOH]^*n*−^ complexes and TEMPOH was analyzed at −40 °C in DMF under pseudo-first-order conditions ([Ni] = 0.25 mM; [TEMPOH] = 2.5–12.5 mM; see the SI). For [LNiOH]^−^, we observed a fast decay of the UV–vis features of the Ni-semiquinone species, which were fitted to an exponential decay that allowed for calculating the pseudo-first-order kinetic constant *k*_obs_ (Figure 12). Variations on the concentration of TEMPOH led to a linear increase of the reaction rates, allowing us to obtain a second-order rate constant (*k*_2_ = 26.2 M^–1^ s^–1^). The kinetics for the reaction of [LNiOH] with TEMPOH were also evaluated at −40 °C. The initial decay of UV–vis features of [LNiOH] was fitted to a linear function, by the method of the initial rates, to determine the pseudo-first-order kinetic constant. The second-order rate constant for the reaction between [LNiOH] and TEMPOH was significantly lower than the *k*_2_ observed for [LNiOH]^−^ (0.1 vs 26.2 M^–1^ s^–1^). The kinetics of the reaction between the Cu analogues and TEMPOH were also studied (see the SI). Our results indicated that the reaction between TEMPOH and [LCuOH] was significantly faster than the reaction with [LCuOH]^−^ (*k*_2_ = 5.3 vs 0.05 M^–1^ s^–1^).

Considering the BDFE of the 1H^+^/1e^–^ reductive protonation of [LNiOH], [LNiOH]^−^, [LCuOH], and [LCuOH]^−^, we expected that the complexes with higher thermodynamic driving force (e.g., [LNiOH] and [LCuOH]) would react with TEMPOH faster than the complexes with lower driving forces ([LNiOH]^−^ and [LCuOH]^−^). Hence, we assumed that the *k*_2_ for the reaction between TEMPOH and the [LMOH]^*n*−^ complexes would follow the trend [LCuOH] > [LNiOH] ≫ [LCuOH]^−^ > [LNiOH]^−^. However, the reaction rates observed for [LNiOH]^−^ were unexpectedly high. Analysis of the kinetics at different temperatures allowed for obtaining the activation parameters (i.e., Δ*H*^⧧^, Δ*S*^⧧^, and Δ*G*^⧧^_233 K_) for the PCET transformations (Figure 12C). For all of the complexes, similar Δ*S*^⧧^ values were obtained (Δ*S*^⧧^ between −20 and −30 cal/mol·K) and the low Δ*G*^⧧^_233 K_ of [LNiOH]^−^ can be attributed to its low Δ*H*^⧧^ when compared to the other complexes.

Analysis of the kinetics of the reaction between the [LMOH]^*n*−^ complexes and TEMPOD helped to elucidate the kinetic isotope effect (KIE) of the PCET transformations. A primary KIE was observed for all of the complexes, which is consistent with O–H cleavage of the substrate during the rate-determining step of the reaction and suggestive of one-step CPET processes.^32,33^ While modest primary KIEs were found for [LCuOH], [LNiOH], and [LCuOH]^−^ (KIE between 2.3 and 4.4), the KIE for [LNiOH]^−^ was very large (KIE = 17.8), suggestive of H-atom tunneling.^34^

**Figure 13:**
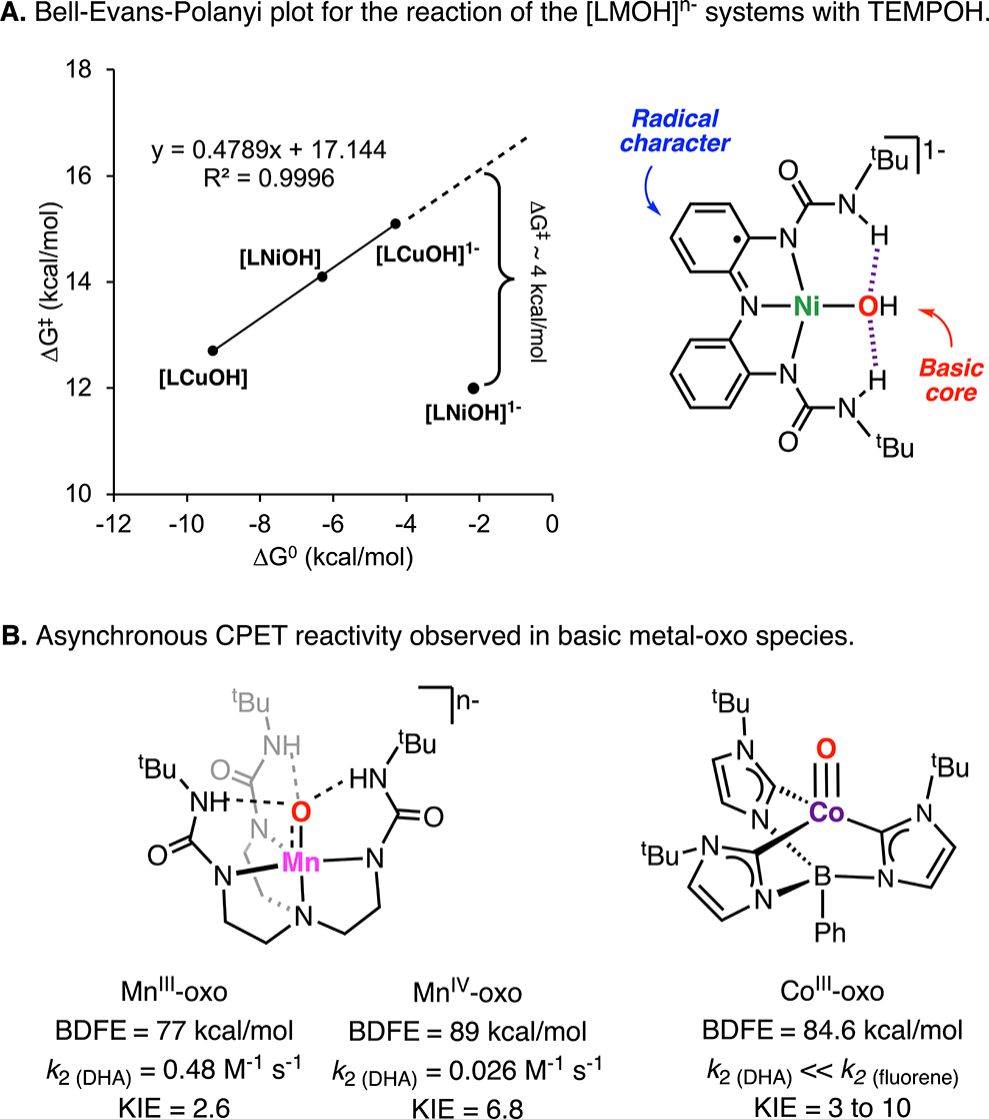
BEP plot for the reaction between [LMOH]^*n*−^ and TEMPOH (A) and selected examples of complexes where higher thermodynamic driving forces do not correlate to faster rates (B).

Overall, the kinetic evidence seems to indicate that the reaction mechanism by which [LNiOH]^−^ abstracts H atoms from TEMPOH differs from the other [LMOH]^*n*−^ complexes. In the Bell–Evans–Polanyi (BEP) plot for the reaction between the [LMOH]^*n*−^ complexes (Δ*G*^⧧^ vs Δ*G*^0^), we observed that [LCuOH], [LNiOH], and [LCuOH]^−^ produced a linear regression with a slope ∼0.5, characteristic of synchronous CPET transformations (Figure 13). The BEP plot clearly shows the deviation of [LNiOH]^−^ from the trend, also suggesting that the H-atom transfer for this NiOH complex may be driven by additional factors (see discussion below).

### Contextualization of Our Findings

In 2009, Borovik and co-workers described the H-atom-abstraction reactivity of a Mn^III^-oxo and Mn^IV^-oxo toward DHA (Figure 13B).^35^ The authors found that the reaction rates for the Mn^III^-oxo species were substantially higher than the one for the Mn^IV^-oxo, despite the latter having a higher thermochemical driving force for PCET (77 vs 89 kcal/mol). Initially, the authors suggested a change in the PCET mechanism due to the high basicity of the Mn^III^-oxo, with the Mn^IV^-oxo species reacting via a concerted CPET and the Mn^III^-oxo reacting via a stepwise PT–ET mechanism. However, recent publications from the same research group suggest that both Mn-oxo complexes react in an asynchronous CPET fashion in which the activation energy of the PCET transformation is highly affected by the basicity of the Mn-oxo species and the p*K*_a_ of the substrate oxidized.^16,36^ In a seminal research report, Anderson and co-workers found that the reactivity of a terminal Co^III^-oxo complex toward C–H bonds was also dictated by the basicity of the metal-oxo complex (Figure 13B).^17^ The authors reported that the reaction rates for the oxidation of substrates with similar BDFE (e.g., DHA and 9-fluorene) were highly dependent on the p*K*_a_ of the substrate (higher acidity of 9-fluorene led to higher reaction rates). In a subsequent study, the authors described that this asynchronous CPET transformation had also very high KIEs, suggesting that the tunneling effect is not exclusive to synchronous CPET transformations.^34^

Theoretical investigations by Srnec and co-workers have recently shown that some of the deviations on the BEP plots can be quantified using the nonclassical thermodynamic factors asynchronicity (η) and frustration (σ), which arise from differences in the reduction potentials and p*K*_a_ of all of the species depicted in the square scheme of a PCET transformation.^14,15^ However, the authors noted that their model did not fully account for nonthermodynamic factors that also affect the activation energy of PCET transformations, such as sterics, adiabaticity, H-atom tunneling, and the involvement of different spin states and excited states. The fact that the thermochemical parameters for the 1H^+^/1e^–^ reductive protonation [LNiOH]^−^ and [LCuOH]^−^ are similar (e.g., p*K*_a_ for protonation of [LNiOH]^−^ and [LCuOH]^−^ are ∼10 in both cases and *E*_1/2_ for the reduction of [LNiOH]^−^ and [LCuOH]^−^ only differ by 80 mV) suggest that the marked difference in the kinetics for PCET might arise from nonthermodynamic factors. We believe that the unique stereoelectronic structure of [LNiOH]^−^, the only complex that combines the high basicity of the hydroxide core and the radical character of the semiquinone-like ligand, might be a contributor to lowering the activation energy of the PCET reaction. The radical character of metal-oxo complexes (“oxyl character”) explains the high reactivity of some biological and synthetic oxidants, including Ni-oxyl complexes.^37^ Similarly, Ni^II^-iminyl radicals (as opposed to Ni^III^-imino species) have also been invoked in challenging Ni-mediated C–H amination reactions.^38^

## Conclusions

In this research paper, we described the synthesis and characterization of a mononuclear NiOH core stabilized by a tridentate redox-active ligand containing H-bonding donors. The NiOH complex was found to reach three molecular oxidation states (namely, [LNiOH]^2–^, [LNiOH]^−^, and [LNiOH]), all of which are described as Ni^II^OH species bound by different oxidation states of the ligand. The PCET reactivity of the [LNiOH]^*n*−^ complexes was studied in detail. The quinone-like complex [LNiOH] was found to act as a 2H^+^/2e^–^ oxidant, capable of performing H-atom abstraction from O–H and N–H substrates with BDFEs lower than 72 kcal/mol. Conversely, the semiquinone-like species [LNiOH]^−^ reacted as a 1H^+^/1e^–^ oxidant toward substrates with lower BDFE (∼66 kcal/mol). Unexpectedly, the reaction rates for the 1H^+^/1e^–^ dehydrogenation of TEMPOH were substantially faster for [LNiOH]^−^ (∼250 times) despite [LNiOH] being a stronger 1H^+^/1e^–^ acceptor (ΔBDFE ∼ 5 kcal/mol). This anomalous behavior, which might be a manifestation of asynchronous PCET, is hypothesized to be derived from the unique electronic properties of [LNiOH]^−^, which combines a noncoupled ligand-based radical (putative electron acceptor) with a highly basic NiOH core (putative proton acceptor).

## Experimental Section

### Materials

All reagents were purchased from commercial suppliers and used as received except as noted. [LCuOH]^2–^ was prepared as previously reported.^10^ DMF·CF_3_SO_3_H was prepared by combining equimolar amounts of DMF and CF_3_SO_3_H in DCM for 30 min at 0 °C. All solvents were purchased at the highest level of purity and further purified and dried by passing through an activated alumina solvent purification system (MB SPS-7, MBRAUN INERTGAS-SYSTEME, Garching, Germany). Dimethylformamide (DMF) was distilled under partial vacuum before use. Deuterated solvents were purchased from Cambridge Isotope Laboratories (Tewksbury, MA) and used as received.

### Physical Methods

Air-free handling of the copper and nickel complexes was performed inside a MBRAUN UNIlab Pro SP glovebox system with N_2_ working gas. Electrochemical measurements were performed using a CH Instruments 620E Electrochemical Workstation (CH Instruments, Austin, TX). UV–vis spectra were collected using a Hewlett-Packard 8454 diode-array spectrophotometer with a 1-cm-path-length quartz cell. The spectrometer was equipped with Agilent UV–visible ChemStation software (ver. B.05.02 [16], Agilent Technologies, Santa Clara, CA) and a Unisoku CoolSpeK UV cryostat (UNISOKU Co., Hirakata, Japan). NMR spectra were on a 500 MHz NMR spectrometer (NEO 500 or Avance III, Bruker Corp., Billerica, MA) to acquire spectra with 16 cumulative scans. Evans method experiments were performed using a 7 in., 5-mm-o.d. NMR tube with a smaller 3-mm-o.d. NMR tube inserted inside. The outer tube contained the analyte dissolved in a deuterated solvent with a dichloromethane (DCM) internal standard. The smaller inner tube contained the same deuterated solvent and DCM internal standard solution (without analyte). X-band EPR spectra of frozen solutions were recorded on a Bruker ELEXSYS spectrometer equipped with an Oxford liquid-helium cryostat and a Bruker bimodal cavity. The quantification of all signals was measured relative to a CuEDTA spin standard prepared from a copper atomic absorption standard (Sigma-Aldrich, St. Louis, MO). The spectra were recorded under nonsaturating power conditions. The microwave frequency was calibrated with a frequency counter, and the magnetic field was measured with an NMR gaussmeter. The sample temperature was calibrated against a calibrated CX-1050 Cernox sensor (Lake Shore Cryotronics, Westerville, OH) mounted inside an EPR tube. A modulation amplitude of 1 mT and frequency of 100 kHz was used for all EPR spectra. CHN analysis was performed by Midwest Micro Lab (Indianapolis, IN). A PerkinElmer Frontier FT-IR spectrometer with an attenuated-total-reflectance attachment containing a germanium crystal was used. Spectra were obtained over a range of 4000–700 cm^–1^ with 0.4 cm^–1^ resolution.

### Synthesis and Characterization of [LNiOH]^2–^

This complex was prepared using a method adapted from our previous publication.^1^ In the glovebox, ligand LH_3_ (100 mg, 0.25 mmol) was dissolved in DMF (1 mL) in an 8 mL vial with a stir bar. KH (30 mg, 0.75 mmol) was added to the solution and stirred for 30 min or until hydrogen evolution ceased. Ni(OAc)_2_ (45 mg, 0.25 mmol) was added as a solid and allowed to stir for 3 h. Me_4_NOH·5H_2_O (90 mg, 0.50 mmol) was added as a solid and allowed to react for 6 h to yield a dark-green solution. Et_2_O (20 mL) was used to precipitate the complex as a green powder. The precipitate was dried, filtered, redissolved in DMF (1 mL), and recrystallized by vapor diffusion with Et_2_O. Green crystals of [LNiOH]^2–^ were obtained in 40% yield (65 mg).

Elemental analysis: (C_30_H_53_N_7_NiO_3_). Experimental C: 58.06%, H: 9.12%, N: 15.87%. Calculated C: 58.26%, H: 8.64%, N: 15.85%. FT-IR (cm^–1^, selected bands): 3029, 2960, 2921, 1590, 1541, 1492, 1433, 1345, 1296, 1287, 1228, 1110, 1031, 944, 727. Crystals suitable for X-ray structure determination were obtained by layering Et_2_O on a solution of complex [LNiOH]^2–^ in DMF (see additional details in the SI).

### Cyclic Voltammetry of [LNiOH]^2–^

A total of 3 mL of a DMF solution of [LNiOH]^2–^ (1 mM) containing 0.1 M NBu_4_PF_6_ was prepared in the glovebox and transferred via a syringe to an argon-purged electrochemical cell outside. A glassy carbon working electrode, CHI112 Ag/AgNO_3_ (0.1 M) reference electrode with a porous Teflon tip, and Pt wire counter electrode were used. The potentials were measured with respect to the Ag/AgNO_3_ reference electrode and converted to Fc^0/+^ after running a cyclic voltammogram of Fc under the same conditions. The cyclic voltammogram was recorded at a scan rate of 100 mV/s, carried out under an argon atmosphere.

### UV–Vis Characterization of [LNiOH]^2–^, [LNiOH]^−^, and [LNiOH]

A total of 3 mL solutions of [LNiOH]^2–^ was prepared in DMF (0.25 mM) inside a N_2_-filled glovebox. The addition of stoichiometric amounts of oxidant (FcPF_6_) solution generated the respective high-valent oxidant states [LNiOH]^−^ and [LNiOH]. They can also be reduced back to [LNiOH]^2–^ by the addition of stoichiometric amounts of a reductant (CoCp_2_) solution. The UV–vis spectra were recorded using a Schlenk quartz cuvette with a rubber septum and magnetic stirring under an argon flow at room temperature (see further details in the SI).

### EPR Characterization of [LNiOH]^2–^, [LNiOH]^−^, and [LNiOH]

In the glovebox, a stock solution of [LNiOH]^2–^ (1 mM) in DMF was prepared along with a stock solution of FcPF_6_ (1 mM). Differing ratios of complex and oxidant were added such that all of the final solutions consisted of 0.3 mL of a 1 mM solution of [LNiOH]^2–^, [LNiOH]^−^, and [LNiOH] and were thoroughly mixed. Each solution was injected into an EPR tube and immediately frozen in liquid N_2_. Note that [LNiOH]^2–^ and [LNiOH] had no observable signal in the standard perpendicular mode (see further details in the SI).

### NMR Characterization of [LNiOH]^2–^, [LNiOH]^−^, and [LNiOH]

In the glovebox, solutions of [LNiOH]^2–^, [LNiOH]^−^, and [LNiOH] were prepared in DMF-*d*_7_ (10 mM) with requisite amounts of FcPF_6_ oxidant and transferred into an NMR tube. All samples were recorded at room temperature (see the SI for additional details).

### Evans Method Measurements

A DMF-*d*_7_ solution containing DCM (∼100 mM) and [LNiOH]^*n*−^ (10 mM) was placed in an NMR tube. A coaxial inner tube containing the same solvent (DMF-*d*_7_ with DCM) was placed inside the standard NMR tube, and NMR spectra were taken at room temperature (298 K). The difference in the internal standard resonances was based on the shift of DCM peaks in the presence of a paramagnetic material (see the SI for additional details).

### PCET Reactivity

#### Analysis of the Stoichiometry

***UV–vis experiments:*** In a typical experiment, 3 mL of a [LMOH]^*n*−^ solution (0.25 mM) in DMF was placed in a 10-mm-path-length quartz cell with a stir bar, capped with a rubber septum. All reactivity experiments were performed at room temperature (25 °C). Differing amounts of a solution of FcPF_6_ (0.25 mM or 0.5 mM) were injected into the complex solution to generate the corresponding “high-valent” species [LMOH]^−^ and [LMOH]. DMF solutions of substrate (5 mM for TEMPOH or 25 mM for PhNHNHPh) or DMF·CF_3_SO_3_H (0.25 mM to 0.5 mM) were stored in 500 μL gastight syringes and injected into the quartz cell correspondingly. ***EPR experiments:*** In the glovebox, 300 μL solutions of [LMOH]^*n*−^ (1 mM), requisite amounts of FcPF_6_ (1 or 2 mM), requisite amounts of DMF·CF_3_SO_3_H (0–2 mM), and TEMPOH (20 mM) were mixed, and the mixture was allowed to incubate for 60 min. They were then transferred to EPR tubes and rapidly transferred outside the glovebox to be frozen in liquid N_2_. ***NMR experiments:*** In the glovebox, 1.0 mL of a DMF-*d*_7_ solution of [LNiOH]^2–^ (10 mM), FcPF_6_ (10 mM or 20 mM), hexamethylbenzene (10 mM, internal standard), and substrate (40–50 mM) were combined, and the mixture was transferred to a 7-in., 5-mm-o.d. NMR tube, which was capped and sealed before NMR spectra were taken (see additional details in the SI).

#### Analysis of the Kinetics

***UV–vis experiments:*** In a typical reaction, 2.7 mL of a DMF solution of [LMOH]^*n*−^ (0.25 mM) was transferred to a 10-mm-path-length quartz cell with a stir bar and a rubber septum. Differing amounts of a solution of FcPF_6_ (0.25 mM or 0.5 mM) were injected into the complex solution to generate the corresponding “high-valent” species [LMOH]^−^ and [LMOH]. DMF solutions of substrate (5 mM for TEMPOH) were stored in 500 μL gastight syringes and injected into the quartz cell correspondingly. All reactions were run under an Ar flow and at −40 °C, with the exception of [LCuOH]^−^, which was performed at room temperature. The decays of the [LMOH]^*n*−^ bands were monitored by UV–vis, which were fitted to the following exponential function.

### DFT Calculations

All DFT calculations were performed with the Amsterdam Density Functional (ADF)^39,40^ and *QUILD*(41) programs, and were performed using the unrestricted Kohn-Sham scheme. Molecular orbitals were expanded in an uncontracted set of Slater type orbitals (STOs) of triple-ζ quality with double polarization functions (TZ2P), or the TDZP basis set which consists of triple-ζ quality on the metal and double-ζ quality on all other atoms, in both cases including one polarization function.^41,42^ Core electrons were not treated explicitly during the geometry optimizations (frozen core approximation^40^). An auxiliary set of s, p, d, f, and g STOs was used to fit the molecular density and to represent the coulomb and exchange potentials accurately for each SCF cycle.

Geometries of all possible spin states were optimized with the *QUILD*(41) program using adapted delocalized coordinates until the maximum gradient component was less than 10–4 a.u. Energies, gradients and Hessians^43^ (for vibrational frequencies) were calculated using S12g,^44,45^ in all cases by including solvation effects through the COSMO dielectric continuum model with appropriate parameters for the solvents.^46,47^ For computing Gibbs free energies, all small frequencies were raised to 100 cm^–1^ in order to compensate for the breakdown of the harmonic oscillator model.^48,49^ Scalar relativistic corrections have been included self-consistently in all calculations by using the zerothorder regular approximation (ZORA^50^). The geometry optimizations (with the TZ2P basis set) have been performed with S12g with a Becke grid of VeryGood quality. See additional details in the Supporting Information.

## Supporting Information Available

Experimental details, including characterization data, spectra, and DFT calculations (PDF)
All computational data (ZIP)
