# Supplementary material for: Enhanced Proton-Coupled Electron-Transfer Reactivity by a Mononuclear Nickel(II) Hydroxide Radical Complex
Source: Inorg Chem. 2024 Dec 16;63(52):24453–65. doi: 10.1021/acs.inorgchem.4c03370 (PMC11688665; doi:10.1021/acs.inorgchem.4c03370)
Supplement: Supplementary file 1 — ic4c03370_si_001.pdf [file ic4c03370_si_001.pdf]

Supporting Information

for

Enhanced PCET Reactivity by a Mononuclear Nickel(II)-Hydroxide Radical Complex

Daniel Ye[†], Tong Wu[†], Ankita Puri[†], David D. Hebert[†], Maxime A. Siegler[#], Michael P. Hendrich[*,†], Marcel Swart[*, Ψ], and Isaac Garcia-Bosch[†,*]

[†]*Department of Chemistry, Carnegie Mellon University, Pittsburgh, Pennsylvania 15213, United States.*

[#]*Johns Hopkins University, Baltimore, Maryland 21218, United States.*

[Ψ] *University of Girona, Campus Montilivi (Ciències), IQCC, Girona, Spain; ICREA, Pg. Lluís Companys 23, 08010, Barcelona, Spain.*

Contents

1. Physical methods and materials 3
2. Synthesis and characterization of $[LNiOH]^{2-}$ 5
3. Electrochemistry 8
4. PCET reactivity: Stoichiometry 15
5. PCET reactivity: Thermochemistry 39
6. PCET reactivity: Kinetics 46
7. DFT Calculations 66
8. References 68

1. Physical methods and materials

Reagents and solvents: All reagents were purchased from commercial suppliers and used as received except as noted. **[LCuOH]$^{2-}$** was prepared as previously reported.[1] DMF·CF_3SO_3H was prepared by combining equimolar amounts of DMF and CF_3SO_3H in DCM for 30 minutes at 0 °C.[2] All solvents were purchased at the highest level of purity and further purified and dried by passing through an activated alumina solvent purification system (MB SPS-7, M. BRAUN INERTGAS-SYSTEME, Garching, Germany). Dimethylformamide (DMF) was distilled under partial vacuum before use. Deuterated solvents were purchased from Cambridge Isotope Laboratories (Tewksbury, MA, USA) and used as received.

Glovebox: Air-free handling of the copper and nickel complexes was performed inside a MBRAUN UNIlab Pro SP glovebox system with N_2 working gas.

Electrochemistry: Electrochemical measurements were performed using a CH Instruments 620E Electrochemical Workstation (CH Instruments, Austin, TX, USA).

UV-vis Spectroscopy: UV-vis spectra were collected using a Hewlett Packard 8454 diode array spectrophotometer with a 1 cm path quartz cell. The spectrometer was equipped with Agilent UV-Visible ChemStation software (ver. B.05.02 [16], Agilent Technologies, Santa Clara, CA, USA) and a Unisoku CoolSpeK UV cryostat (UNISOKU Co., Hirakata, Japan).

NMR Spectroscopy: NMR spectra were on a 500 MHz NMR spectrometer (NEO 500 or Avance III, Bruker Corp., Billerica, MA) to acquire spectra with 16 cumulative scans. Evans method experiments were performed using a 7 in, 5 mm o.d. NMR tube with a smaller 3 mm o.d. NMR tube inserted inside. The outer tube contained the analyte dissolved in a deuterated solvent with a dichloromethane (DCM) internal standard. The smaller inner tube contained the same deuterated solvent and DCM internal standard solution (without analyte).

EPR Spectroscopy: X-band EPR spectra of frozen solutions were recorded on a Bruker ELEXSYS spectrometer equipped with an Oxford liquid helium cryostat and a Bruker bimodal cavity. The quantification of all signals was measured relative to a CuEDTA spin standard prepared from a copper atomic absorption standard (Sigma-Aldrich, St. Louis, MO, USA). The spectra were recorded under non-saturating power conditions. The microwave frequency was calibrated with a frequency counter, and the magnetic field was measured with an NMR gaussmeter. The sample temperature was calibrated against a calibrated CX-1050 Cernox sensor (Lake Shore Cryotronics, Westerville, OH) mounted inside an EPR tube. A modulation amplitude of 1 mT and frequency of 100 kHz was used for all EPR spectra.

Elemental Analysis: CHN analysis was performed by Midwest Micro Lab (Indianapolis, IN, USA).

Fourier-Transform Infrared Spectroscopy (FT-IR): A Perkin Elmer Frontier FT–IR Spectrometer with an attenuated total reflectance attachment containing a germanium crystal was used. Spectra were obtained over a range of 4000–700 cm–1 with 0.4 cm–1 resolution.

2. Synthesis and characterization of $[LNiOH]^{2-}$

This complex was prepared using a method adapted from our previous publication.[1] In the glovebox, ligand LH_3 (100 mg, 0.25 mmol) was dissolved in DMF (1 mL) in an 8 mL vial with a stir bar. KH (30 mg, 0.75 mmol) was added to the solution and stirred for 30 minutes or until hydrogen evolution ceased. $Ni(OAc)_2$ (45 mg, 0.25 mmol) was added as a solid and allowed to stir for 3 hours. $Me_4NOH•5H_2O$ (90 mg, 0.50 mmol) was added as a solid and allowed to react for 6 hours to yield a dark green solution. Et_2O (20 mL) was used to precipitate the complex as a green powder. The precipitate was dried, filtered, redissolved in DMF (1 mL), and recrystallized by vapor diffusion with Et_2O. Green crystals of **$[LNiOH]^{2-}$** were obtained in 40% yield (65 mg).

Elemental analysis: ($C_{30}H_{53}N_7NiO_3$) Experimental C: 58.06%, H: 9.12%, N: 15.87%. Calculated C: 58.26%, H: 8.64%, N: 15.85%.

FT-IR (cm^{-1}, selected bands): 3029, 2960, 2921, 1590, 1541, 1492, 1433, 1345, 1296, 1287, 1228, 1110, 1031, 944, 727

Crystals suitable for X-ray structure determination were obtained by layering Et_2O on a solution of complex **$[LNiOH]^{2-}$** in DMF.

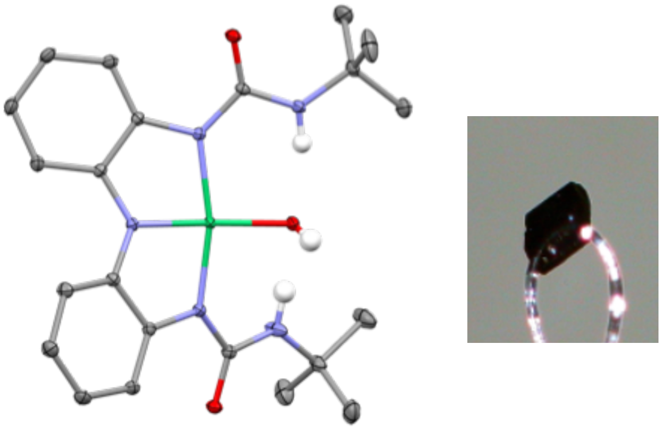

Figure S1. Left: Displacement ellipsoid plot (50% probability level) of **$[LNiOH]^{2-}$** at 110(2) K. H atoms (except for N–H and O–H) were removed for clarity. Right: Crystal used for data collection.

Single-crystal X-ray Diffraction Crystallography: All reflection intensities were measured at 110(2) K using a SuperNova diffractometer (equipped with Atlas detector) with Mo Kα radiation (λ = 0.71073 Å) under the program CrysAlisPro (Version CrysAlisPro 1.171.39.29c, Rigaku OD, 2017). The same program was used to refine the cell dimensions and for data reduction. The structure was solved with the program SHELXS-2018/3 (Sheldrick, 2018) and was refined on F^2 with

SHELXL-2018/3 (Sheldrick, 2018). Numerical absorption correction based on gaussian integration over a multifaceted crystal model was applied using CrysAlisPro. The temperature of the data collection was controlled using the system Cryojet (manufactured by Oxford Instruments). The H atoms were placed at calculated positions (unless otherwise specified) using the instructions AFIX 43 or AFIX 137 with isotropic displacement parameters having values 1.2 or 1.5 U_{eq} of the attached C atoms. The H atoms attached to N1, N5, O3/O3' were found from different Fourier maps, and their coordinates were refined pseudofreely using the DFIX instruction in order to keep the N–H and O–H bonds within acceptable ranges. The structure is partly disordered. The Ni–OH fragment of the Ni complex and the two tetramethylammonium cations are disordered over two orientations. All occupancy factors can be retrieved from the final .cif file. The asymmetric unit contains also one disordered DMF lattice solvent molecule, which is found at site of inversion symmetry, and its occupancy factor is constrained to be 0.5. The asymmetric unit also contains one lattice Et_2O solvent molecule that is found to be very disordered (and most likely partially occupied). In the final refinement, its contribution has been removed using the SQUEEZE procedure in Platon (see A.L. Spek, *Acta Cryst.* 2009, D65, 148-155).

Table S1. Crystallographic data for **[LNiOH]$^{2-}$**

Crystal data	
Chemical formula	$2(C_{22}H_{29}N_5NiO_3)\cdot C_3H_7NO\cdot 4(C_4H_{12}N)$
M_r	1310.10
Crystal system, space group	Monoclinic, $P2_1/c$
Temperature (K)	110
a, b, c (Å)	14.7927 (3), 11.5227 (2), 22.2664 (4)
β (°)	90.5607 (19)
V (Å^3)	3795.17 (12)
Z	2
Radiation type	Mo $K\alpha$
μ (mm^{-1})	0.55
Crystal size (mm)	0.34 × 0.26 × 0.15
Data collection	
Diffractometer	SuperNova, Dual, Cu at zero, Atlas
Absorption correction	Gaussian<br>*CrysAlis PRO* 1.171.39.29c (Rigaku Oxford Diffraction, 2017) Numerical absorption correction based on gaussian integration over a multifaceted crystal model Empirical absorption correction using spherical harmonics, implemented in SCALE3 ABSPACK scaling algorithm.
T_{min}, T_{max}	0.678, 1.000
No. of measured, independent and observed [$I > 2\sigma(I)$] reflections	57184, 8714, 7696
R_{int}	0.026
(sin θ/λ)$_{max}$ (Å^{-1})	0.650
Refinement	
$R[F^2 > 2\sigma(F^2)]$, $wR(F^2)$, S	0.033, 0.088, 1.05
No. of reflections	8714
No. of parameters	556
No. of restraints	343
H-atom treatment	H atoms treated by a mixture of independent and constrained refinement
$\Delta\rho_{max}$, $\Delta\rho_{min}$ (e Å^{-3})	0.39, -0.24

3. Electrochemistry

3.1. Cyclic voltammetry

3 mL of a DMF solution of **[LNiOH]$^{2-}$** (1 mM) containing 0.1 M NBu_4PF_6 was prepared in the glovebox and transferred via a syringe to an argon-purged electrochemical cell outside. A glassy carbon working electrode, CHI112 Ag/$AgNO_3$ (0.1 M) reference electrode with a porous Teflon tip, and Pt wire counter electrode were used. The potentials were measured with respect to the Ag/$AgNO_3$ reference electrode and converted to $Fc^{0/+}$ after running a cyclic voltammogram of Fc under the same conditions. The cyclic voltammogram was recorded at a scan rate of 100 mV/s, carried out under an argon atmosphere.

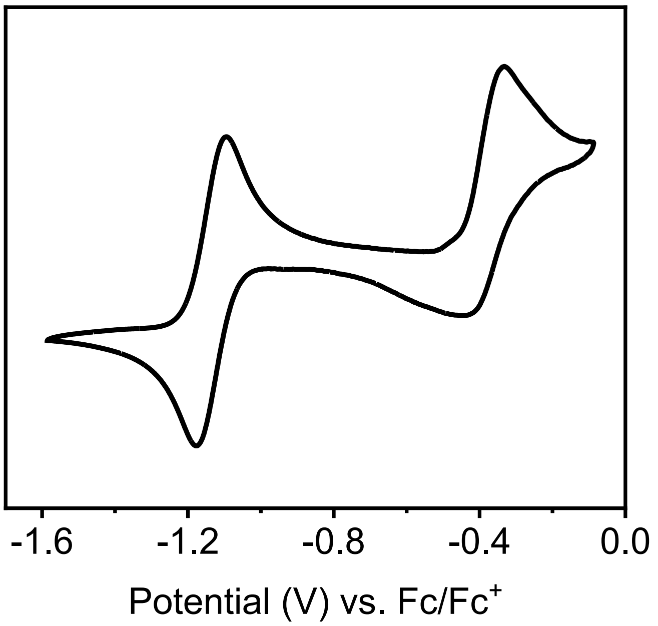

Figure S2. Cyclic voltammogram of **[LNiOH]$^{2-}$.** [Ni] = 1 mM, [NBu_4PF_6] electrolyte = 0.1 M, scan rate: 100 mV/s.

3.2. UV-vis characterization

3 mL solutions of **[LNiOH]$^{2-}$** were prepared in DMF (0.25 mM) inside a N_2 filled glovebox. Addition of stoichiometric amounts of oxidant ($FcPF_6$) solution generated the respective high valent oxidant states **[LNiOH]$^{-}$** and **[LNiOH]**. They can also be reduced back to **[LNiOH]$^{2-}$** by addition of stoichiometric amounts of reductant ($CoCp_2$) solution. The UV-vis spectra were recorded using a Schlenk quartz cuvette with a rubber septum and magnetic stirring under argon flow, at room temperature.

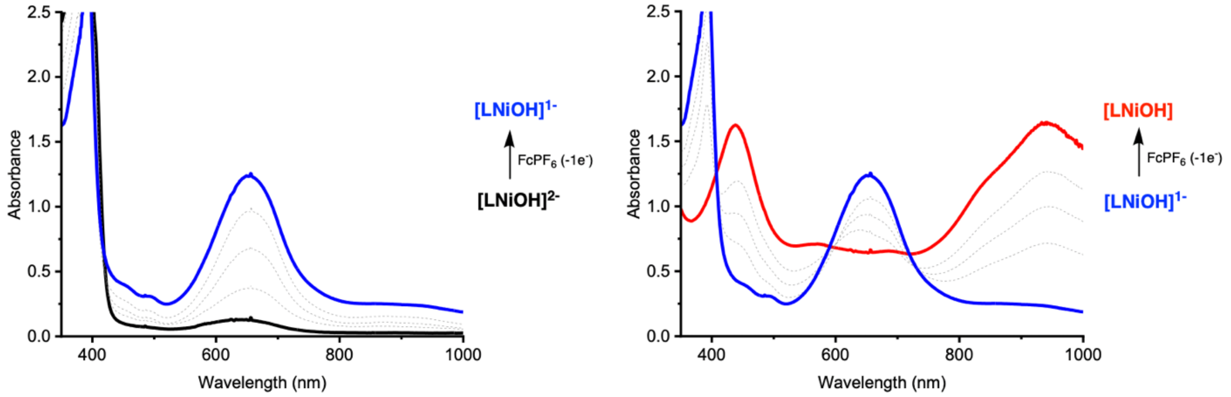

Figure S3. UV-vis spectra for the oxidation of **[LNiOH]$^{2-}$** to **[LNiOH]$^{-}$** (left) and **[LNiOH]$^{-}$** to **[LNiOH]** (right) upon addition of $FcPF_6$.

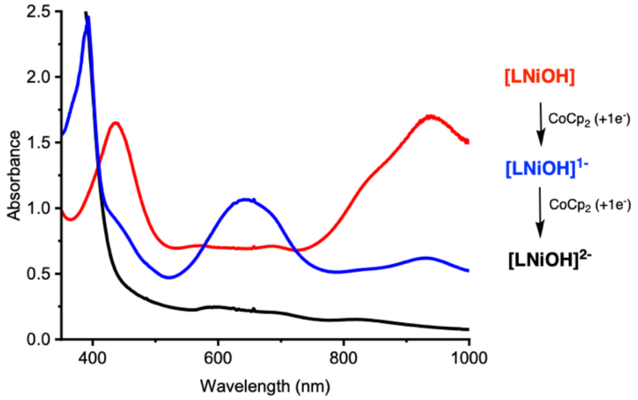

Figure S4. UV-vis spectra for the reduction of **[LNiOH]** to **[LNiOH]$^{-}$** and **[LNiOH]$^{2-}$** upon addition of $CoCp_2$. Note: Some of the weak UV-vis bands that appeared upon regeneration of **[LNiOH]$^{2-}$** might belong to the metallocene reagents utilized.

3.3. EPR characterization

In the glovebox, a stock solution of **[LNiOH]$^{2-}$** (1 mM) in DMF was prepared along with a stock solution of $FcPF_6$ (1 mM). Differing ratios of complex and oxidant were added such that all of the final solutions consisted of 0.3 mL of a 1 mM solution of **[LNiOH]$^{2-}$**, **[LNiOH]$^{-}$**, and **[LNiOH]** and were thoroughly mixed. Each solution was injected into an EPR tube and immediately frozen in liquid N_2. Note that **[LNiOH]$^{2-}$** and **[LNiOH]** had no observable signal in the standard perpendicular mode, so only the spectrum of **[LNiOH]$^{-}$** is shown.

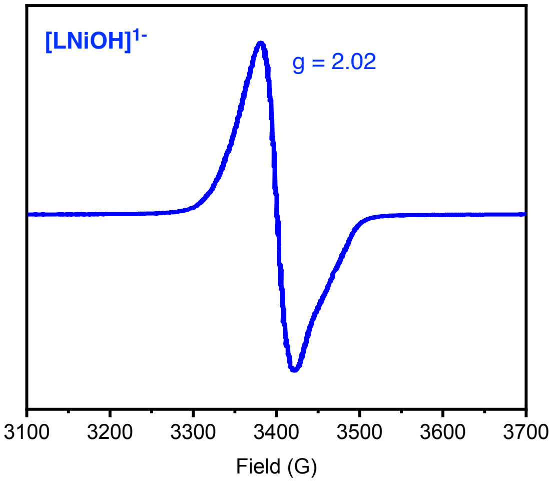

Figure S5. X-band EPR spectrum of **[LNiOH]$^{-}$**

3.4. NMR characterization

In the glovebox, solutions of **[LNiOH]$^{2-}$**, **[LNiOH]$^{-}$**, and **[LNiOH]** were prepared in DMF-d_7 (10 mM) with requisite amounts of $FcPF_6$ oxidant and transferred into an NMR tube. All samples were recorded at room temperature.

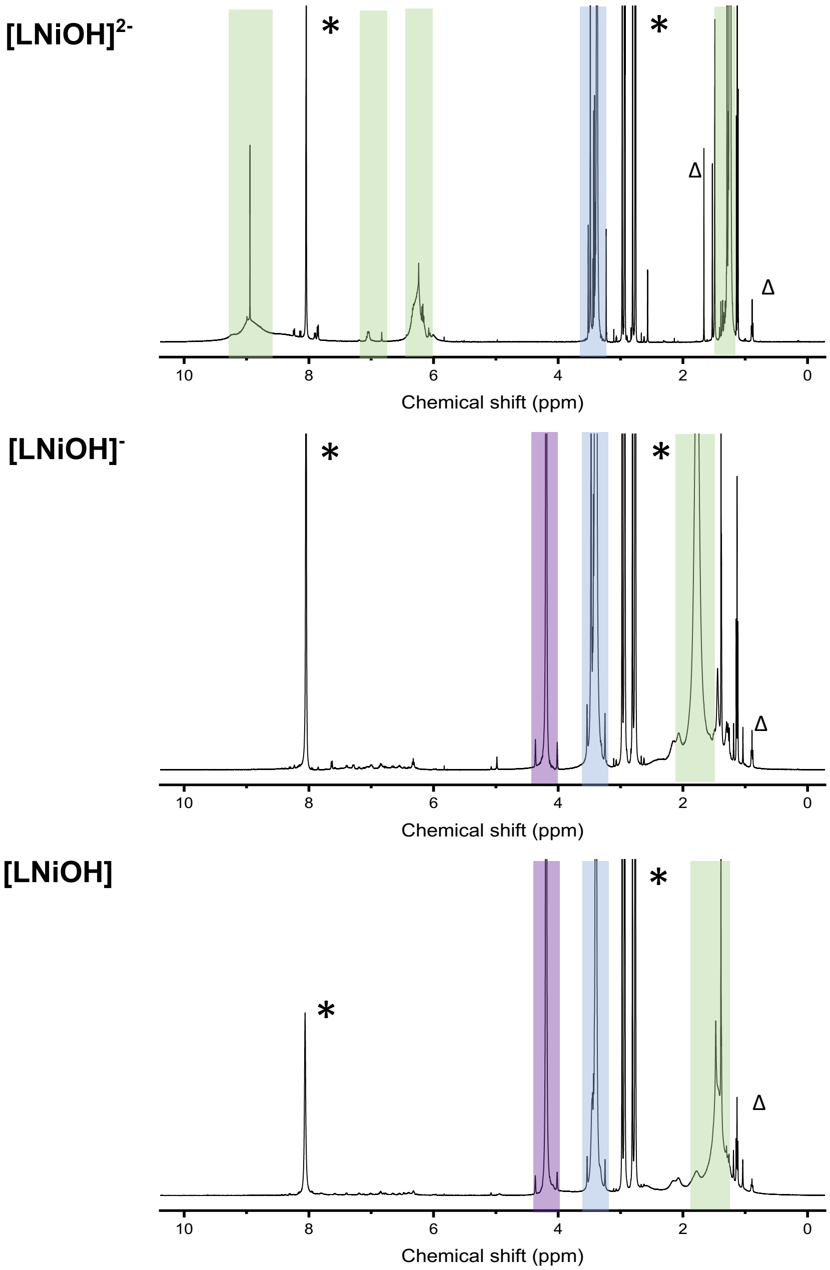

Figure S6. ^{1}H-NMR spectra of **[LNiOH]$^{2-}$**, **[LNiOH]$^{-}$**, and **[LNiOH]** in DMF-d_7. Complex peaks are highlighted in green, NMe_4^+ cation highlighted in blue, ferrocene highlighted in purple, DMF impurities denoted by *, and Et_2O, THF, and other trace impurities are denoted by Δ. Note: The broad peaks observed for the diamagnetic complex **[LNiOH]$^{2-}$** are similar to the spectrum reported by Borovik for a related diamagnetic complex $[(NNN^{3-})Ni^{II}OH_2]^{2-}$.[3]

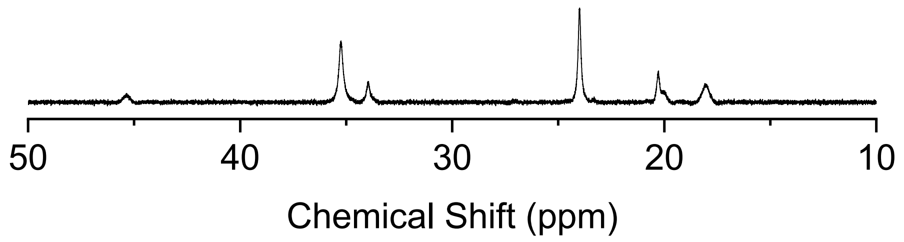

Figure S7. ^{1}H-NMR spectrum (paramagnetic mode) of **[LNiOH]** in DMF-d_7 at room temperature. Note: the integration of the paramagnetic peaks was compared to the integration of the ^{t}Bu groups (broad peak ~ 1.3 ppm, see Figure S6), leading to values between 0.5 and 1. The spectrum was recorded from 100 to -100 ppm but paramagnetic peaks only appeared between 10 and 50 ppm.

3.5. Evans method measurements

The Evans Method was used to determine the bulk magnetic susceptibility of each **[LNiOH]$^{n-}$** complex. The effective magnetic moment was calculated by a simplified Evans method analysis using the following equation:

$$\mu_{eff} = 0.0618\sqrt{\frac{\Delta\upsilon T}{2fM}}$$

where μ_{eff} is the effective magnetic moment, $\Delta\upsilon$ is the difference in frequency (Hz) between the internal standard resonances, *f* (MHz) is the frequency of the NMR spectrometer, T (K) is the temperature, and M is the molar concentration of the metal complex.

A DMF-d_7 solution containing DCM (~100 mM) and **[LNiOH]$^{n-}$** (10 mM) was placed in an NMR tube. A coaxial inner tube containing the same solvent (DMF-d_7 with DCM) was placed inside the standard NMR tube, and NMR spectra were taken at room temperature (298 K). The difference in internal standard resonances was based on the shift of DCM peaks in the presence of paramagnetic material.

Table S2. Effective magnetic moments for **[LNiOH]$^{n-}$** as determined by Evans Method in DMF-d_7 at 298 K.

Complex	μ_{eff} (μ_B)
**[LNiOH]$^{2-}$**	0
**[LNiOH]$^{-}$**	1.77
**[LNiOH]**	2.81

3.6. IR characterization

In the glovebox, stoichiometric amounts of FcPF6 were added to separate DMF solutions of **[LNiOH]$^{2-}$** (20 mM) to generate **[LNiOH]$^{-}$** and **[LNiOH]**. Removal of DMF via Biotage V10 evaporation yielded a dark blue powder for **[LNiOH]$^{-}$** and a dark green powder for **[LNiOH]**. Complexes **[LNiOH]$^{n-}$** (10 mg) were placed on the ATR crystal for detection.

[LNiOH]$^{2-}$ FT-IR (cm^{-1}, selected bands): 3029, 2960, 2921, 1590, 1541, 1492, 1433, 1345, 1296, 1287, 1228, 1110, 1031, 944, 727.

[LNiOH]$^{-}$ FT-IR (cm^{-1}, selected bands): 3375, 3029, 2968, 1673, 1602, 1573, 1495, 1456, 1387, 1358, 1299, 1270, 1240, 1211, 1123, 1035, 957, 849, 742.

[LNiOH] FT-IR (cm^{-1}, selected bands): 3382, 2972, 2894, 1660, 1611, 1503, 1454, 1396, 1356, 1298, 1259, 1200, 1141, 1040, 956, 838, 750.

4. PCET reactivity: Stoichiometry

4.1. Experimental procedures

UV-vis experiments: In a typical experiment, 3 mL of a **$[LMOH]^{n-}$** solution (0.25 mM) in DMF were placed in a 10 mm path quartz cell with a stir bar, capped with a rubber septum. All reactivity experiments were performed at room temperature (25 ^{o}C). Differing amounts of a solution of $FcPF_6$ (0.25 mM or 0.5 mM) were injected into the complex solution to generate the corresponding "high-valent" species **$[LMOH]^{-}$** and **[LMOH].** DMF solutions of substrate (5 mM for TEMPOH or 25 mM for PhNHNHPh) or $DMF{\cdot}CF_3SO_3H$ (0.25 mM to 0.5 mM) were stored in 500 μL gastight syringes and injected into the quartz cell correspondingly.

EPR experiments: In the glovebox, 300 μL solutions of **$[LMOH]^{n-}$** (1 mM), requisite amounts of $FcPF_6$ (1 or 2 mM), requisite amounts of $DMF{\cdot}CF_3SO_3H$ (0-2 mM), and TEMPOH (20 mM) were mixed, and the mixture was allowed to incubate for 60 minutes. They were then transferred to EPR tubes and rapidly transferred outside the glovebox to be frozen in liquid N_2.

NMR experiments: In the glovebox, 1.0 mL of a DMF-d_7 solution of **$[LNiOH]^{2-}$** (10 mM), $FcPF_6$ (10 mM or 20 mM), hexamethylbenzene (10 mM, internal standard I.S.), and substrate (40-50 mM) were combined, and the mixture was transferred to a 7-inch, 5-mm o.d. NMR tube, which was capped and sealed before NMR spectra were taken.

4.2. Summary of the acids and PCET reagents used

Table S3. Summary of the pK_a values for the various organic acids used in this paper.[4]

Reactant	pK_a (DMF)
4-MeO-2,6-DTBP	19
2,6-DTBP	18.2
4-NO_2-PhOH	12.3
4-NO_2-2,6-DTBP	8.6
DMF·CF_3SO_3H	6.1[a,4]

[a]In MeCN

Table S4. Summary of BDFE and reactivities for the substrates used in this paper.[5]

H atom donor	pK_a (DMF)	BDFE / kcal mol^{-1} (DMF)	[LNiOH] React	[LNiOH]$^-$ React	[LCuOH] React	[LCuOH]$^-$ React
DHA	-	72.9	No	No	No	No
2,4,6-TTBP	18.6	75.1	Yes	No	Yes	No
4-MeO-2,6-DTBP	19	71.9	Yes	No	Yes	No
TEMPOH	31	65.7	Yes	Yes	Yes	Yes
PhNHNHPh	26.7	65.2 (avg)	Yes	Yes	Yes	Yes

4.3. Proposed species formed in the PCET and protonation reactivity of [LNiOH]$^{n-}$

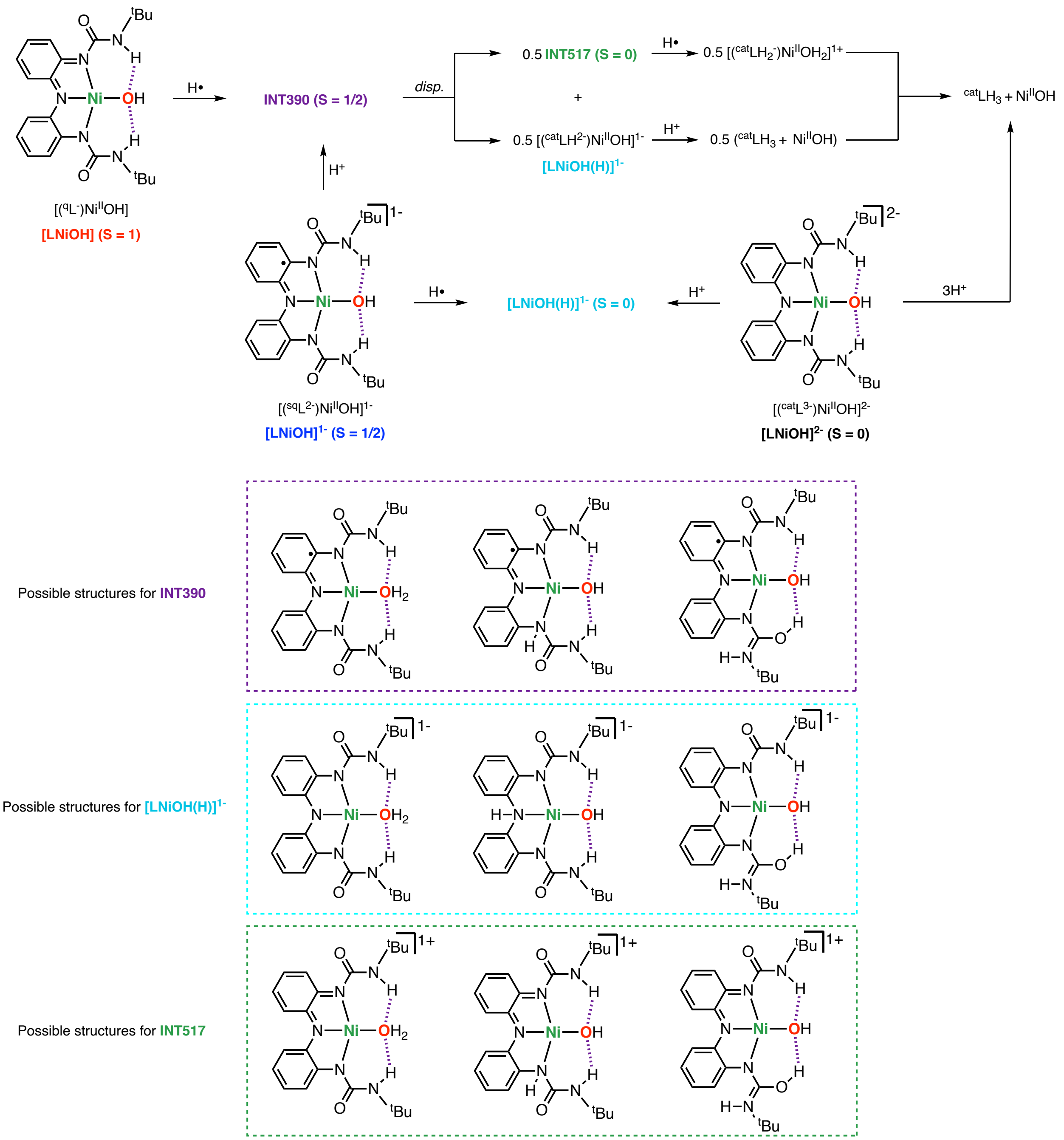

Figure S8. Proposed intermediates and products formed for the PCET and protonation reactivity of the **[LNiOH]$^{n-}$** system. Note: The reductive/protonation or protonation of **[LNiOH]$^{n-}$** can occur at the hydroxide, but also at the N donors of the NNN ligand (like it has been shown by Heyduk and co-workers in the reactivity of a Ni bound by an SNS ligand[6]), or at the O atom of the ureanyl group (like it has been observed by Borovik and co-workers in the protonation reactivity of Fe^{IV}-oxo complex[7]).

4.4. [LNiOH]⁻ reactivity with TEMPOH

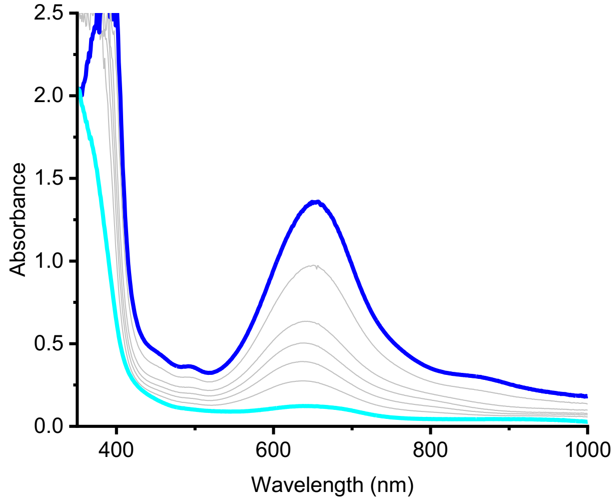

Figure S9. UV-vis spectra for the reaction of **[LNiOH]⁻** (0.25 mM, blue) with 20 equiv of TEMPOH under Ar at room temperature.

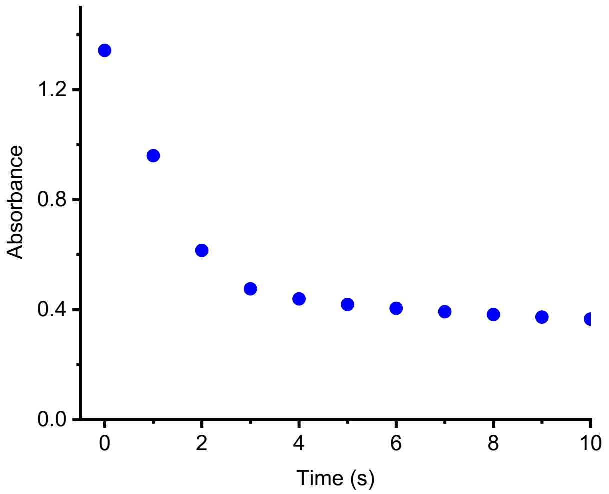

Figure S10. UV-vis time trace monitored at 655 nm for the reaction of **[LNiOH]⁻** (0.25 mM) with 20 equiv of TEMPOH under Ar at room temperature.

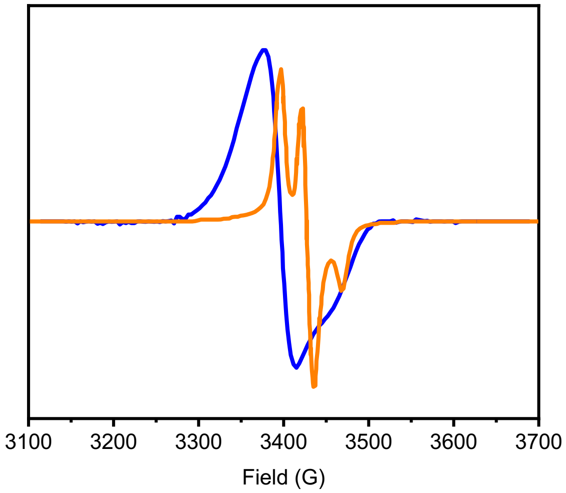

Figure S11. EPR spectra for the TEMPO radical generated in the reaction of **[LNiOH]**$^-$ (1 mM) with 20 equiv of TEMPOH under Ar at room temperature. Final spectrum in orange. 0.8 mM of TEMPO radical was produced, corresponding to an 80% yield of a $1H^+/1e^-$ process.

4.5. [LNiOH]⁻ reactivity with PhNHNHPh

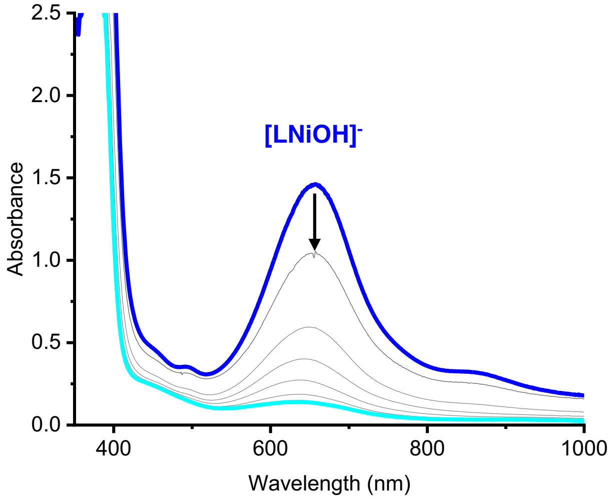

Figure S12. UV-vis spectra for the reaction of **[LNiOH]⁻** (0.25 mM, blue) with 100 equiv of PhNHNHPh under Ar at room temperature.

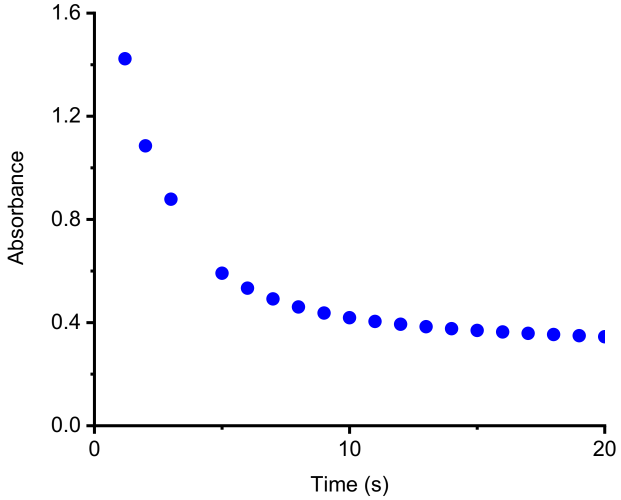

Figure S13. UV-vis time trace monitored at 655 nm for the reaction of **[LNiOH]⁻** (0.25 mM) with 100 equiv of PhNHNHPh under Ar at room temperature.

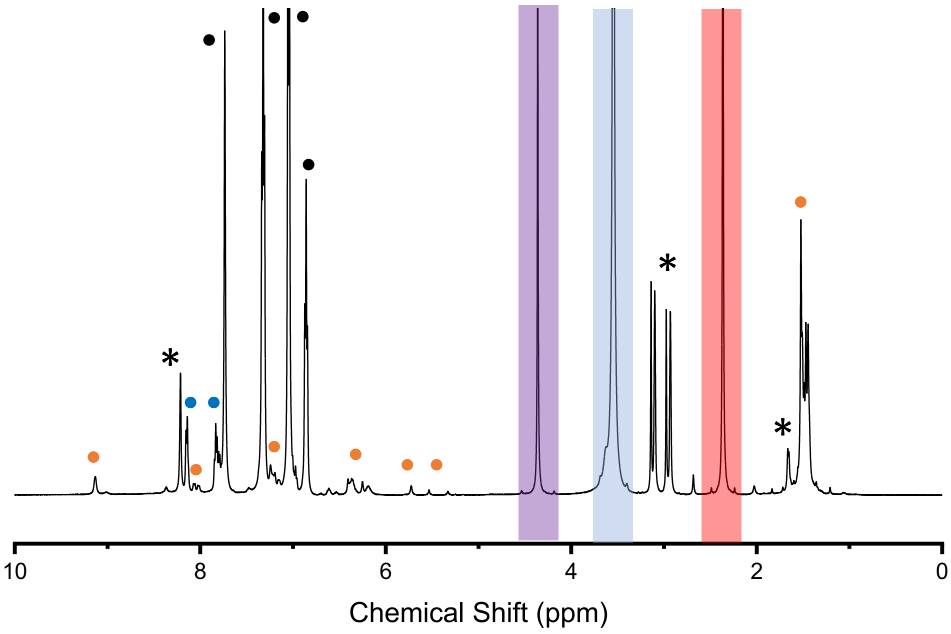

Figure S14. ^{1}H-NMR spectrum for the reaction between **[LNiOH]$^-$** and PhNHNHPh in DMF-*d*$_7$. Orange dots represent **[LNiOH(H)]$^-$**, blue dots represent PhN=NPh, black dots represent excess PhNHNHPh. Internal standard is highlighted in red, ferrocene highlighted in purple, NMe_4^+ cation highlighted in blue, and DMF and other trace impurities are denoted by *. PhN=NPh yield: ~97% based on $1H^+/1e^-$ process. **[LNiOH(H)]$^-$** could not be quantified due to the formation of multiple isomers (see Figure S8) that overlap with the PhNHNHPh and PhN=NPh peaks.

4.6. $[LNiOH]^{2-}$ protonation with $DMF \cdot CF_3SO_3H$

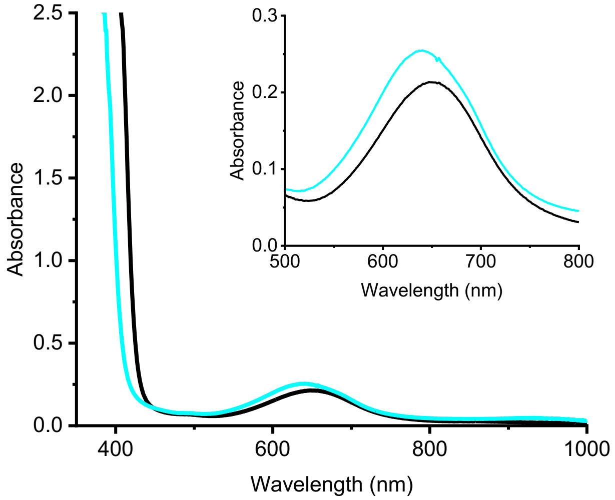

Figure S15. Addition of 1 equiv of $DMF \cdot CF_3SO_3H$ to **$[LNiOH]^{2-}$** (black). Inset is the magnified spectrum centered at 650 nm.

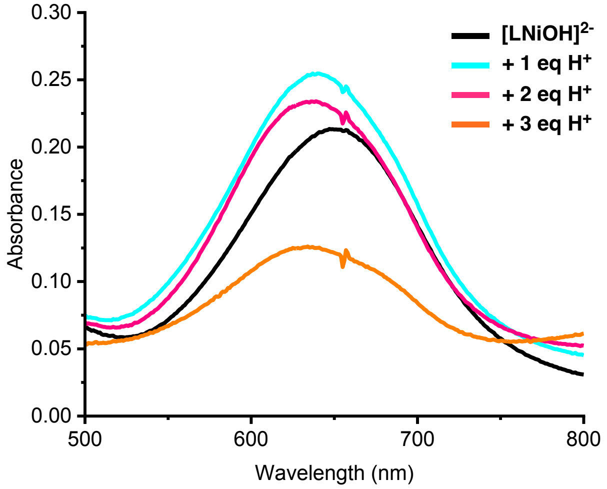

Figure S16. Addition of stoichiometric amounts of $DMF \cdot CF_3SO_3H$ to **$[LNiOH]^{2-}$** (from 1 to 3 equiv).

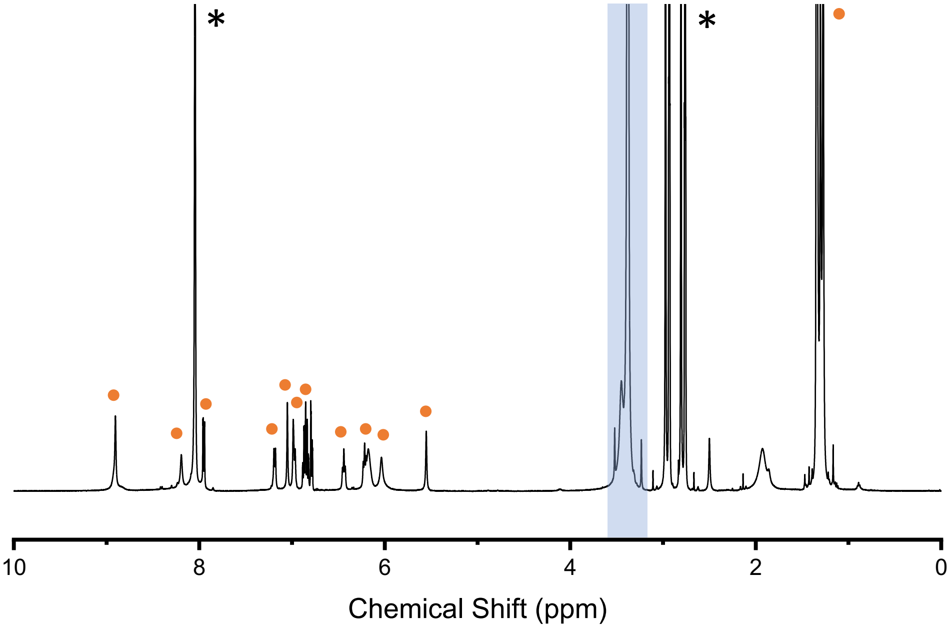

Figure S17. ^{1}H-NMR spectrum for the protonation of **$[LNiOH]^{2-}$** to **$[LNiOH(H)]^{-}$** with DMF·CF_3SO_3H at room temperature (1 equiv of acid was added). Peaks for **$[LNiOH(H)]^{-}$** are denoted with orange dots, NMe_4^+ cation is represented by the blue highlight, and DMF impurities are denoted by *. Note: the NMR spectrum suggests the formation of multiple isomers for the protonation of **$[LNiOH]^{2-}$** to **$[LNiOH(H)]^{-}$**. The integration of the peaks from 5.5 to 9 ppm was compared with the ^{t}Bu peaks, giving a ratio of 9 to 18 (expected ratio is 11 to 18).

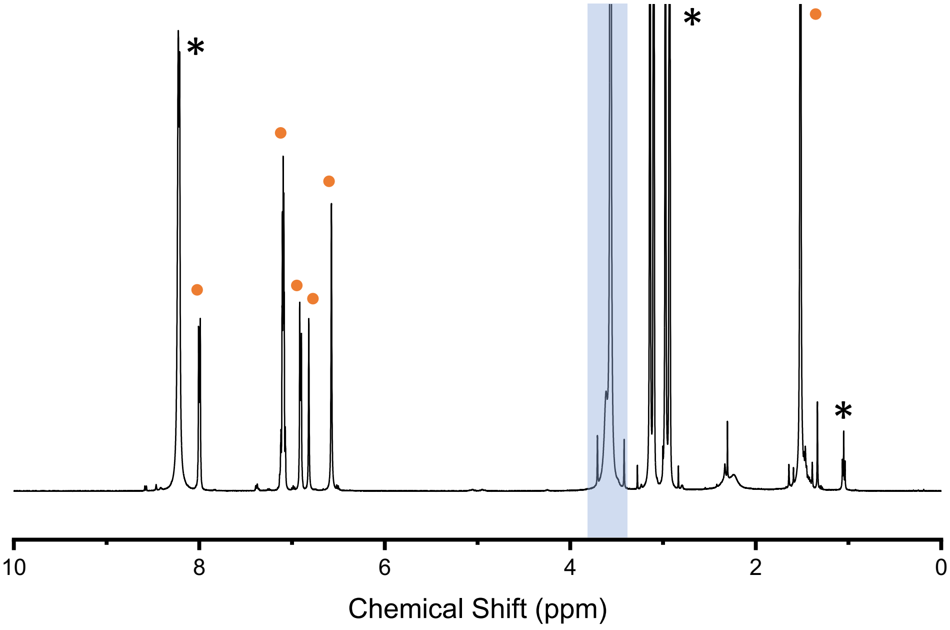

Figure S19. ^{1}H-NMR spectrum for the protonation of **$[LNiOH]^{2-}$** to **$[LNiOH(H)_2]$** (forms LH_3, see manuscript text) with 2 equivalents of DMF·CF_3SO_3H at room temperature. Peaks for LH_3 are denoted with orange dots, NMe_4^+ cation is represented by the blue highlight, and DMF impurities are denoted by *. Note: LH_3 yield: ~96%.

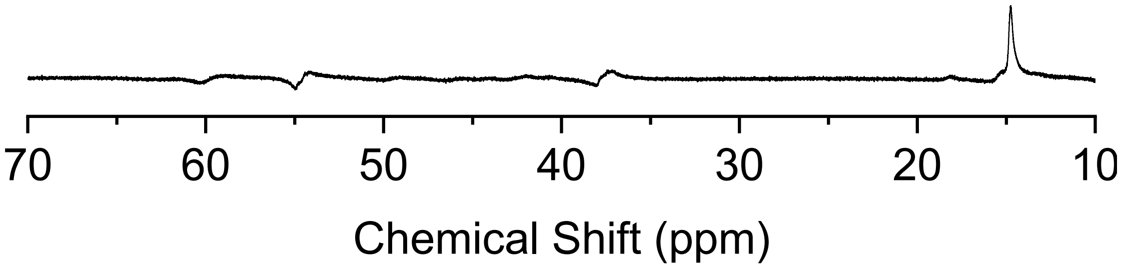

Figure S18. ^{1}H-NMR spectrum (paramagnetic mode) for the protonation of **$[LNiOH]^{2-}$** with subsequent addition of 3 equiv bipyridine. The peak at 15 ppm is consistent with the formation of $[Ni(bpy)_3]^{2+}$.[8,9] Note: The spectrum was recorded from 100 to -100 ppm but paramagnetic peaks only appeared between 10 and 70 ppm.

4.7. [LNiOH] reactivity with TEMPOH

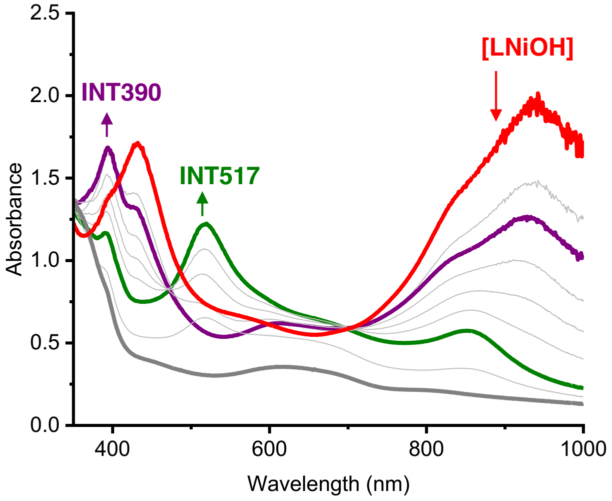

Figure S20. UV-vis spectra for the reaction of **[LNiOH]** (0.25 mM, red) with 20 equiv of TEMPOH under Ar at room temperature.

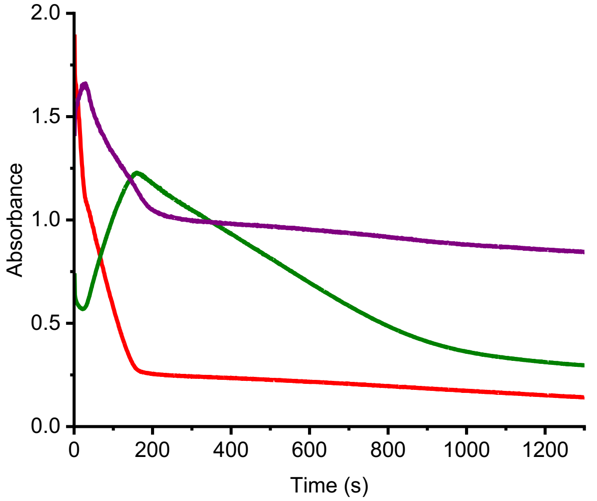

Figure S21. UV-vis time trace monitored at 390 nm (purple), 517 nm (green) and 950 nm (red) for the reaction of **[LNiOH]** (0.25 mM) with 20 equiv of TEMPOH under Ar at room temperature.

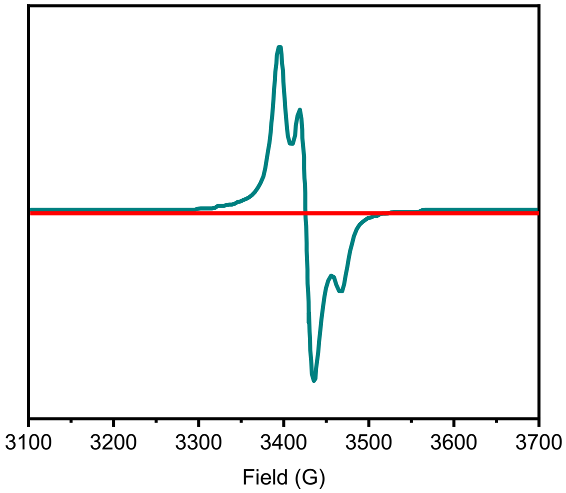

Figure S22. EPR spectra for the TEMPO radical generated in the reaction of **[LNiOH]** (1 mM) with 20 equiv of TEMPOH under Ar at room temperature. Initial **[LNiOH]** spectrum in red, final spectrum in turquoise. 2.0 mM of TEMPO radical was produced, corresponding to ~100% yield for a $2H^+/2e^-$ process.

4.8. [LNiOH] reactivity with PhNHNHPh

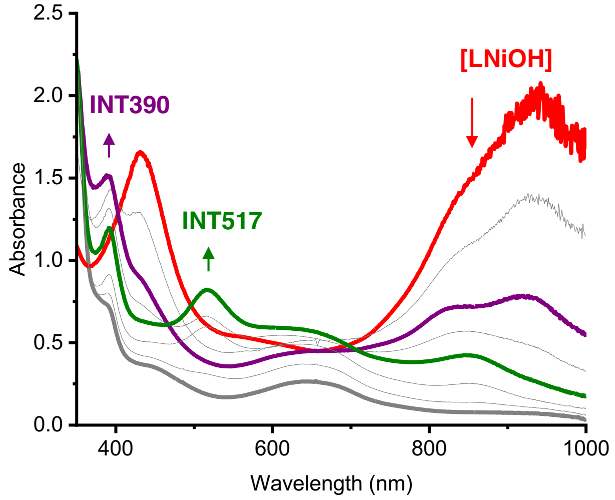

Figure S23. UV-vis spectra for the reaction of **[LNiOH]** (0.25 mM, red) with 100 equiv of PhNHNHPh under Ar at room temperature.

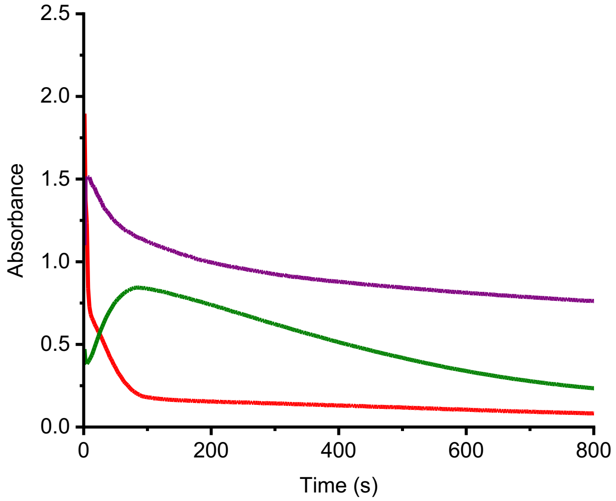

Figure S24. UV-vis time trace monitored at 390 nm (purple), 517 nm (green) and 950 nm (red) for the reaction of **[LNiOH]** (0.25 mM) with 100 equiv of PhNHNHPh under Ar at room temperature.

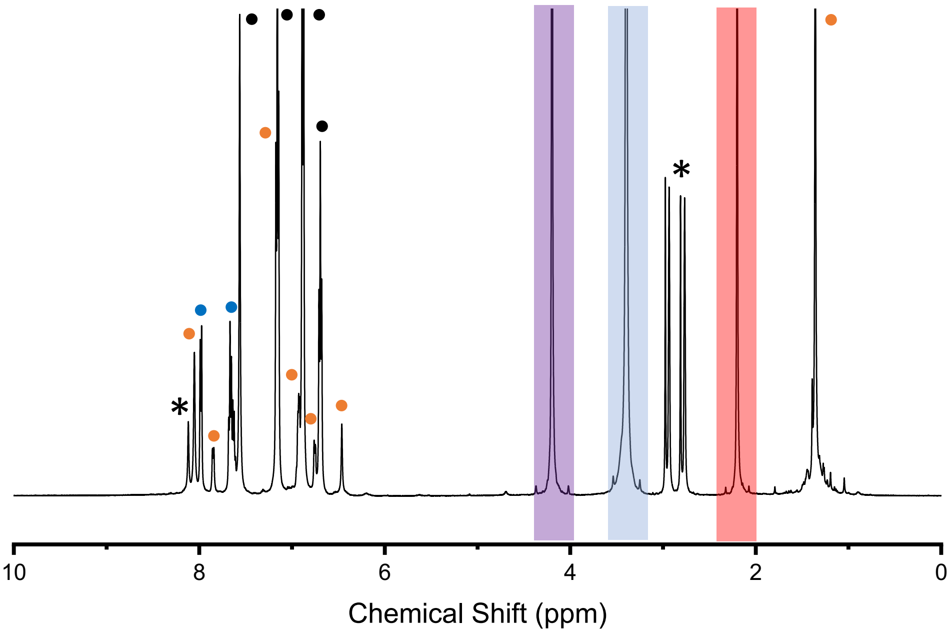

Figure S25. ^{1}H-NMR spectrum for the reaction between **[LNiOH]** and PhNHNHPh in DMF-d_7. Orange dots represent **[LNiOH(H)]** (which disproportionates into **INT517** and a reduced nickel complex, ultimately forming ligand, LH_3; see Figure S8), blue dots represent PhN=NPh, black dots represent excess PhNHNHPh. Internal standard is highlighted in red, ferrocene highlighted in purple, NMe_4^+ cation highlighted in blue, and DMF and other trace impurities are denoted by *. Note: PhN=NPh yield: 72% based on a $2H^+/2e^-$ process. LH_3 yield: 75% based on the proposed stoichiometry.

4.9. [LNiOH] reactivity with 4-MeO-2,6-DTBP

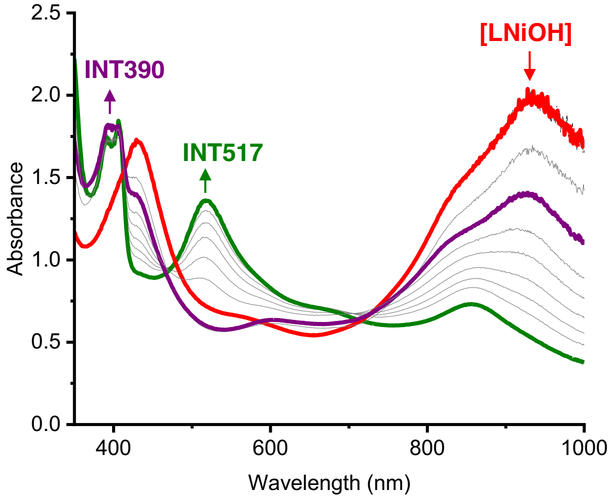

Figure S26. UV-vis spectra for the reaction of **[LNiOH]** (0.25 mM, red) with 100 equivalents of 4-MeO-2,6-DTBP under Ar at room temperature.

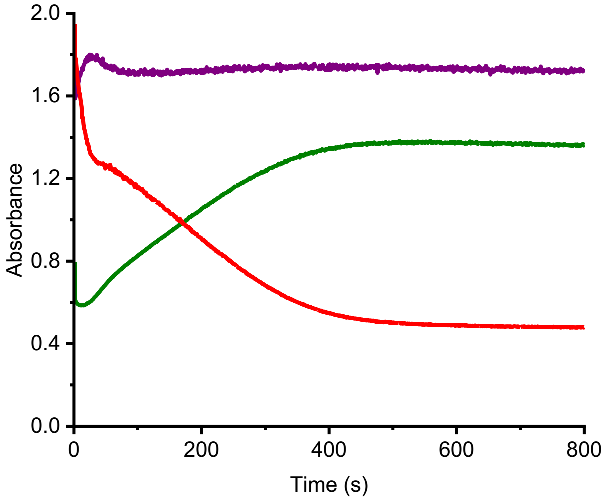

Figure S27. UV-vis time trace monitored at 390 nm (purple), 517 nm (green) and 950 nm (red) for the reaction of **[LNiOH]** (0.25 mM) with 100 equivalents of 4-MeO-2,6-DTBP under Ar at room temperature.

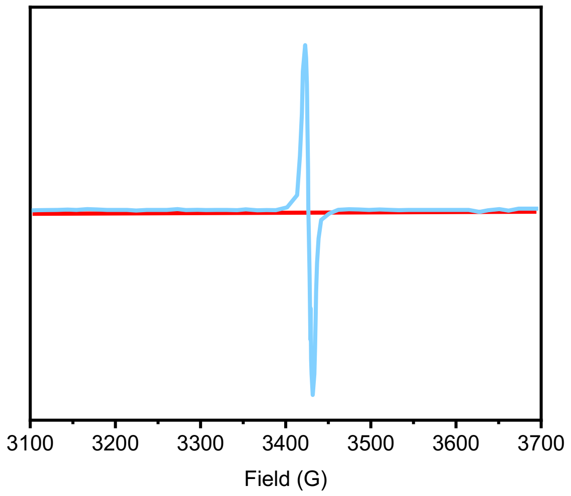

Figure S28. EPR spectrum for the phenoxyl radical generated in the reaction of **[LNiOH]** (1 mM) with 20 equivalents of 4-MeO-2,6-DTBP under Ar at room temperature. Initial **[LNiOH]** spectrum in red, final spectrum in light blue. 0.78 mM of the phenoxyl radical was produced, corresponding to a 78% yield of a $1H^{+}/1e^{-}$ process.

4.10. $[LNiOH]^-$ protonation with $DMF{\cdot}CF_3SO_3H$

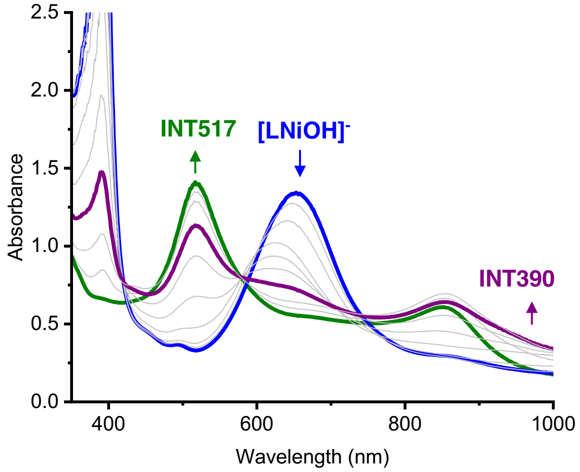

Figure S29. UV-vis spectra for the titration of **$[LNiOH]^-$** (blue) with 1 equiv (purple) and 2 equiv of $DMF{\cdot}CF_3SO_3H$ (green, **INT517**) under Ar at room temperature.

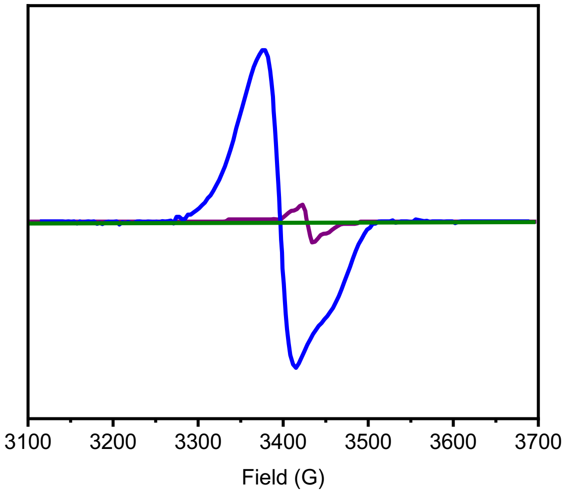

Figure S30. EPR spectra of the titration of **$[LNiOH]^-$** (blue) with 1 equiv (purple) and 2 equiv of $DMF{\cdot}CF_3SO_3H$ (green, **INT517**) at room temperature. Note the purple spectrum (5% sample that differs from the starting complex. We hypothesize that this signal belongs to **INT390**.

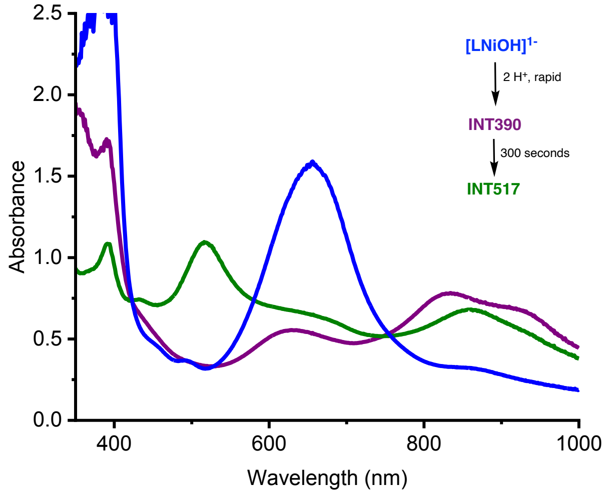

Figure S31. UV-vis spectra for the addition of the 2 equiv of DMF·CF_3SO_3H to **[LNiOH]**$^-$ at room temperature. **INT390** formed immediately, followed by the gradual formation of **INT517**.

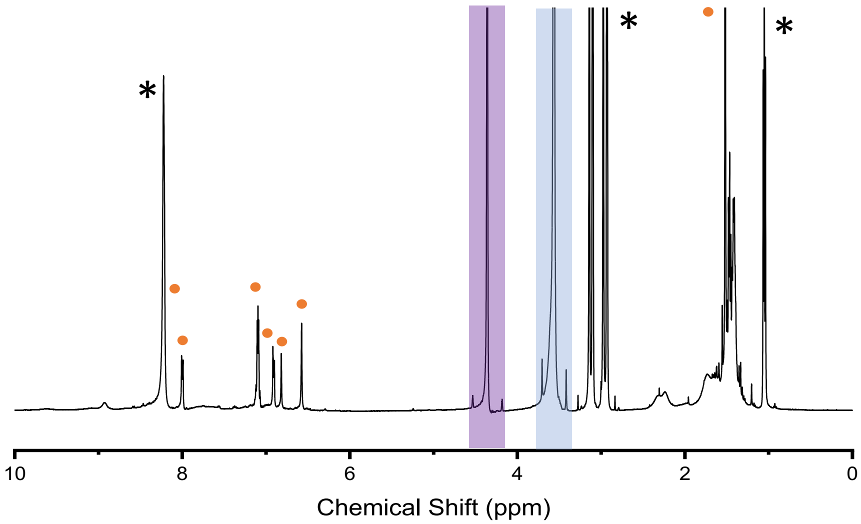

Figure S32. ^{1}H-NMR spectrum for the protonation of **[LNiOH]**$^-$ with 1 equiv of DMF·CF_3SO_3H at room temperature. Peaks for ligand are denoted with orange dots, NMe_4^+ cation is represented by the blue highlight, ferrocene represented by purple highlight, and DMF and other trace impurities are denoted by *. Note: 0.25 equiv of LH_3 are formed (~50% yield based on the proposed stoichiometry). The broad region from 6 to 9 ppm is consistent with the formation of the diamagnetic **INT517**.

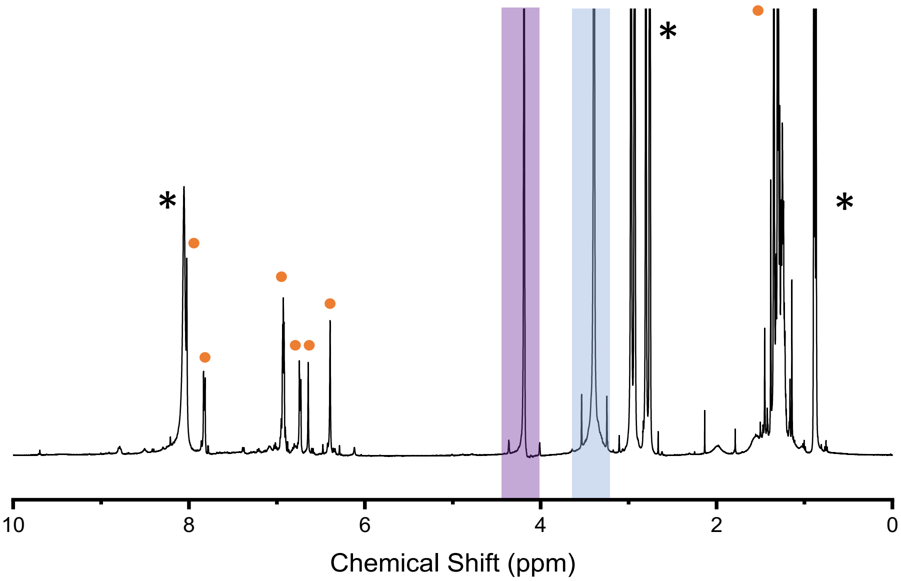

Figure S33. ^{1}H-NMR spectrum for the protonation of **[LNiOH]**$^-$ with 2 equiv of DMF·CF_3SO_3H at room temperature. Peaks for ligand are denoted with orange dots, NMe_4^+ cation is represented by the blue highlight, ferrocene represented by purple highlight, and DMF and other trace impurities are denoted by *. Note: 0.35 equiv of LH_3 are formed (~70% yield based on the proposed stoichiometry). The broad region from 6 to 9 ppm is consistent with the formation of the diamagnetic **INT517**.

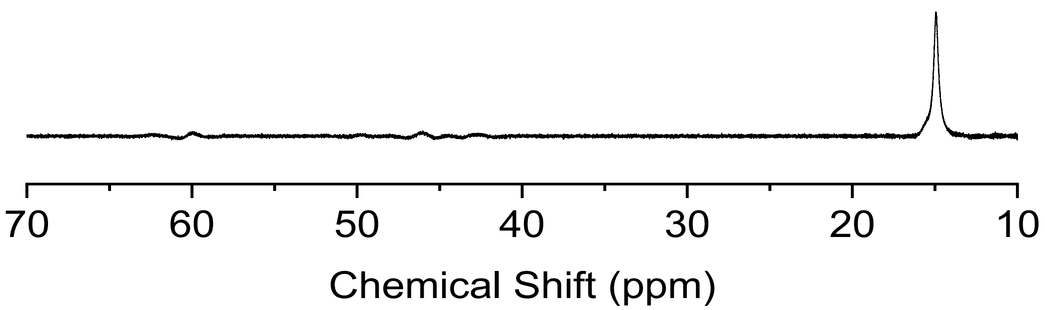

Figure S34. ^{1}H-NMR spectrum (paramagnetic mode) for the protonation of **[LNiOH]$^{-}$** with subsequent addition of 3 equiv of bipyridine. The peak at 15 ppm is consistent with the formation of $[Ni(bpy)_3]^{2+}$.[8,9] The spectrum was recorded from 100 to -100 ppm but paramagnetic peaks only appeared between 10 and 70 ppm.

4.11. INT517 reactivity with PCET reagents

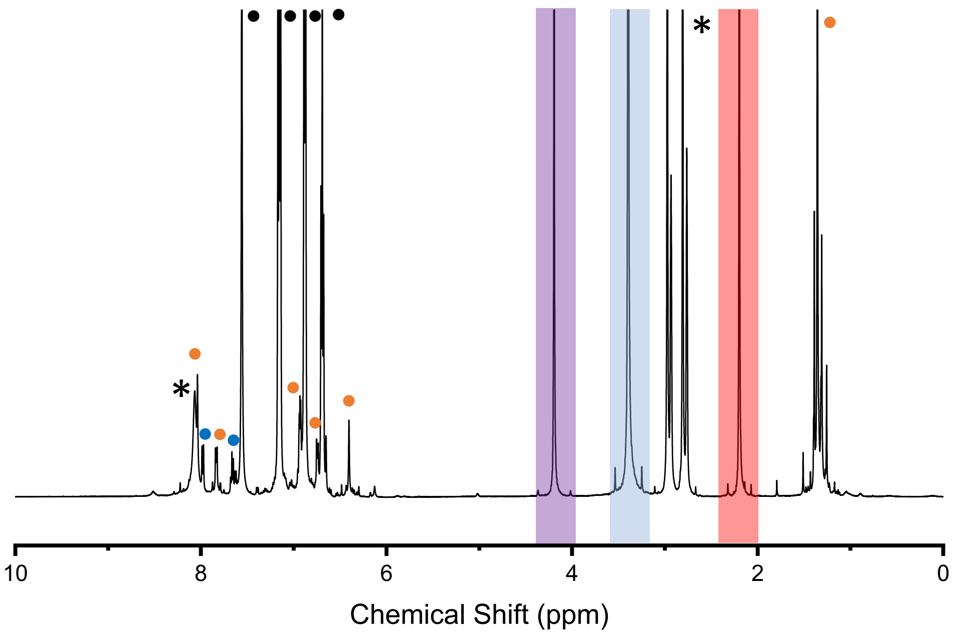

Figure S35. ^{1}H-NMR spectrum for the reaction between **INT517** and PhNHNHPh in DMF-d_7. Orange dots represent ligand, LH_3, blue dots represent PhN=NPh, black dots represent excess PhNHNHPh. Internal standard is highlighted in red, ferrocene highlighted in purple, NMe_4^+ cation highlighted in blue, and DMF and other trace impurities are denoted by *. Note: PhN=NPh yield: 90% based on $1H^+/1e^-$ process. LH_3 yield: ~100% based on the proposed stoichiometry.

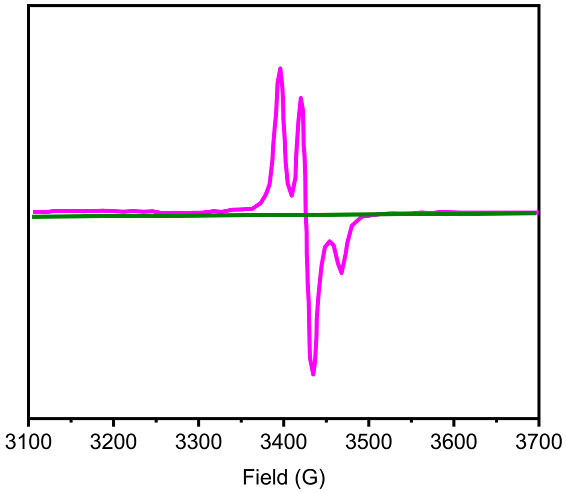

Figure S36. EPR spectra for the TEMPO radical generated in the reaction of **INT517** (1 mM) with 20 equivalents of TEMPOH under Ar at room temperature. Initial **INT517** spectrum in green, final spectrum in pink. 0.8 mM of TEMPO radical was produced, corresponding to ~80% yield of a $1H^{+}/1e^{-}$ process.

4.12. [LCuOH]⁻ reactivity with TEMPOH

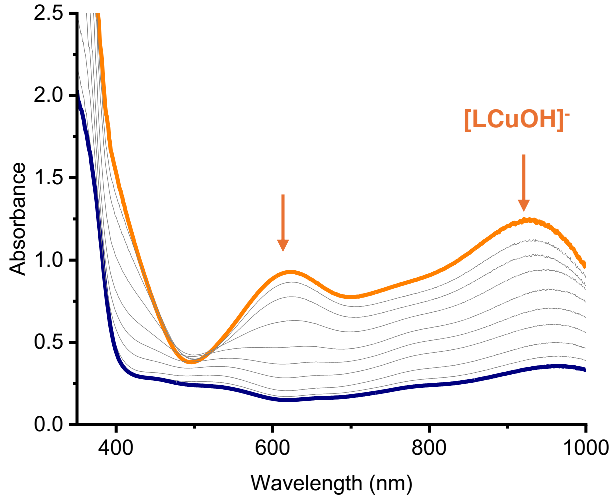

Figure S37. UV-vis spectra for the reaction of **[LCuOH]⁻** (0.25 mM, orange) with 20 equiv of TEMPOH under Ar at room temperature.

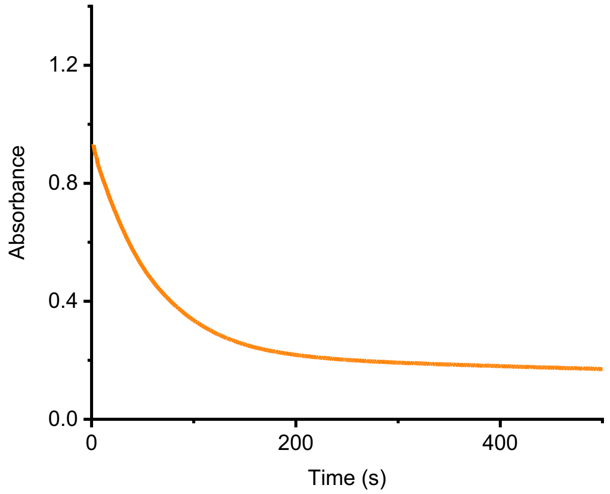

Figure S38. UV-vis time trace monitored at 625 nm for the reaction of **[LCuOH]⁻** (0.25 mM) with 20 equiv of TEMPOH under Ar at room temperature.

4.13. [LCuOH] reactivity with TEMPOH

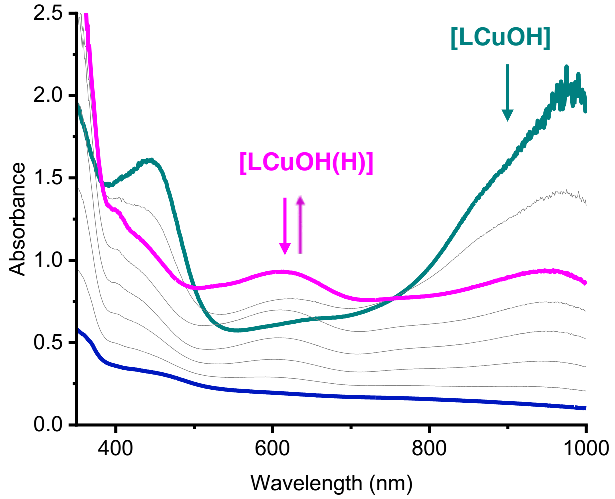

Figure S39. UV-vis spectra for the reaction of **[LCuOH]** (0.25 mM, turquoise) with 20 equiv of TEMPOH under Ar at room temperature.

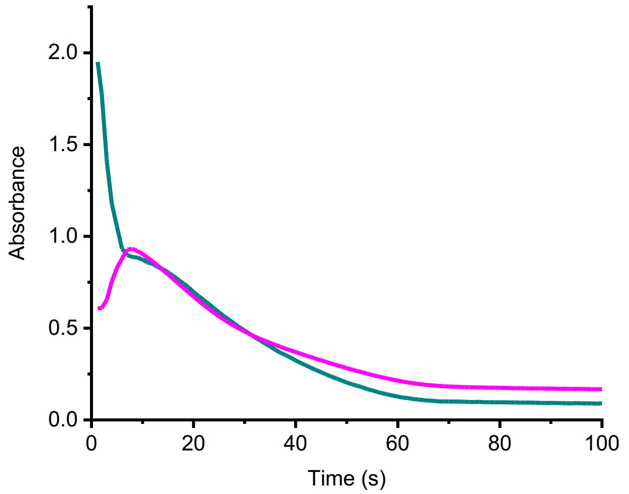

Figure S40. UV-vis time trace monitored at 965 nm (turquoise) and 600 nm (magenta) for the reaction of **[LCuOH]** (0.25 mM) with 20 equiv of TEMPOH under Ar at room temperature.

5. PCET reactivity: Thermochemistry

5.1. Bordwell square schemes for $[LMOH]^{n-}$ systems

The Bordwell equation can be used to determine the thermodynamic driving force for the reductive protonation of **$[LMOH]^{n-}$**, described by the bond dissociation free energy (BDFE). The scheme below summarizes the thermodynamic values measured (such as $E_{1/2}$) and estimated (such as $BDFE^{(3)}$ and $pK_a^{(3)}$) for the NiOH complexes. The analogous CuOH square scheme is also shown for comparison. Note the C_G value for DMF is 67.6 kcal/mol.[10]

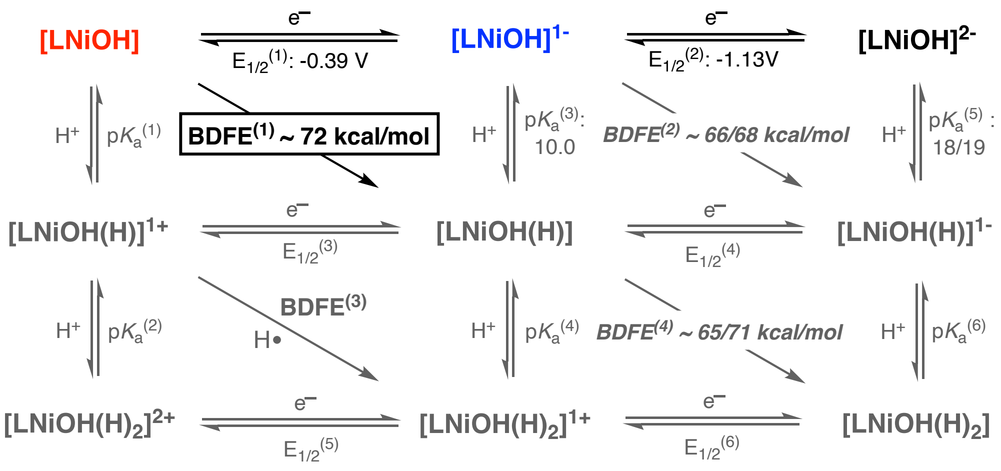

$$BDFE = 1.37pK_a + 23.06E_{1/2} + C_G$$

Figure S41. BDFE, pK_a, and $E_{1/2}$ values for the NiOH complexes determined in this article.

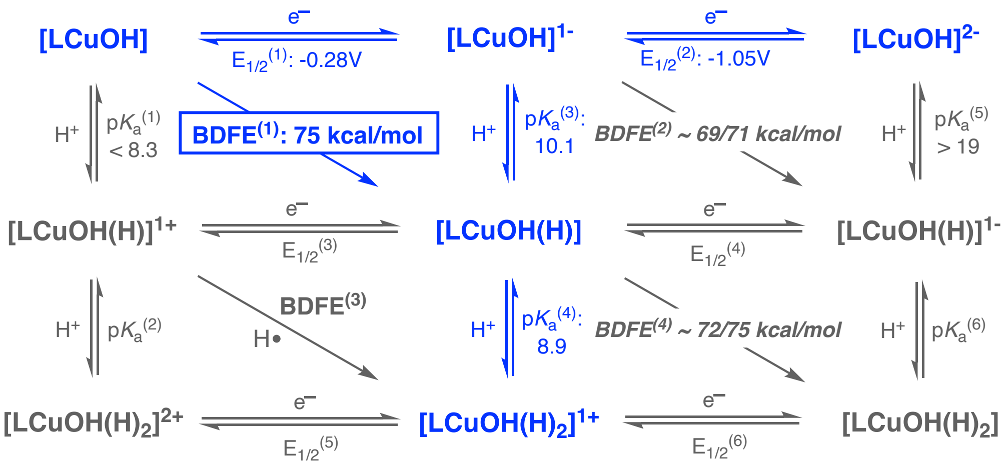

Figure S42. BDFE, pK_a, and $E_{1/2}$ values for the CuOH complexes

5.2. Determination of BDFE for the reductive protonation of [LNiOH]

5.2.1. Estimation of the pK_a for the [LNiOH]$^-$/[LNiOH(H)] couple

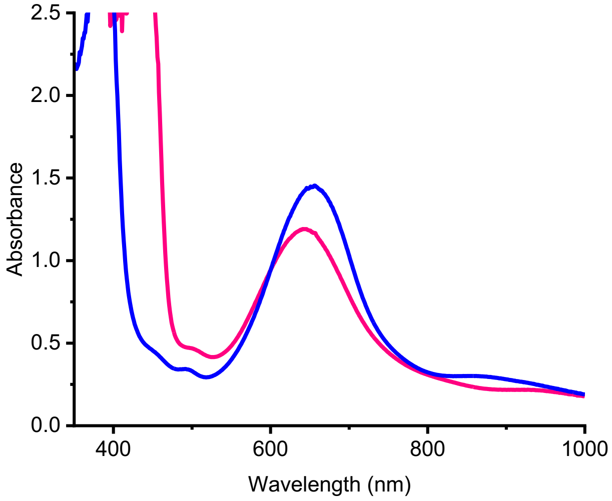

Figure S43. UV-vis spectra for the protonation of **[LNiOH]$^-$** (blue) with 10 equivalents of 4-NO_2-PhOH.

The protonation of **[LNiOH]$^-$** was found to be irreversible so the equilibrium of the protonation can only be estimated. However, if we assumed a reversible process, the equilibrium constant would be calculated as follows:

$$\textbf{[LNiOH]}^- + \text{4-NO}_2\text{-PhOH} \rightleftharpoons \textbf{[LNiOH(H)]} + \text{4-NO}_2\text{-PhO}^-$$

$$K_{eq} = \frac{x^2}{(0.25 * 10^{-3} - x)(10 * 0.25 * 10^{-3} - x)}$$

The change in absorbance at 655 nm is 0.27, corresponding to a change in concentration of

$x = 4.82 * 10^{-5}$ M

$K_{eq} = 0.0045$

$-\log K_{eq} = 2.3$

pK_a **[LNiOH(H)]** = 10.0

5.2.2. Calculation of the BDFE for the reductive protonation of [LNiOH]

$$\mathrm{BDFE} = 1.37\mathrm{p}K_a + 23.06\mathrm{E}_{1/2} + 67.6 \text{ in DMF}$$

$$\mathrm{BDFE} = 1.37(10) + 23.06(-0.39) + 67.6$$

$$\mathrm{BDFE} \sim 72.3 \text{ kcal/mol}$$

5.2.3. [LNiOH] reactivity with 2,4,6-TTBP and BDFE estimation

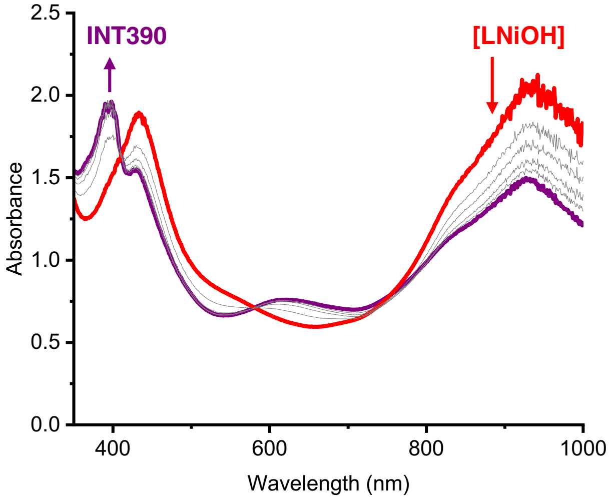

Figure S44. UV-vis spectra for the reaction of **[LNiOH]** (0.25 mM, red) with 100 equiv of 2,4,6-TTBP under Ar at room temperature. The final spectrum was taken after equilibrium was achieved.

The reductive protonation of **[LNiOH]** is an irreversible process. However, we can approximate the BDFE of this process, if we consider it a reversible equilibrium, as follows:

[LNiOH] + 2,4,6-TTBP $\rightleftharpoons$ **[LNiOH(H)]** + 2,4,6-TTBP•

$$K_{eq} = \frac{x^2}{(0.25 * 10^{-3} - x)(100 * 0.25 * 10^{-3} - x)}$$

The change in absorbance at 950 nm is 0.66, corresponding to a change in concentration of

$x = 8.3 * 10^{-5}$ M

$K_{eq} = 0.00166$

$\Delta G^{o} = -RT\ln K_{eq} = 3.8$

BDFE **[LNiOH]** = 71.3 kcal/mol

5.3. Determination of the BDFE for the reductive protonation of $[LNiOH]^-$

5.3.1. Estimation of the pK_a for the protonation of $[LNiOH]^{2-}$

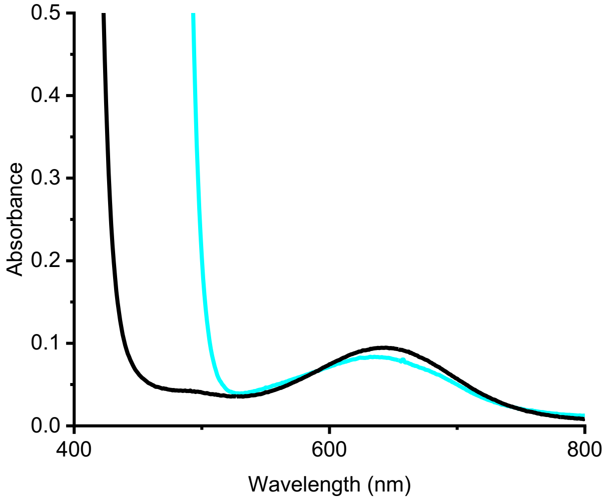

Figure S45. UV-vis spectra for the protonation of $\mathbf{[LNiOH]^{2-}}$ (black) with 1 equiv of 4-NO_2-2,6-DTBP.

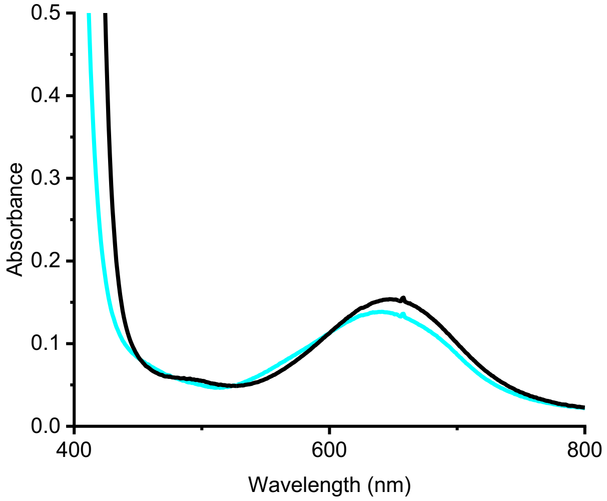

Figure S46. UV-vis spectrum for the protonation of $\mathbf{[LNiOH]^{2-}}$ (black) with 10 equiv of 2,6-DTBP.

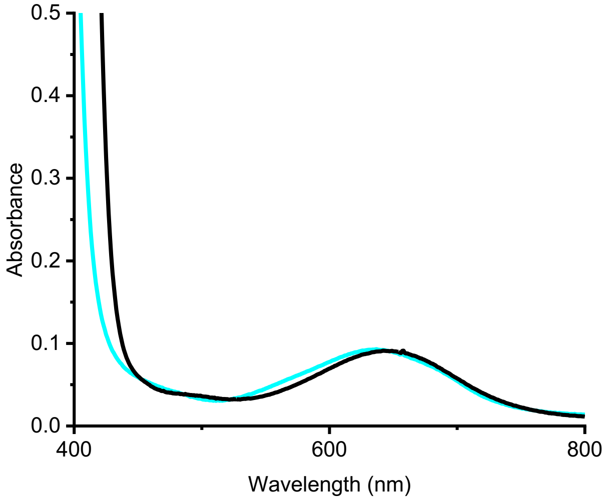

Figure S47. UV-vis spectrum for the protonation of **[LNiOH]$^{2-}$** (black) with 10 equiv of 4-MeO-2,6-DTBP.

5.3.2. Estimation of the BDFE for the reductive protonation of **[LNiOH]$^-$**

Since **[LNiOH]$^{2-}$** is partially protonated by addition of excess amounts of 2,6-DTBP and 4-MeO-2,6-DTBP, the estimated pK_a for the protonation of **[LNiOH]$^{2-}$** to **[LNiOH(H)]$^-$** is 18-19.

$$\text{BDFE} = 1.37\text{p}K_a + 23.06E_{1/2} + 67.6 \text{ in DMF}$$

$$\text{BDFE} = 1.37(18) + 23.06(-1.13) + 67.6$$

$$\text{BDFE} = 66.2 \text{ kcal/mol}$$

$$\text{BDFE} = 1.37(19) + 23.06(-1.13) + 67.6$$

$$\text{BDFE} = 67.6 \text{ kcal/mol}$$

Therefore, the BDFE for the reductive protonation of **[LNiOH]$^-$** is estimated to be between 66 and 68 kcal/mol.

6. PCET reactivity: Kinetics

In a typical reaction, 2.7 mL of a DMF solution of **[LMOH]$^{n-}$** (0.25 mM) were transferred to a 10 mm path quartz cell with a stir bar and a rubber septum. Differing amounts of a solution of $FcPF_6$ (0.25 mM or 0.5 mM) were injected into the complex solution to generate the corresponding “high-valent” species **[LMOH]$^{-}$** and **[LMOH].** DMF solutions of substrate (5 mM for TEMPOH) were stored in 500 µL gastight syringes and injected into the quartz cell correspondingly. All reactions were run under Ar flow and at -40 °C, with the exception of **[LCuOH]$^{-}$,** which was performed at room temperature. The decays of the **[LMOH]$^{n-}$** bands were monitored by UV-vis, which were fitted to the following exponential function:

$$\boldsymbol{Abs_t = Abs_\infty - Abs_0 \cdot e^{(-kobs\cdot t)}}$$

Plotting the k_{obs} values obtained at different substrate concentrations allows for the determination of k_2, the second order rate constant for each reaction (except **[LNiOH]**).

The k_{obs} value for the PCET reaction between **[LNiOH]** and TEMPOH was calculated using the initial rates method, with the assumption that d[Ni]/dt ~ Δ[Ni]/Δt. We used the initial rates method because the reductive protonation of **[LNiOH]** produces INT390 and INT517, and the UV-vis spectra of these intermediates overlap with **[LNiOH]**. The initial slope of the decay of **[LNiOH]** was recorded for each concentration of TEMPOH used, and this was subsequently converted to a change in concentration, and finally k_{obs}.

All kinetics experiments performed for Eyring analysis were conducted with **[LMOH]$^{n-}$** (0.25 mM) and TEMPOH (5 mM). The activation parameters $\Delta H^{\ddagger}$, and $\Delta S^{\ddagger}$ were determined by the Eyring equation:[11]

$$ln\frac{k_2}{T} = \frac{-\Delta H^{\ddagger}}{R}\frac{1}{T} + ln\frac{k_B}{h} + \frac{\Delta S^{\ddagger}}{R}$$

$\Delta G^{\ddagger}$ was calculated by $\Delta G^{\ddagger} = \Delta H^{\ddagger} - T\Delta S^{\ddagger}$, and T = 233 K was used for all calculations.

6.1. Kinetics for the reaction of $[LNiOH]^-$ with TEMPOH

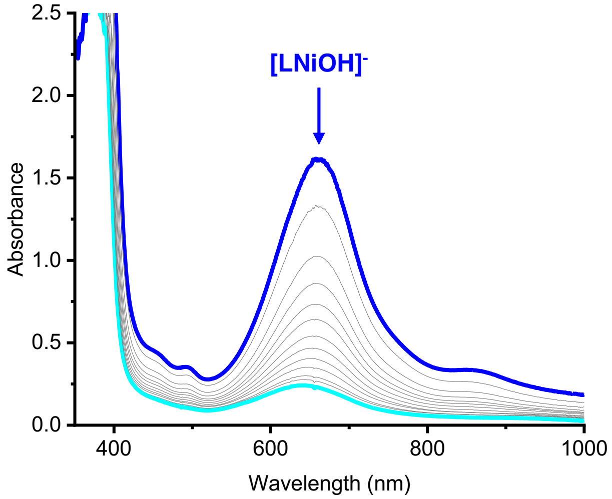

Figure S48. UV-vis spectra of the reaction between **$[LNiOH]^-$** (blue) and 20 equiv of TEMPOH under Ar at -40 °C

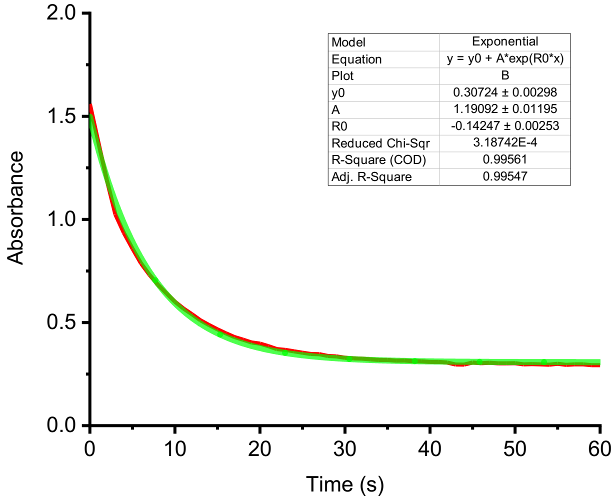

Figure S49. UV-vis time trace at 655 nm for the reaction of **$[LNiOH]^-$** and 20 equivalents of TEMPOH under Ar at -40 °C. Exponential fitting shown in green.

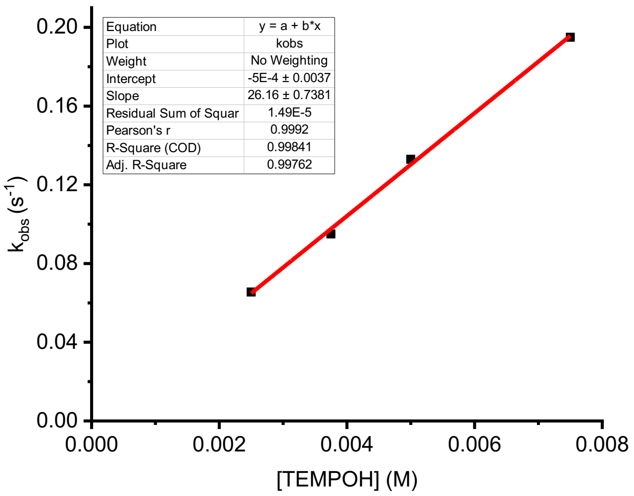

Figure S50. Second order rate constant for the reaction between **[LNiOH]$^-$** and TEMPOH at -40 ^{o}C (k_2 = 26.2 $M^{-1}s^{-1}$). Note: Self decay rate constant for **[LNiOH]$^-$** is 3.1*10^{-5} s^{-1}.

6.2. Kinetics for the reaction of $[LNiOH]^-$ with TEMPOD

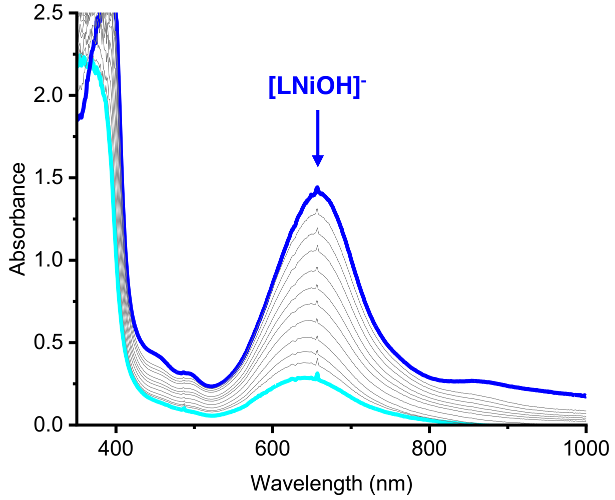

Figure S51. UV-vis spectra of the reaction between **$[LNiOH]^-$** (blue) and 20 equivalents of TEMPOD under Ar at -40 °C

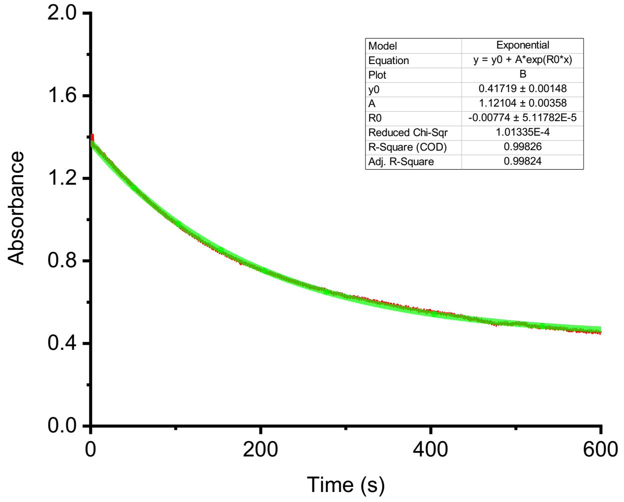

Figure S52. UV-vis time trace at 655 nm for the reaction of **$[LNiOH]^-$** and 20 equivalents of TEMPOD under Ar at -40 °C. Exponential fitting shown in green.

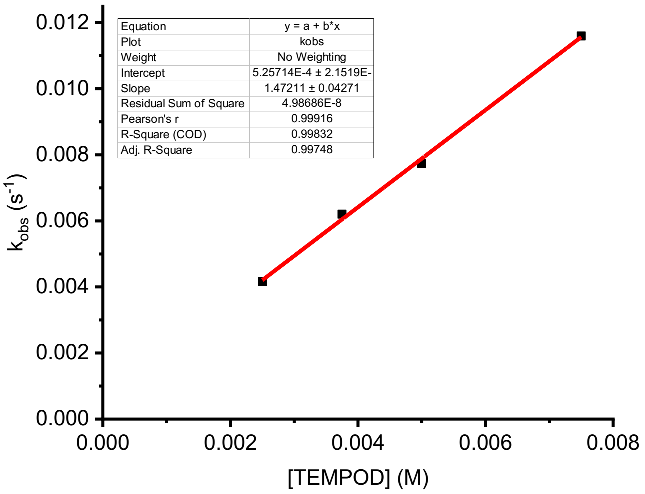

Figure S53. Second order rate constant for the reaction between **[LNiOH]**$^-$ and TEMPOD at -40 ^{o}C (k_2 = 1.47 $M^{-1}s^{-1}$)

6.3. Kinetics for the reaction of [LNiOH] with TEMPOH

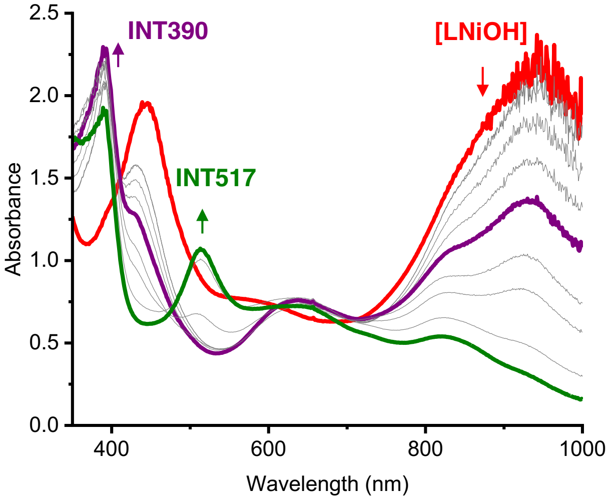

Figure S54. UV-vis spectra of the reaction between **[LNiOH]** (red) and 20 equiv of TEMPOH under Ar at -40 °C. Note that the spectrum only depicts the reaction up to the formation of INT517.

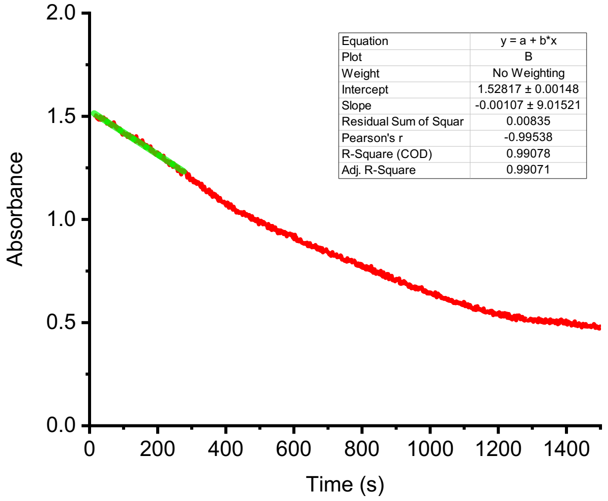

Figure S55. UV-vis time trace at 850 nm for the reaction of **[LNiOH]** and 20 equiv of TEMPOH under Ar at -40 °C. Initial rate (linear) fitting shown in green.

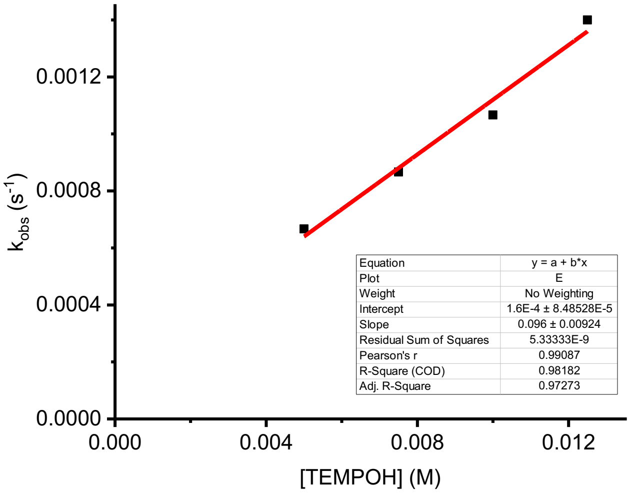

Figure S56. Second order rate constant for the reaction between **[LNiOH]** and TEMPOH at -40 ^{o}C (k_2 = 0.1 $M^{-1}s^{-1}$). Note: Self decay rate constant for **[LNiOH]** is 2.2*10^{-4} s^{-1}.

6.4. Kinetics for the reaction of [LNiOH] with TEMPOD

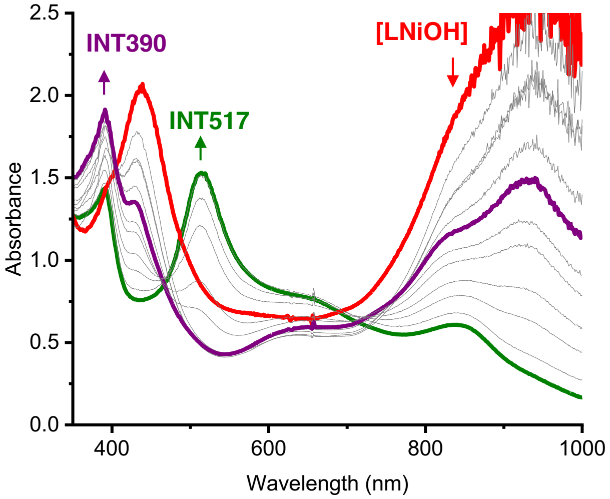

Figure S57. UV-vis spectra of the reaction between **[LNiOH]** (red) and 20 equiv of TEMPOD under Ar at -40 °C. Note that the spectrum only depicts the reaction up to the formation of INT517.

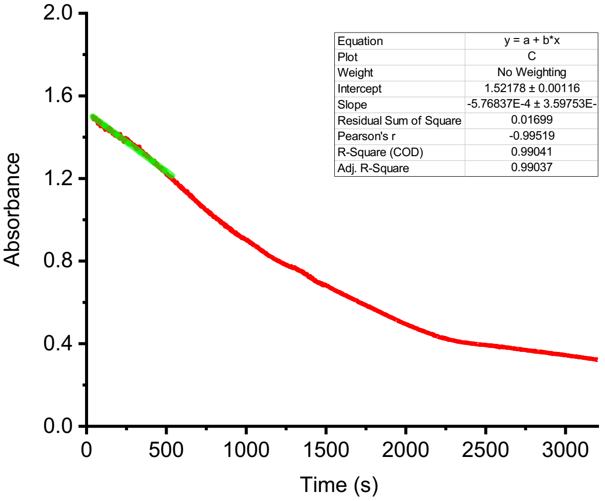

Figure S58. UV-vis time trace at 850 nm for the reaction of **[LNiOH]** and 20 equivalents of TEMPOD under Ar at -40 °C. Initial rate (linear) fitting shown in green.

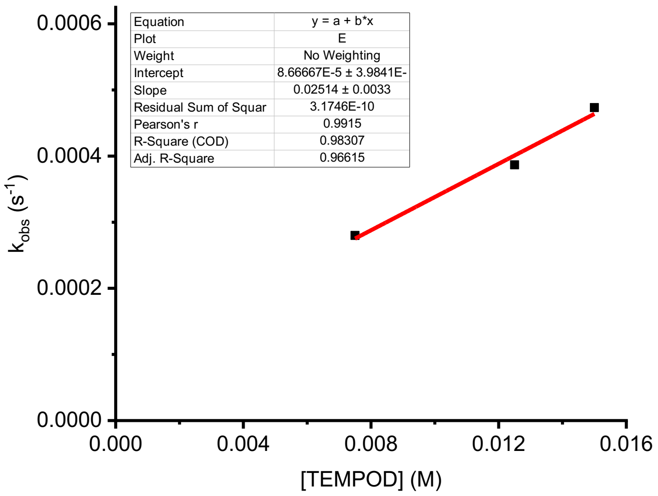

Figure S59. Second order rate constant for the reaction between **[LNiOH]** and TEMPOH at -40 °C (k_2 = 0.025 $M^{-1}s^{-1}$)

6.5. Kinetics for the reaction of $[LCuOH]^-$ with TEMPOH

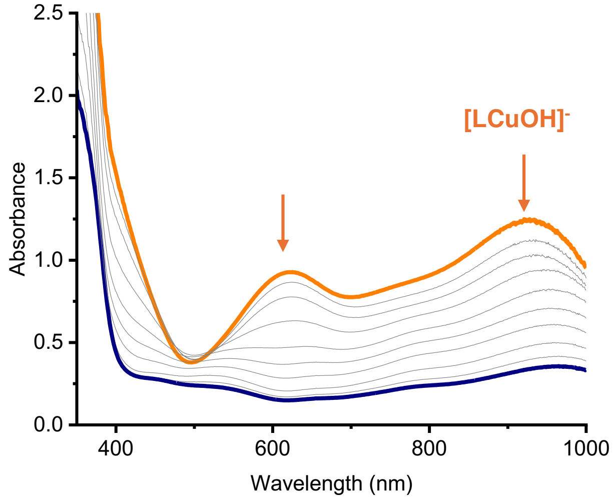

Figure S60. UV-vis spectra of the reaction between **$[LCuOH]^-$** (orange) and 20 equiv of TEMPOH under Ar at room temperature.

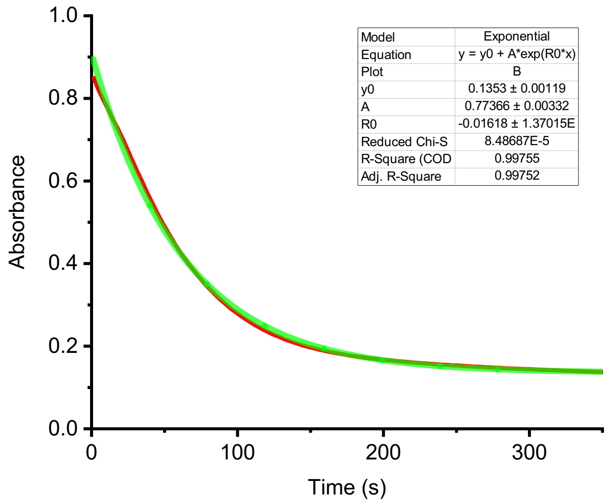

Figure S61. UV-vis time trace at 950 nm for the reaction of **$[LCuOH]^-$** and 20 equiv of TEMPOH under Ar at room temperature. Exponential fitting shown in green.

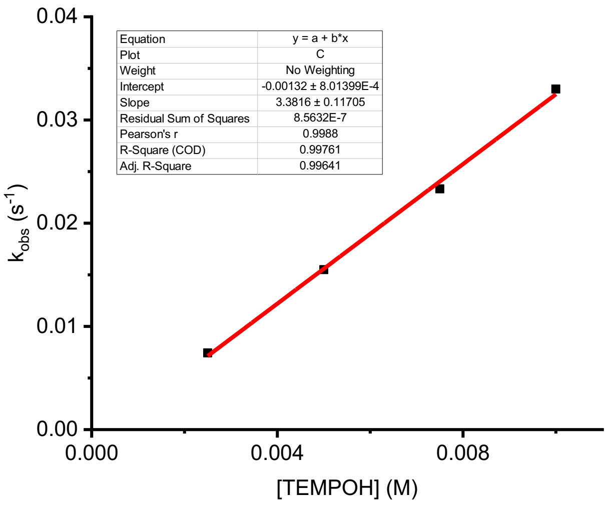

Figure S62. Second order rate constant for the reaction between **[LCuOH]⁻** and TEMPOH at room temperature (k_2 = 3.4 $M^{-1}s^{-1}$)

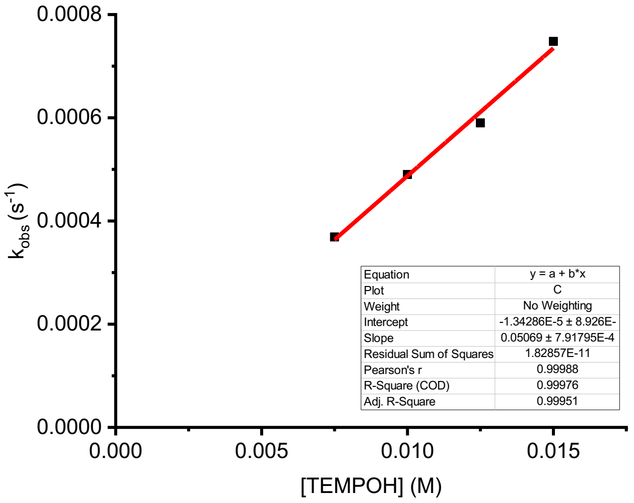

Figure S63. Second order rate constant for the reaction between **[LCuOH]⁻** and TEMPOH at -40 °C (k_2 = 0.05 $M^{-1}s^{-1}$)

6.6. Kinetics for the reaction of $[LCuOH]^-$ with TEMPOD

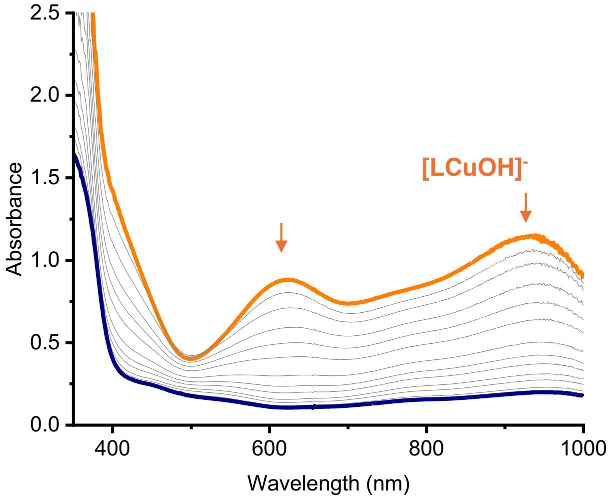

Figure S64. UV-vis spectra of the reaction between **$[LCuOH]^-$** (orange) and 20 equivalents of TEMPOD under Ar at room temperature.

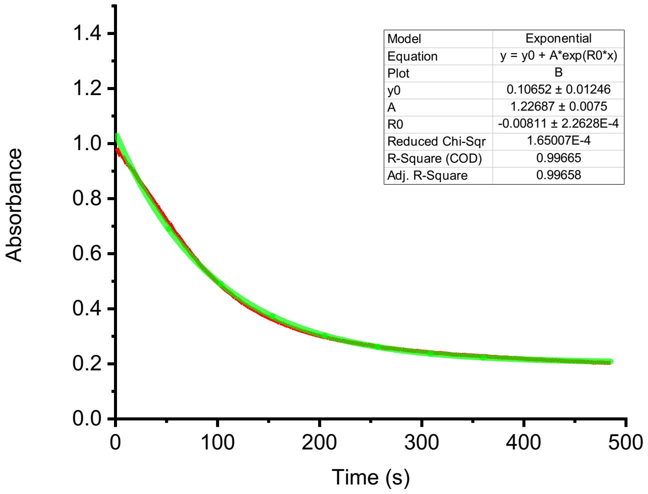

Figure S65. UV-vis time trace at 950 nm for the reaction of **$[LCuOH]^-$** and 20 equivalents of TEMPOD under Ar at room temperature. Exponential fitting shown in green.

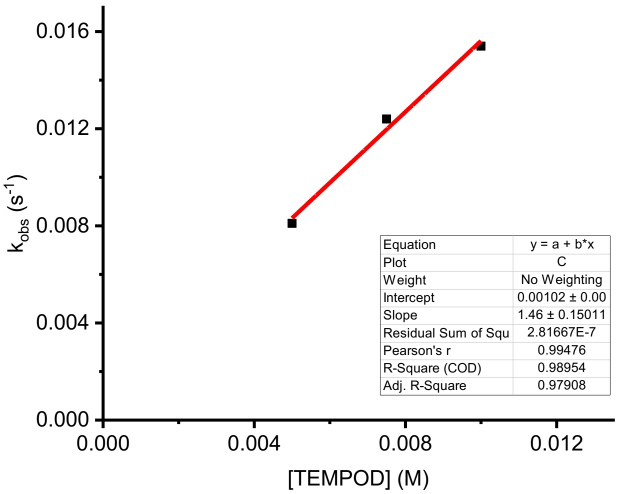

Figure S66. Second order rate constant for the reaction between **[LCuOH]$^-$** and TEMPOD at room temperature (k_2 = 1.46 $M^{-1}s^{-1}$)

6.7. Kinetics for the reaction of [LCuOH] with TEMPOH

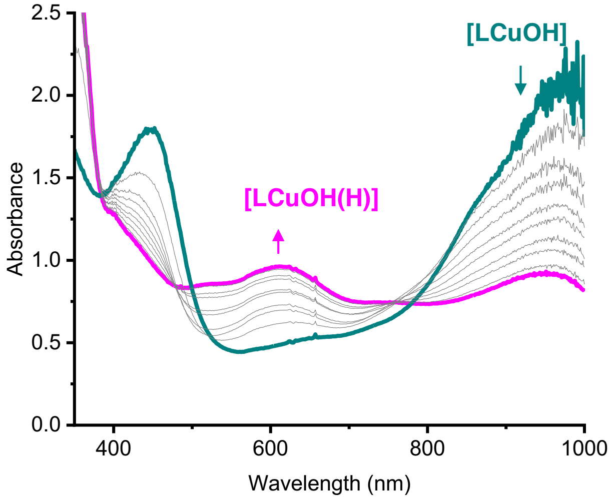

Figure S67. UV-vis spectra of the reaction between **[LCuOH]** (turquoise) and 20 equi of TEMPOH under Ar at -40 °C. Note the spectrum only shows the reaction up until the formation of **[LCuOH(H)]**

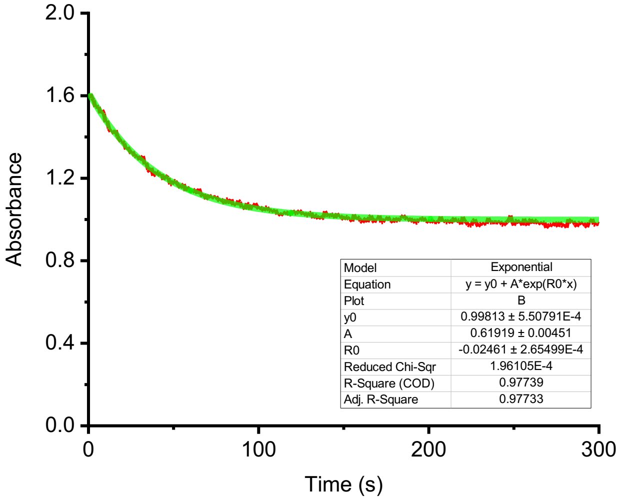

Figure S68. UV-vis time trace at 965 nm for the reaction of **[LCuOH]** and 20 equiv of TEMPOH under Ar at -40 °C. Exponential fitting shown in green.

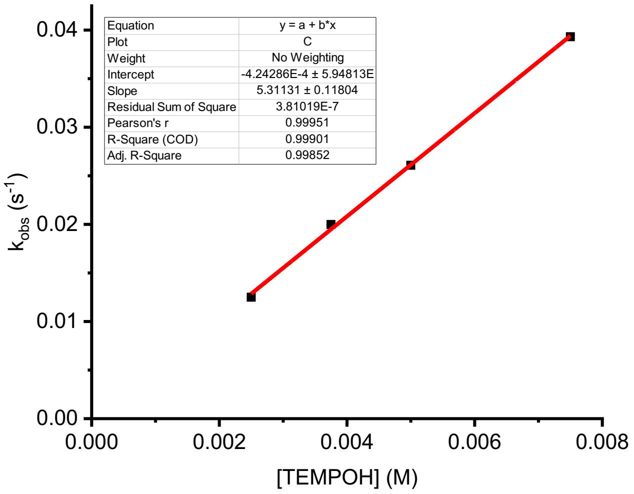

Figure S69. Second order rate constant for the reaction between **[LCuOH]** and TEMPOH at -40 °C (k_2 = 5.3 $M^{-1}s^{-1}$)

6.8. Kinetics for the reaction of [LCuOH] with TEMPOD

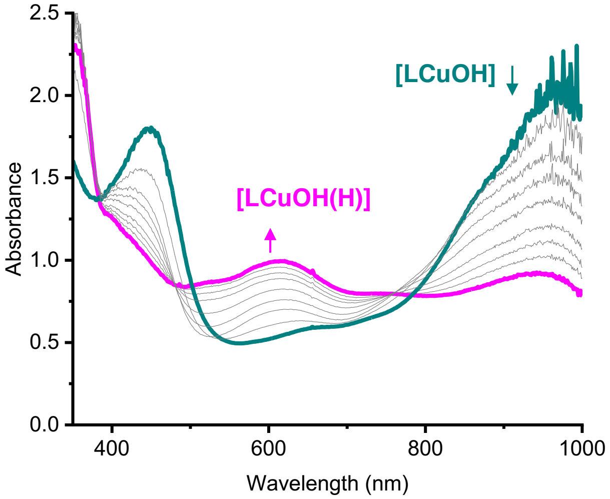

Figure S70. UV-vis spectra of the reaction between **[LCuOH]** (turquoise) and 20 equiv of TEMPOD under Ar at -40 °C. Note the spectrum only shows the reaction up until the formation of **[LCuOH(H)].**

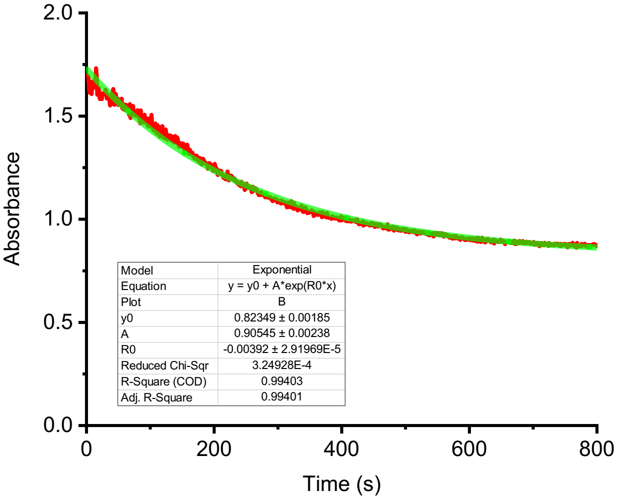

Figure S71. UV-vis time trace at 965 nm for the reaction of **[LCuOH]** and 20 equiv of TEMPOD under Ar at -40 °C. Exponential fitting shown in green.

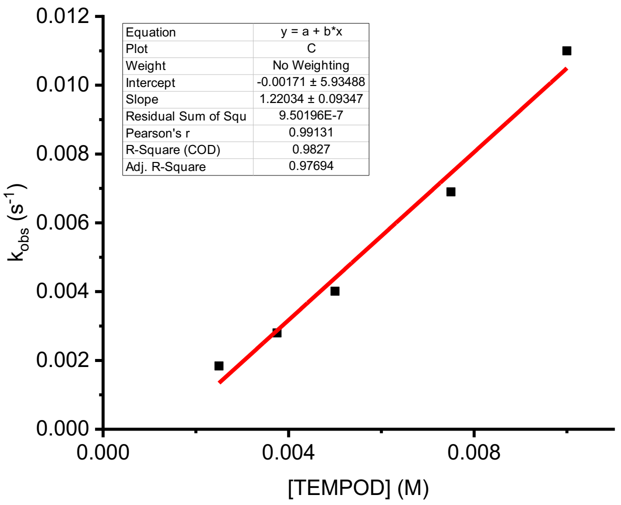

Figure S72. Second order rate constant for the reaction between **[LCuOH]** and TEMPOD at -40 °C (k_2 = 1.22 $M^{-1}s^{-1}$)

6.9. Kinetics Summary

Table S5. Summary of the kinetics between **[MOH]$^{n-}$** and TEMPOH/D.

Complex	[TEMPOH/D] (M)	k_{obs} (TEMPOH) (s^{-1})	k_{obs} (TEMPOD) (s^{-1})
**[LNiOH]**	0.005	0.00067	-
	0.0075	0.00087	0.00028
	0.01	0.0011	-
	0.0125	0.0014	0.00039
	0.015	-	0.00047
**[LNiOH]$^-$**	0.0025	0.066	0.0042
	0.00375	0.095	0.0062
	0.005	0.133	0.0077
	0.0075	0.195	0.0116
**[CuOH]**	0.0025	0.0125	0.0018
	0.00375	0.0199	0.0028
	0.005	0.0261	0.0040
	0.0075	0.0393	0.0069
	0.01	-	0.0110
**[CuOH]$^-$**	0.0025	0.0074	-
	0.005	0.0155	0.0081
	0.0075	0.023	0.0124
	0.01	0.033	0.0154

Concentration of **[MOH]$^{n-}$** = 0.25 mM. All experiments were performed at -40 ^oC with the exception of **[LCuOH]$^-$**, which was performed at room temperature.

6.10. Eyring plot analysis

6.10.1. Eyring plot for the reaction of [LNiOH]⁻ with TEMPOH

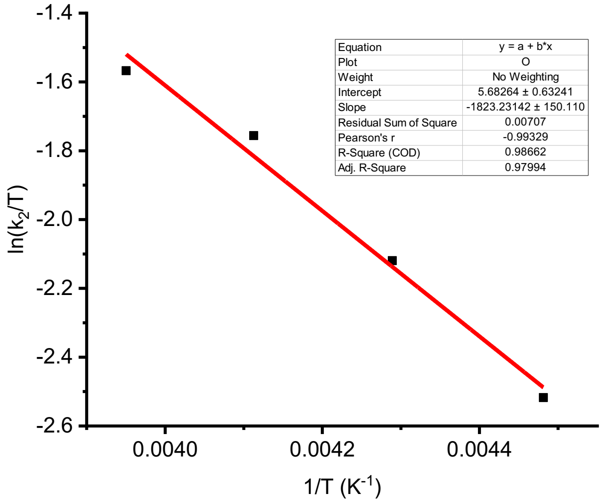

Figure S73. Eyring plot for the reaction between **[LNiOH]⁻** and TEMPOH. $\Delta H^{\ddagger}$ = 3.6 kcal•mol^{-1}, $\Delta S^{\ddagger}$ = -36.0 cal•mol^{-1}K^{-1}, $\Delta G^{\ddagger}_{233}$ = 12.0 kcal•mol^{-1}.

6.10.2. Eyring plot for the reaction of [LNiOH] with TEMPOH

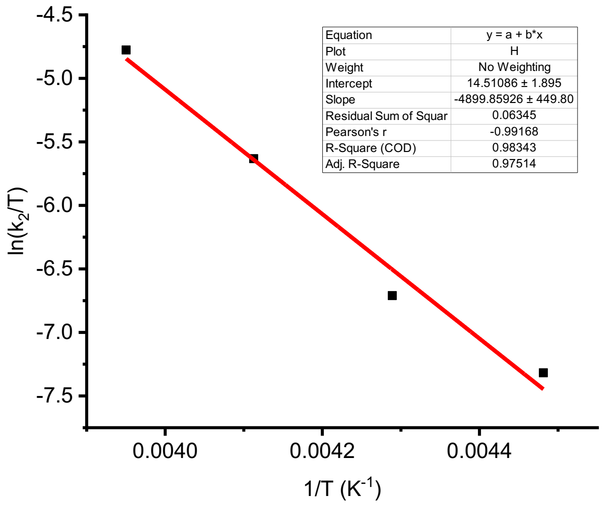

Figure S74. Eyring plot for the reaction between **[LNiOH]** and TEMPOH. $\Delta H^{\ddagger}$ = 9.7 kcal•mol^{-1}, $\Delta S^{\ddagger}$ = -18.4 cal•mol^{-1}K^{-1}, $\Delta G^{\ddagger}_{233}$ = 14.0 kcal•mol^{-1}.

6.10.3. Eyring plot for the reaction of $[LCuOH]^-$ with TEMPOH

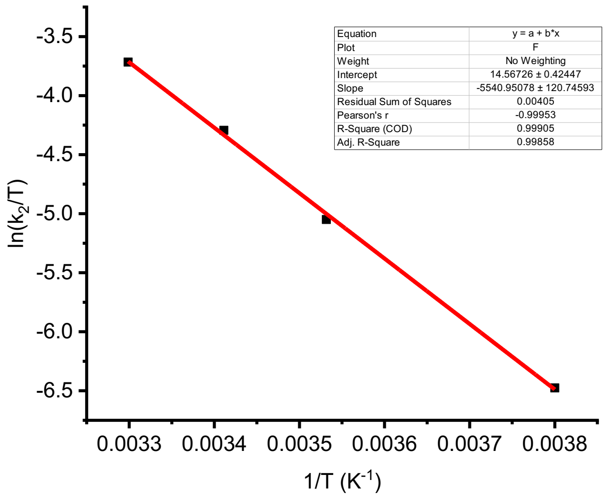

Figure S75. Eyring plot for the reaction between **[LCuOH]$^-$** and TEMPOH. $\Delta H^‡$ = 11 kcal•mol^{-1}, $\Delta S^‡$ = -18.3 cal•mol^{-1}K^{-1}, $\Delta G^‡_{233}$ = 15.3 kcal•mol^{-1}.

6.10.4. Eyring plot for the reaction of [LCuOH] with TEMPOH

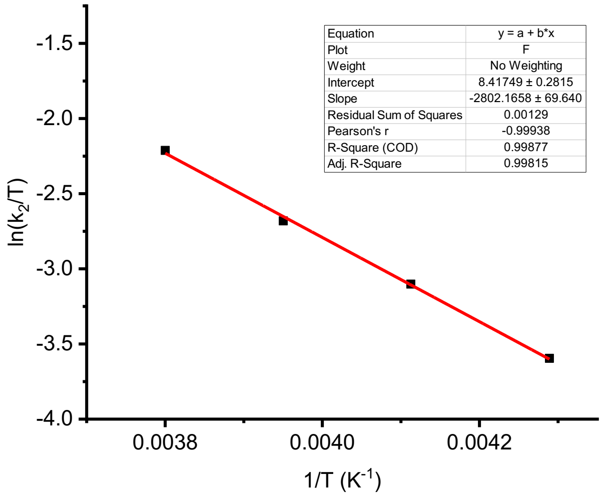

Figure S76. Eyring plot for the reaction between **[LCuOH]** and TEMPOH. $\Delta H^‡$ = 5.6 kcal•mol^{-1}, $\Delta S^‡$ = -30.5 cal•mol^{-1}K^{-1}, $\Delta G^‡_{233}$ = 12.7 kcal•mol^{-1}.

7. DFT Calculations

All DFT calculations were performed with the Amsterdam Density Functional (ADF)[12,13] and QUILD6 programs, and were performed using the unrestricted Kohn-Sham scheme. Molecular orbitals were expanded in an uncontracted set of Slater type orbitals (STOs) of triple-ζ quality with double polarization functions (TZ2P), or the TDZP basis set which consists of triple-ζ quality on the metal and double-ζ quality on all other atoms, in both cases including one polarization function.[14,15] Core electrons were not treated explicitly during the geometry optimizations (frozen core approximation[13]). An auxiliary set of s, p, d, f, and g STOs was used to fit the molecular density and to represent the coulomb and exchange potentials accurately for each SCF cycle.

Geometries of all possible spin states were optimized with the QUILD6 program using adapted delocalized coordinates until the maximum gradient component was less than 10–4 a.u. Energies, gradients and Hessians9 (for vibrational frequencies) were calculated using BP86-D3 [16–21], in all cases by including solvation effects through the COSMO13 dielectric continuum model with appropriate parameters for the solvents.[22] For computing Gibbs free energies, all small frequencies were raised to 100 cm–1 in order to compensate for the breakdown of the harmonic oscillator model.[23,24] Scalar relativistic corrections have been included self-consistently in all calculations by using the zerothorder regular approximation (ZORA[25]). The geometry optimizations (with the TZ2P basis set) have been performed with S12g with a Becke grid of VeryGood quality.

All computational data will be uploaded onto the IOCHEM-BD platform (www.iochem-bd.org[26]) to facilitate data exchange and dissemination, according to the FAIR principles[27] of OpenData sharing. All compdata is available to the general public following this link or can be downloaded as a zip file:

https://doi.org/10.19061/iochem-bd-4-79

7.1 Computations on [LNiOH(DMF)$_2$]

Like we stated in the manuscript, the computations on [LNiOH] in the triplet state suggested that this species should be formulated as a Ni^{III} semiquinone-like complex. This is evidenced by the d-electron occupancy (7 of out of 10 of the electrons in the d-orbitals had higher occupancy than 0.7) and by the substantial spin density located on the aromatic C atoms of the ligand scaffold. However, this disagrees with our experimental data (NMR, UV-Vis), which suggest that the [LNiOH] is in the triplet state at room temperature and that is formulated as a Ni^{II}OH core bound by a quinone-like ligand. We hypothesized that the coordination of DMF molecules could induce a change in the electronic structure of the [LNiOH] complex. The computations of [LNiOH(DMF)$_2$] were carried out for the single and triplet isomers (see Figure S77) and we observed that the two molecules of DMF coordinated to the complex via intramolecular H-bonding interactions between the ureanyl H-atom donor and the O-atom of the solvent. DMF coordination induced a change in the d-orbital occupancy values of the Ni complex in the triplet state (now 8 of out of 10 of the electrons in the d-orbitals had higher occupancy than 0.7), a substantial decrease on the spin density on the aromatic C atoms (from 70.4% to 52.7%) and an increase on the spin density on the Ni (from 46.2% to 57.1%), which suggest that [LNiOH(DMF)$_2$] is formulated as a Ni^{II} quinone-like species. Computations on [LNiOH(DMF)$_2$]$^-$ and [LNiOH(DMF)$_2$]$^{2-}$ (singlet and triplet state) were also performed, but no significant changes in the d-orbital occupancy and spin-density plots were observed.

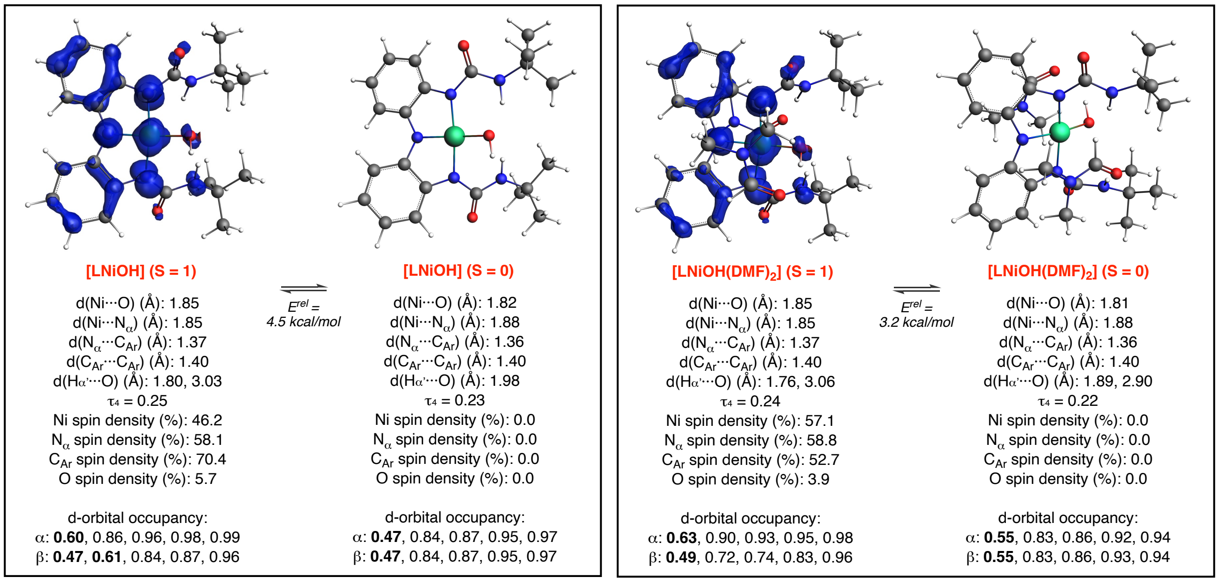

Figure S77. DFT calculations on the electronic structure of the [LNiOH(DMF)$_2$] in the singlet and triplet state.

8. References

1. Wu, T.; MacMillan, S. N.; Rajabimoghadam, K.; Siegler, M. A.; Lancaster, K. M.; Garcia-Bosch, I. Structure, Spectroscopy, and Reactivity of a Mononuclear Copper Hydroxide Complex in Three Molecular Oxidation States. *J. Am. Chem. Soc.* **2020**, *142*, 28, 12265–12276

2. Favier, I.; Duñach, E. New protic salts of aprotic polar solvents, *Tetrahedron Letters*, Volume 45, Issue 17, 2004, Pages 3393-3395, ISSN 0040-4039

3. Brazzolotto, D.; Bogart, J. A.; Ross, D. L.; Ziller, J. W.; Borovik, A. S. Stabilizing a NiII-aqua complex via intramolecular hydrogen bonds: Synthesis, structure, and redox properties. *Inorg. Chim. Acta* **2019**, *495*, 118960.

4. VanderWeide, A., Prokopchuk, D.E. Cyclopentadienyl ring activation in organometallic chemistry and catalysis. *Nat Rev Chem,* **2023***, 7*, 561–572.

5. Wu, T.; Puri, A.; Qiu, Y. L.; Ye, D.; Sarma, R.; Wang, Y.; Kowalewski, T.; Siegler, M. A.; Swart, M.; Garcia-Bosch, I. Tuning the Thermochemistry and Reactivity of a Series of Cu-Based $4H^+/4e^-$ Electron-Coupled-Proton-Buffers. *Inorg. Chem.,* **2024**, *63*, 9014-9025

6. Hill, E. A.; Weitz, A. C.; Onderko, E.; Romero-Rivera, A.; Guo, Y.; Swart, M.; Bominaar, E. L.; Green, M. T.; Hendrich, M. P.; Lacy, D. C.; et al. Reactivity of an FeIV-Oxo Complex with Protons and Oxidants. *J. Am. Chem. Soc.* **2016**, *138* (40), 13143-13146.

7. Rosenkoetter, K. E.; Wojnar, M. K.; Charette, B. J.; Ziller, J. W.; Heyduk, A. F. Hydrogen-Atom Noninnocence of a Tridentate [SNS] Pincer Ligand. *Inorg. Chem.* **2018**, *57* (16), 9728-9737.

8. Holz, R. C.; Evdokimov, E. A.; Gobena, F. T. Two Dimensional ^{1}H NMR Studies on Octahedral Nickel(II) Complexes. *Inorg. Chem.* **1996**, *35*, 3808-3814

9. Wicholas, M.; Drago, R. S.; Spin-Delocalization Mechanisms in Some Paramagnetic Tris-2,2'-bipyridine Complexes of Nickel(II). *J. Am. Chem. Soc.,* **1968**, *90*, 6946-6950

10. Agarwal, R. G.; Coste, S. C.; Groff, B. D.; Heuer, A. M.; Noh, H.; Parada, G. A.; Wise, C. F.; Nichols, E. M.; Warren, J. J.; Mayer, J. M. Free Energies of Proton-Coupled Electron Transfer Reagents and Their Applications. *Chem. Rev.,* **2022**, *122*, 1-49

11. Chang, R. *Physical Chemistry for the Biosciences.* University Science Books, **2005**, 338-342.

(12) Baerends, E. J.; Ziegler, T.; Autschbach, J.; Bashford, D.; Berger, A.; Bérces, A.; Bickelhaupt, F. M.; Bo, C.; de Boeij, P. L.; Boerrigter, P. M.; Borini, S.; Bulo, R. E.; Cavallo, L.; Chong, D. P.; Deng, L.; Dickson, R. M.; van Duin, A. C. T.; Ellis, D. E.; van Faassen, M.; Fan, L.; Fischer, T. H.;

Fonseca Guerra, C.; Ghysels, A.; Giammona, A.; van Gisbergen, S. J. A.; Götz, A. W.; Groeneveld, J. A.; Gritsenko, O. V.; Grüning, M.; Gusarov, S.; Harris, F. E.; Heine, T.; van den Hoek, P.; Jacob, C. R.; Jacobsen, H.; Jensen, L.; Kadantsev, E. S.; Kaminski, J. W.; van Kessel, G.; Klooster, R.; Kootstra, F.; Kovalenko, A.; Krykunov, M. V.; van Lenthe, E.; Louwen, J. N.; McCormack, D. A.; McGarrity, E.; Michalak, A.; Mitoraj, M.; Neugebauer, J.; Nicu, V. P.; Noodleman, L.; Osinga, V. P.; Patchkovskii, S.; Philipsen, P. H. T.; Post, D.; Pye, C. C.; Ravenek, W.; Rodríguez, J. I.; Romaniello, P.; Ros, P.; Schipper, P. R. T.; Schreckenbach, G.; Seldenthuis, J. S.; Seth, M.; Skachkov, D. G.; Snijders, J. G.; Solà, M.; Swart, M.; Swerhone, D.; te Velde, G.; Vernooijs, P.; Versluis, L.; Visscher, L.; Visser, O.; Wang, F.; Wesolowski, T. A.; van Wezenbeek, E. M.; Wiesenekker, G.; Wolff, S. K.; Woo, T. K.; Yakovlev, A. L. ADF2012.01; SCM, Theoretical Chemistry, Vrije Universiteit, Amsterdam, 2012.

(13) te Velde, G.; Bickelhaupt, F. M.; Baerends, E. J.; Fonseca Guerra, C.; van Gisbergen, S. J. A.; Snijders, J. G.; Ziegler, T. Chemistry with ADF. *J. Comput. Chem.* **2001**, *22* (9), 931–967.

(14) Swart, M.; Bickelhaupt, F. M. QUILD: QUantum-Regions Interconnected by Local Descriptions. *J. Comput. Chem.* **2008**, *29* (5), 724–734.

(15) Van Lenthe, E.; Baerends, E. J. Optimized Slater-Type Basis Sets for the Elements 1-118. *J. Comput. Chem.* **2003**, *24* (9), 1142–1156.

(16) Chong, D. P.; van Lenthe, E.; Van Gisbergen, S.; Baerends, E. J. Even-Tempered Slater-Type OrbitalsRevisited: From Hydrogen to Krypton. *J. Comput. Chem.* **2004**, *25* (8), 1030–1036.

(17) Wolff, S. K. Analytical Second Derivatives in the Amsterdam Density Functional Package. Int. *J. Quantum Chem.* **2005**, *104* (5), 645–659.

(18) Becke, A. D. Density-Functional Exchange-Energy Approximation with Correct Asymptotic Behavior. *Phys. Rev. A Gen. Phys.* **1988**, *38* (6), 3098–3100.

(19) Perdew, J. P. Density-Functional Approximation for the Correlation Energy of the Inhomogeneous Electron Gas. *Phys. Rev. B Condens. Matter* **1986**, *33* (12), 8822-8824 Erratum: Ibid. 8834, 7406.

(20) Grimme, S.; Antony, J.; Ehrlich, S.; Krieg, H. A Consistent and Accurate Ab Initio Parametrization of Density Functional Dispersion Correction (DFT-D) for the 94 Elements H-Pu. *J. Chem. Phys.* **2010**, *132* (15), 154104.

(21) Klamt, A.; Schüürmann, G. COSMO: A New Approach to Dielectric Screening in Solvents with Explicit Expressions for the Screening Energy and Its Gradient. *Journal of the Chemical Society, Perkin Transactions 2* **1993**, *0* (5), 799–805.

(22) Swart, M.; Rösler, E.; Bickelhaupt, F. M. Proton Affinities in Water of Maingroup-element Hydrides – Effects of Hydration and Methyl Substitution. *Eur. J. Inorg. Chem.* **2007**, *2007* (23), 3646–3654.

(23) Averkiev, B. B.; Truhlar, D. G. Free Energy of Reaction by Density Functional Theory: Oxidative Addition of Ammonia by an Iridium Complex with PCP Pincer Ligands. *Catalysis Science & Technology* **2011**, *1* (8), 1526–1529.

(24) Klein, J. E. M. N.; Dereli, B.; Que, L.; Cramer, C. J. Why Metal-Oxos React with Dihydroanthracene and Cyclohexadiene at Comparable Rates, despite Having Different C-H Bond Strengths. A Computational Study. *Chem. Commun. (Camb.)* **2016**, *52* (69), 10509–10512.

(25) van Lenthe, E.; Baerends, E. J.; Snijders, J. G. Relativistic Regular Two-Component Hamiltonians. *J. Chem. Phys.* **1993**, 99 (6), 4597–4610.

(26) Álvarez-Moreno, M.; de Graaf, C.; López, N.; Maseras, F.; Poblet, J. M.; Bo, C. Managing the Computational Chemistry Big Data Problem: The IoChem-BD Platform. *J. Chem. Inf. Model.* **2015**, *55* (1), 95–103.

(27) Wilkinson, M. D.; Dumontier, M.; Aalbersberg, I. J. J.; Appleton, G.; Axton, M.; Baak, A.; Blomberg, N.; Boiten, J.-W.; da Silva Santos, L. B.; Bourne, P. E.; Bouwman, J.; Brookes, A. J.; Clark, T.; Crosas, M.; Dillo, I.; Dumon, O.; Edmunds, S.; Evelo, C. T.; Finkers, R.; Gonzalez-Beltran, A.; Gray, A. J. G.; Groth, P.; Goble, C.; Grethe, J. S.; Heringa, J.; ’t Hoen, P. A. C.; Hooft, R.; Kuhn, T.; Kok, R.; Kok, J.; Lusher, S. J.; Martone, M. E.; Mons, A.; Packer, A. L.; Persson, B.; Rocca-Serra, P.; Roos, M.; van Schaik, R.; Sansone, S.-A.; Schultes, E.; Sengstag, T.; Slater, T.; Strawn, G.; Swertz, M. A.; Thompson, M.; van der Lei, J.; van Mulligen, E.; Velterop, J.; Waagmeester, A.; Wittenburg, P.; Wolstencroft, K.; Zhao, J.; Mons, B. The FAIR Guiding Principles for Scientific Data Management and Stewardship. *Sci. Data* **2016**, 3, 160018.
