# Supplementary material for: Enhanced Proton-Coupled Electron-Transfer Reactivity by a Mononuclear Nickel(II) Hydroxide Radical Complex
Source: Inorg Chem. 2024 Dec 16;63(52):24453–65. doi: 10.1021/acs.inorgchem.4c03370 (PMC11688665; doi:10.1021/acs.inorgchem.4c03370)
Supplement: Supplementary file 2 — ic4c03370_si_002.zip [file ic4c03370_si_002.zip › CompData/CompChemData_for_review.pdf]

SYSTEM NIOHDMF2_0_1 2
SYSTEM NIOHDMF2_0_3 4
SYSTEM NIOHDMF2_-1_2 6
SYSTEM NIOHDMF2_-2_1 8
SYSTEM NIOHDMF2_-2_3 10
SYSTEM NI-OH_0_1 12
SYSTEM NI-OH_0_3 14
SYSTEM NI-OH_-1_2 16
SYSTEM NI-OH_-2_1 18
SYSTEM NI-OH_-2_3 20
SYSTEM NIOH-CS_0_1 (CLOSED-SHELL SINGLET, C_S SYMMETRY) 22
SYSTEM NIOH-CS-2_0_1 (OPEN-SHELL SINGLET, C_S SYMMETRY) 24
SYSTEM NIOH-CS_0_3 (TRIPLET, C_S SYMMETRY) 26

System niohdmf2_0_1

```
Reading 1 outputfiles

       Pauli        Elstat         OrbInt       Disp.       Solv.         TOTAL          Erel    Symm.      <S2>
----------------------------------------------------------------------------------------------------------------------
-----
   44368.946     -9293.840     -46880.909     -61.882     -19.180    -11887.092         0.000    NOSYM     0.000
AMS(1)

Corresponding output files
 1 : opt_niohdmf2_0_1.12323827.out

Coordinates (Angs)
  84
opt_niohdmf2_0_1.12323827.out       -11887.0934
O       2.725610    3.079434   -0.843090
C      -4.633593    1.811205   -0.229666
H      -3.811125    2.533028   -0.189656
H      -5.553259    2.320581    0.072612
H      -4.747382    1.478727   -1.267396
C      -4.163086    1.133589    2.131595
H      -3.977508    0.301063    2.815573
H      -5.055455    1.671751    2.468525
H      -3.299444    1.805134    2.170663
C      -5.537967   -0.356001    0.629208
H      -5.682255   -0.704824   -0.399354
H      -6.459408    0.135337    0.959520
H      -5.355040   -1.223128    1.268816
C      -4.368935    0.628957    0.700370
C      -2.495933   -1.032973    0.798361
C      -0.808696   -2.439825   -0.205839
C      -1.620214   -3.593563   -0.362662
H      -2.657802   -3.563852   -0.045600
C      -1.090671   -4.717117   -0.948691
H      -1.722648   -5.591416   -1.091395
C       0.248110   -4.753987   -1.403901
H       0.618432   -5.638537   -1.915781
C       1.077986   -3.670960   -1.228077
H       2.085864   -3.693833   -1.626045
C       0.578664   -2.507962   -0.602217
C       2.567830   -1.076135   -0.303187
C       3.614352   -2.021622   -0.285347
H       3.394550   -3.082039   -0.245729
C       4.919870   -1.589358   -0.252029
H       5.729631   -2.313817   -0.216135
C       5.207305   -0.209829   -0.228059
H       6.245163    0.115881   -0.190976
C       4.208726    0.740209   -0.196111
H       4.446854    1.793194   -0.127121
C       2.853540    0.335094   -0.191513
C       1.734918    2.481011   -0.405770
C       0.125612    4.412965   -0.587749
C       0.377047    4.707713   -2.069684
H       1.437801    4.615360   -2.314406
H       0.051394    5.726726   -2.303619
H      -0.192198    4.014309   -2.698066
C       0.886638    5.403190    0.298620
H       0.693846    5.194634    1.356823
H       0.561255    6.426887    0.084042
H       1.963000    5.334452    0.119594
C      -1.374715    4.497175   -0.305218
H      -1.917109    3.773446   -0.923805
H      -1.745117    5.500769   -0.533447
H      -1.589968    4.284644    0.748838
N      -3.135382   -0.009605    0.215791
H      -2.642550    0.457257   -0.542447
N      -1.200783   -1.238844    0.234718
N       1.227724   -1.336411   -0.362654
N       1.749637    1.115010   -0.053097
N       0.507345    3.034561   -0.267885
H      -0.191568    2.466338    0.211755
O      -2.921027   -1.736364    1.717990
Ni      0.229160    0.017498    0.322260
O      -0.651021    1.121980    1.456906
H       0.032281    1.505240    2.035442
O      -1.871186    1.268677   -2.238770
C      -0.776661    0.911497   -2.690737
N      -0.504581   -0.271823   -3.265445
C       0.815063   -0.570148   -3.773405
C      -1.527059   -1.279019   -3.441410
H       0.108974    1.571834   -2.650796
H       1.198842   -1.489275   -3.315371
H       1.497387    0.250239   -3.535775
H       0.791536   -0.709420   -4.860932
H      -2.425585   -0.973546   -2.903055
H      -1.178066   -2.240315   -3.049171
H      -1.764305   -1.404514   -4.505157
O       1.687909    1.065223    3.523788
C       2.022429   -0.089300    3.230146
N       1.264468   -1.191192    3.361050
C       1.762131   -2.493034    2.978519
C      -0.077686   -1.136802    3.900735
```

```
H      -0.147112   -1.777031    4.788394
H      -0.803373   -1.478985    3.153832
H      -0.313002   -0.106908    4.169645
H       1.780100   -3.167729    3.842596
H       2.775537   -2.402205    2.579830
H       1.118047   -2.936550    2.208994
H       3.019878   -0.315066    2.806101

Frequencies (cm-1)
  100.00    100.00    100.00    100.00    100.00    100.00    100.00    100.00    100.00    100.00
  100.00    100.00    100.00    100.00    100.00    100.00    100.00    100.00    100.00    100.41
  102.01    108.34    119.09    123.79    124.34    128.18    142.41    157.38    168.08    174.11
  176.10    187.26    195.60    206.66    212.96    215.85    221.43    225.42    247.02    250.02
  252.29    257.18    259.41    270.28    271.90    281.10    299.31    310.46    318.57    318.88
  324.93    326.11    337.97    352.67    355.09    356.14    360.61    366.88    371.64    391.66
  399.34    399.49    412.59    414.88    434.51    446.49    461.84    466.34    467.75    472.28
  488.08    489.16    508.51    516.25    566.00    577.12    587.66    595.13    607.29    650.26
  652.34    665.55    675.66    716.27    725.19    730.92    737.00    744.77    749.06    757.33
  771.23    776.15    804.31    818.69    831.92    843.95    861.70    863.35    866.23    867.75
  888.88    902.56    911.06    912.41    916.46    918.29    924.02    931.84    933.62    933.74
  936.13    945.77    949.29    968.47    972.86    977.00    979.72   1010.16   1018.67   1020.46
 1021.32   1022.64   1036.44   1037.35   1043.90   1044.14   1044.54   1076.50   1076.68   1081.13
 1083.66   1130.51   1132.73   1133.13   1133.98   1140.46   1151.90   1197.93   1205.43   1210.32
 1212.51   1214.86   1215.69   1218.21   1235.26   1242.29   1249.74   1252.15   1267.72   1290.50
 1326.45   1333.73   1336.03   1340.16   1341.28   1357.35   1366.25   1366.63   1367.47   1372.26
 1375.41   1380.32   1383.76   1386.93   1389.18   1391.13   1393.98   1407.60   1409.12   1410.80
 1414.53   1414.94   1418.03   1425.89   1427.41   1428.42   1429.21   1431.85   1432.65   1433.20
 1436.24   1437.77   1440.35   1441.68   1444.28   1445.71   1461.66   1463.10   1464.76   1466.30
 1484.54   1488.52   1489.18   1532.73   1537.66   1539.10   1547.97   1589.21   1601.34   1654.66
 1666.89   1670.27   1688.42   2930.13   2956.15   2977.86   2980.02   2985.24   2986.79   2986.95
 2991.12   2992.03   2993.87   2997.83   2999.14   3058.35   3062.19   3070.70   3072.57   3084.58
 3089.71   3089.82   3093.21   3096.07   3098.73   3104.02   3105.49   3107.24   3108.89   3109.02
 3109.59   3112.93   3116.90   3128.49   3137.21   3140.59   3142.06   3157.38   3158.62   3179.51
 3183.10   3197.75   3215.35   3341.34   3441.08   3647.96
Note: any frequencies below 100 cm-1 (including spurious imaginary ones) are upscaled to 100 cm-1
      for the calculation of thermodynamic properties.
      (Averkiev, Truhlar, Catal. Sci. Technol. 2011, 1, 1526)

Thermodynamics

Note: this script does not take into account the spin entropy
For more info, see eq. 3 of Inorg. Chem. 2002, 41, 6928-6935
(M. Reiher), https://doi.org/10.1021/ic025891l

Temperature is now: 298.150

Reporting max. of 0 frequencies (set by $GETFREQSMAX env. variable)


Reading 1 outputfiles

               AMS(1)
---------------------
---------------------
(ZPVE)        430.111
(dH,0->T)      29.418
(-TS)         -74.833
(dGibbs)      384.696
=====================

Corresponding output files
 1 : frq_niohdmf2_0_1.12365812.out
```

System niohdmf2_0_3

```
Reading 1 outputfiles

       Pauli        Elstat        OrbInt       Disp.       Solv.         TOTAL          Erel   Symm.      <S2>
---------------------------------------------------------------------------------------------------------------------
-----
   44359.585     -9293.405    -46869.963     -62.018     -17.831    -11883.856         0.000   NOSYM     2.007
AMS(1)

Corresponding output files
 1 : opt_niohdmf2_0_3.9938484.out

Coordinates (Angs)
  84
opt_niohdmf2_0_3.9938484.out        -11883.8565
O       2.046241    3.276748   -1.112968
C      -4.717974    1.255028   -0.705608
H      -3.976241    2.060953   -0.699315
H      -5.700946    1.689909   -0.503159
H      -4.733109    0.815866   -1.709076
C      -4.362515    0.848492    1.738768
H      -4.128624    0.108095    2.508558
H      -5.337799    1.292532    1.964228
H      -3.594822    1.627822    1.774276
C      -5.429676   -0.923534    0.294929
H      -5.447329   -1.379310   -0.701151
H      -6.425563   -0.520818    0.507713
H      -5.203392   -1.700116    1.029995
C      -4.394030    0.201083    0.351479
C      -2.363111   -1.217642    0.724864
C      -0.493353   -2.476777   -0.197755
C      -1.199033   -3.675609   -0.410004
H      -2.234934   -3.753192   -0.093399
C      -0.565604   -4.732094   -1.038094
H      -1.111399   -5.655853   -1.215942
C       0.764938   -4.617367   -1.470397
H       1.236658   -5.448050   -1.990013
C       1.490010   -3.454769   -1.254986
H       2.502086   -3.379664   -1.634101
C       0.888218   -2.379826   -0.582917
C       2.695752   -0.683344   -0.330744
C       3.884868   -1.417638   -0.457093
H       3.866595   -2.499432   -0.513451
C       5.101929   -0.753288   -0.445599
H       6.022454   -1.327084   -0.526162
C       5.155866    0.638674   -0.306105
H       6.119834    1.142559   -0.286212
C       3.995305    1.384484   -0.164631
H       4.040181    2.458469   -0.026232
C       2.750990    0.738888   -0.167445
C       1.272132    2.671127   -0.364620
C      -0.528824    4.414786   -0.257047
C      -0.830082    4.440571   -1.759617
H       0.094197    4.390699   -2.342169
H      -1.352404    5.367638   -2.018990
H      -1.464229    3.589767   -2.031341
C       0.357497    5.597032    0.141916
H       0.565333    5.573730    1.217327
H      -0.151600    6.538502   -0.090705
H       1.307407    5.570262   -0.397694
C      -1.840762    4.466476    0.526269
H      -2.483700    3.617730    0.267443
H      -2.380454    5.388612    0.291958
H      -1.656103    4.440807    1.606235
N      -3.056573   -0.318936    0.019462
H      -2.554875    0.149319   -0.734392
N      -1.001655   -1.315300    0.285822
N       1.410163   -1.156117   -0.276836
N       1.519604    1.326201    0.040735
N       0.104577    3.143350    0.106173
H      -0.430634    2.494540    0.702248
O      -2.764333   -1.905845    1.663329
Ni      0.227447    0.059582    0.456479
O      -0.821218    1.040569    1.617913
H      -0.202947    1.251744    2.341751
O      -1.760229    0.981090   -2.350507
C      -0.598745    0.830392   -2.749788
N      -0.098341   -0.276388   -3.321147
C       1.279382   -0.336179   -3.754649
C      -0.925753   -1.435467   -3.575444
H       0.154474    1.635201   -2.663257
H       1.793491   -1.174800   -3.270376
H       1.792783    0.590436   -3.484742
H       1.338477   -0.474111   -4.840932
H      -1.895510   -1.294265   -3.095833
H      -0.447244   -2.334273   -3.172871
H      -1.070060   -1.572521   -4.654326
O       1.515920    1.008009    3.643409
C       2.055845   -0.051029    3.296660
N       1.494845   -1.270999    3.335165
C       2.212204   -2.442342    2.884429
C       0.155152   -1.478106    3.844356
```

```
H       0.175187   -2.230905    4.640719
H      -0.513656   -1.819049    3.045169
H      -0.226971   -0.536336    4.239510
H       2.347824   -3.151411    3.709559
H       3.192809   -2.153250    2.498131
H       1.654568   -2.944547    2.084778
H       3.087951   -0.074112    2.898059

Frequencies (cm-1)
   100.00    100.00    100.00    100.00    100.00    100.00    100.00    100.00    100.00    100.00
   100.00    100.00    100.00    100.00    100.00    100.00    100.00    100.00    100.00    100.15
   104.37    112.42    120.49    122.36    126.93    134.30    146.33    159.62    174.33    177.59
   180.07    192.08    195.43    202.98    213.09    216.76    225.74    227.88    246.15    252.26
   257.36    258.95    261.24    267.31    269.46    274.71    293.89    316.08    317.44    320.71
   323.69    328.84    336.42    351.69    353.83    354.38    365.22    368.56    387.39    388.53
   399.91    399.93    410.13    415.34    441.27    444.49    458.46    466.79    469.14    470.77
   484.27    517.38    524.26    528.51    535.59    572.94    581.14    590.85    606.07    650.45
   652.65    668.28    679.81    721.89    725.98    727.61    734.07    739.89    744.59    756.13
   768.88    802.17    817.69    830.19    836.86    854.09    862.21    866.58    872.85    880.79
   893.44    905.41    911.14    912.87    915.87    918.77    919.65    921.60    924.80    932.52
   933.88    946.82    958.79    959.60    961.95    976.97    982.56   1001.87   1015.13   1018.44
  1019.56   1030.28   1034.11   1037.80   1043.72   1044.39   1047.97   1077.09   1078.10   1082.88
  1083.78   1124.46   1128.02   1131.90   1133.17   1146.02   1151.42   1195.24   1208.22   1211.16
  1211.65   1215.23   1216.23   1217.93   1239.74   1242.62   1248.34   1251.49   1270.54   1290.27
  1294.72   1333.43   1334.57   1339.03   1340.29   1342.79   1359.63   1366.87   1368.71   1374.11
  1374.62   1375.12   1379.45   1385.33   1386.24   1391.34   1391.94   1409.98   1410.96   1411.34
  1415.44   1416.03   1418.83   1425.49   1427.45   1427.64   1430.06   1433.29   1434.17   1435.79
  1436.62   1437.94   1440.39   1440.66   1443.61   1445.51   1450.37   1457.74   1461.03   1462.47
  1463.94   1488.70   1489.58   1536.14   1549.39   1556.50   1562.46   1568.19   1580.89   1663.20
  1667.04   1667.84   1696.36   2941.75   2976.11   2978.94   2979.96   2987.82   2988.09   2989.29
  2991.65   2991.96   2993.58   2996.92   2999.48   3060.54   3060.58   3074.08   3074.46   3084.13
  3089.83   3090.47   3091.47   3092.42   3100.23   3103.51   3105.56   3105.94   3109.62   3109.89
  3111.32   3112.51   3115.21   3128.04   3137.73   3139.60   3141.25   3143.23   3154.83   3156.85
  3169.90   3186.97   3199.96   3204.96   3400.50   3605.23
Note: any frequencies below 100 cm-1 (including spurious imaginary ones) are upscaled to 100 cm-1
      for the calculation of thermodynamic properties.
      (Averkiev, Truhlar, Catal. Sci. Technol. 2011, 1, 1526)

Thermodynamics

Note: this script does not take into account the spin entropy
For more info, see eq. 3 of Inorg. Chem. 2002, 41, 6928-6935
(M. Reiher), https://doi.org/10.1021/ic025891l

Temperature is now: 298.150

Reporting max. of 0 frequencies (set by $GETFREQSMAX env. variable)


Reading 1 outputfiles

               AMS(1)
---------------------
---------------------
(ZPVE)        429.788
(dH,0->T)      29.347
(-TS)         -74.630
(dGibbs)      384.505
=====================

Corresponding output files
 1 : frq_niohdmf2_0_3.12299161.out
```

System niohdmf2_-1_2

```
Reading 1 outputfiles

       Pauli        Elstat        OrbInt       Disp.       Solv.         TOTAL          Erel   Symm.      <S2>
-----------------------------------------------------------------------------------------------------------------------
-----
   44239.569     -9270.522    -46839.784     -62.746     -47.242    -11980.927         0.000   NOSYM     0.754
AMS(1)

Corresponding output files
 1 : opt_niohdmf2_-1_2.12365861.out

Coordinates (Angs)
  84
opt_niohdmf2_-1_2.12365861.out       -11980.9176
O       2.427989    3.355169   -0.472448
C      -4.619845    1.442182    0.282922
H      -3.796683    2.126386    0.514886
H      -5.537526    1.835538    0.731176
H      -4.746758    1.418420   -0.805341
C      -4.085341    0.132994    2.340933
H      -3.909270   -0.859602    2.764079
H      -4.958018    0.575277    2.834277
H      -3.199611    0.746533    2.533362
C      -5.495536   -0.891388    0.519468
H      -5.688565   -0.917603   -0.558982
H      -6.404152   -0.546398    1.025916
H      -5.267790   -1.906150    0.856761
C      -4.325747    0.046112    0.829691
C      -2.370069   -1.489105    0.475969
C      -0.492167   -2.548715   -0.592877
C      -1.127616   -3.755381   -0.943270
H      -2.173133   -3.898596   -0.688627
C      -0.435835   -4.730894   -1.649007
H      -0.950993   -5.647333   -1.933926
C       0.900792   -4.541122   -2.018618
H       1.422500   -5.297527   -2.601643
C       1.564732   -3.377397   -1.647399
H       2.588396   -3.218683   -1.970348
C       0.897704   -2.392553   -0.903367
C       2.690807   -0.788858   -0.277720
C       3.832848   -1.606447   -0.261392
H       3.739941   -2.676781   -0.408497
C       5.080539   -1.057572    0.005657
H       5.956566   -1.703001    0.033561
C       5.203114    0.310961    0.261157
H       6.181530    0.737800    0.477134
C       4.083095    1.136141    0.276869
H       4.179237    2.189578    0.510639
C       2.806985    0.607347    0.026285
C       1.473730    2.623830   -0.143673
C      -0.270273    4.447589   -0.226270
C       0.040139    4.952521   -1.639457
H       1.117106    4.950223   -1.822799
H      -0.338294    5.973055   -1.765189
H      -0.443349    4.315327   -2.388210
C       0.355967    5.375666    0.820574
H       0.116917    5.023690    1.830528
H      -0.029600    6.395669    0.710097
H       1.443471    5.397000    0.708415
C      -1.787454    4.398854   -0.035686
H      -2.240787    3.708905   -0.756933
H      -2.220054    5.393976   -0.178503
H      -2.043583    4.055560    0.973845
N      -3.113186   -0.412438    0.143314
H      -2.652736    0.262915   -0.456046
N      -1.076492   -1.434567   -0.064764
N       1.387561   -1.184678   -0.468173
N       1.595098    1.260798    0.103545
N       0.192128    3.073098   -0.049810
H      -0.470844    2.411580    0.363819
O      -2.782879   -2.428010    1.177648
Ni      0.180583   -0.019296    0.232906
O      -0.895414    0.933793    1.385952
H      -0.273975    1.304288    2.033832
O      -2.179395    1.308749   -2.338437
C      -0.988146    1.176995   -2.645563
N      -0.451675    0.139474   -3.310671
C       0.957572    0.106331   -3.627485
C      -1.252306   -0.994761   -3.710892
H      -0.234975    1.939346   -2.379157
H       1.418924   -0.792781   -3.204510
H       1.452299    0.980933   -3.196416
H       1.111669    0.105487   -4.713914
H      -2.249438   -0.889831   -3.279724
H      -0.798047   -1.920586   -3.340847
H      -1.327901   -1.050030   -4.804533
O       1.376573    0.853552    3.748975
C       1.745167   -0.239362    3.298774
N       1.016773   -1.366967    3.270987
C       1.542366   -2.588205    2.705049
C      -0.345682   -1.407187    3.754816
```

```
H      -0.436555   -2.150033    4.557443
H      -1.024660   -1.674000    2.936912
H      -0.618784   -0.421394    4.132142
H       1.575864   -3.380097    3.463558
H       2.551858   -2.415833    2.323090
H       0.909071   -2.921803    1.874703
H       2.750350   -0.377808    2.857282

Frequencies (cm-1)
  100.00    100.00    100.00    100.00    100.00    100.00    100.00    100.00    100.00    100.00
  100.00    100.00    100.00    100.00    100.00    100.00    100.00    100.00    100.00    100.00
  105.74    107.40    116.25    119.39    126.07    127.92    143.81    157.70    172.52    179.22
  189.25    198.34    202.39    207.59    210.57    219.05    226.84    229.75    245.06    250.41
  251.29    256.24    259.24    261.88    270.32    278.70    302.59    313.70    317.67    318.88
  321.20    325.37    336.54    350.29    352.79    354.12    359.74    362.59    370.55    388.50
  400.25    400.62    412.75    414.80    437.99    447.89    460.80    461.41    466.63    469.86
  480.76    488.93    501.26    545.53    548.24    566.27    590.11    593.20    602.79    608.41
  650.32    652.38    676.27    717.94    722.41    724.84    733.64    735.76    739.54    756.99
  770.49    792.99    815.34    819.91    825.63    831.96    862.62    865.24    866.81    869.40
  886.33    890.00    899.25    906.39    907.84    909.15    912.21    912.98    915.31    929.49
  930.70    934.73    939.33    945.36    953.07    974.54    975.63   1015.85   1018.27   1023.45
 1026.28   1032.04   1044.38   1046.01   1046.31   1046.87   1054.42   1076.11   1079.51   1084.81
 1085.32   1118.02   1123.49   1133.88   1134.76   1135.61   1141.11   1187.36   1205.74   1209.37
 1212.06   1213.27   1215.48   1222.70   1238.86   1251.30   1254.11   1257.28   1272.23   1281.97
 1313.33   1328.36   1328.96   1331.43   1335.78   1336.28   1354.22   1361.38   1362.15   1363.98
 1373.66   1375.26   1378.66   1384.33   1388.04   1389.38   1391.46   1407.96   1408.70   1409.79
 1412.81   1416.45   1417.63   1425.53   1426.76   1427.81   1429.74   1432.78   1434.05   1436.47
 1436.78   1437.04   1440.35   1440.52   1443.30   1445.34   1446.14   1449.64   1463.67   1467.22
 1467.46   1488.38   1490.01   1521.74   1549.87   1551.58   1559.82   1569.57   1585.71   1621.33
 1641.11   1667.97   1670.97   2938.32   2971.40   2977.42   2977.75   2980.83   2982.09   2983.46
 2985.70   2985.86   2989.44   2993.16   2994.66   3062.94   3064.73   3068.41   3071.34   3079.50
 3082.67   3083.23   3086.19   3092.02   3096.18   3097.21   3102.05   3105.31   3105.50   3106.75
 3107.20   3109.48   3114.71   3122.61   3122.79   3122.86   3134.86   3140.11   3141.45   3168.15
 3171.91   3185.94   3194.76   3277.98   3514.59   3689.21
Note: any frequencies below 100 cm-1 (including spurious imaginary ones) are upscaled to 100 cm-1
      for the calculation of thermodynamic properties.
      (Averkiev, Truhlar, Catal. Sci. Technol. 2011, 1, 1526)

Thermodynamics

Note: this script does not take into account the spin entropy
For more info, see eq. 3 of Inorg. Chem. 2002, 41, 6928-6935
(M. Reiher), https://doi.org/10.1021/ic025891l

Temperature is now: 298.150

Reporting max. of 0 frequencies (set by $GETFREQSMAX env. variable)


Reading 1 outputfiles

               AMS(1)
---------------------
---------------------
(ZPVE)        429.227
(dH,0->T)      29.443
(-TS)         -74.813
(dGibbs)      383.856
=====================

Corresponding output files
 1 : frq_niohdmf2_-1_2.1327556.out
```

System niohdmf2_-2_1

```
Reading 1 outputfiles

       Pauli        Elstat        OrbInt       Disp.       Solv.         TOTAL          Erel   Symm.      <S2>
---------------------------------------------------------------------------------------------------------------------
-----
   44132.670     -9237.614    -46750.321     -61.773    -136.592    -12053.805         0.000   NOSYM     0.000
ADF(1)

Corresponding output files
 1 : opt_niohdmf2_-2_1.1327821.out

Coordinates (Angs)
  84
opt_niohdmf2_-2_1.1327821.out       -12053.8026
O       0.000000    0.000000    4.139294
C      -4.496175    0.958831   -1.969112
H      -4.476982    0.088646   -1.302820
H      -5.478603    1.011017   -2.450973
H      -4.345831    1.850437   -1.350561
C      -3.572713   -0.428914   -3.837068
H      -2.768413   -0.532212   -4.570928
H      -4.534539   -0.422864   -4.364324
H      -3.548545   -1.300239   -3.172593
C      -3.468872    2.083546   -3.940740
H      -3.366309    3.000031   -3.348789
H      -4.434613    2.110994   -4.459402
H      -2.664954    2.057926   -4.680007
C      -3.388908    0.859287   -3.022555
C      -0.892631    0.755896   -2.808715
C       1.421699    0.953114   -2.187979
C       1.877425    1.656407   -3.317004
H       1.161291    1.935499   -4.079756
C       3.221775    2.015260   -3.463362
H       3.531950    2.566448   -4.351268
C       4.150743    1.682256   -2.481417
H       5.197073    1.969694   -2.583220
C       3.731135    0.984402   -1.346420
H       4.447754    0.774275   -0.558579
C       2.388625    0.606404   -1.183034
C       2.440817   -0.702955    0.914681
C       3.728982   -1.268253    0.914584
H       4.374046   -1.146600    0.050312
C       4.171519   -2.038949    1.993526
H       5.168967   -2.477360    1.964159
C       3.338022   -2.253160    3.087948
H       3.676920   -2.854716    3.931041
C       2.047451   -1.706429    3.102844
H       1.391032   -1.886297    3.947739
C       1.574007   -0.939209    2.033124
C      -0.448079   -0.027760    2.965114
C      -2.753717    0.747476    3.608155
C      -2.361776    1.998401    4.405275
H      -1.457401    1.807860    4.988433
H      -3.170532    2.289840    5.085986
H      -2.165987    2.837284    3.727625
C      -3.068924   -0.406447    4.570444
H      -3.385553   -1.290694    4.005034
H      -3.875546   -0.130333    5.260367
H      -2.179116   -0.666218    5.150279
C      -3.998570    1.057657    2.770990
H      -3.804545    1.864977    2.055877
H      -4.827512    1.354929    3.422196
H      -4.308166    0.176590    2.196660
N      -2.137194    0.855157   -2.277411
H      -2.197336    0.514680   -1.297082
N       0.112936    0.631487   -1.853109
N       1.831353    0.000000   -0.090296
N       0.293155   -0.423930    1.865495
N      -1.712283    0.377237    2.661524
H      -1.989981    0.173089    1.681908
O      -0.686401    0.790086   -4.046602
Ni      0.000000    0.000000    0.000000
O      -1.899063   -0.260650    0.091217
H      -1.966325   -1.226856    0.069797
O      -2.960981    3.528608    0.106643
C      -1.810222    3.286971    0.490599
N      -0.677845    3.596282   -0.162489
C       0.615083    3.382997    0.446172
C      -0.702062    4.162079   -1.490020
H      -1.608218    2.764693    1.443885
H       1.258981    2.807381   -0.225196
H       0.495148    2.808123    1.368373
H       1.097941    4.343823    0.673648
H      -1.717529    4.091869   -1.882877
H      -0.026461    3.591258   -2.136986
H      -0.383111    5.213522   -1.480250
O      -1.356140   -3.444563    0.051960
C      -0.191294   -3.250677   -0.328696
N       0.195864   -3.069680   -1.602268
C       1.575776   -2.801193   -1.936023
C      -0.768908   -2.928490   -2.667871
```

```
H      -0.516681   -3.597282   -3.499395
H      -0.771525   -1.892927   -3.030069
H      -1.761097   -3.175753   -2.287426
H       1.935828   -3.529245   -2.673537
H       2.193371   -2.861361   -1.036193
H       1.673085   -1.789806   -2.347192
H       0.658433   -3.232434    0.378259

Frequencies (cm-1)
  100.00    100.00    100.00    100.00    100.00    100.00    100.00    100.00    100.00    100.00
  100.00    100.00    100.00    100.00    100.00    100.00    100.00    100.00    100.00    100.00
  100.00    100.00    104.89    111.01    117.07    119.95    147.33    153.99    171.78    179.14
  186.12    193.20    198.77    200.84    213.76    221.81    225.02    235.06    237.08    241.55
  247.22    255.53    260.21    263.47    264.12    303.96    305.75    309.43    313.08    320.96
  321.81    335.12    338.55    344.98    346.88    349.72    350.83    353.97    368.15    381.58
  403.89    404.32    411.98    416.15    433.29    445.58    456.02    460.10    461.94    465.07
  467.19    474.01    481.14    491.12    548.96    557.09    588.69    600.66    607.30    649.50
  652.14    670.05    696.06    704.98    720.04    728.83    738.83    745.15    753.57    769.27
  773.58    791.88    795.54    809.40    810.26    848.27    858.54    864.05    869.72    871.32
  873.03    877.63    890.42    896.54    899.73    901.87    905.47    907.81    909.66    913.91
  920.55    921.32    931.33    956.59    960.31    970.03    979.64   1010.11   1011.70   1022.44
 1024.17   1032.92   1044.76   1045.70   1046.84   1047.19   1053.08   1075.37   1077.37   1080.26
 1081.26   1104.21   1116.22   1128.01   1129.37   1130.18   1134.89   1189.50   1203.20   1204.59
 1211.25   1215.33   1215.86   1227.35   1233.73   1252.08   1256.84   1268.48   1278.18   1285.32
 1290.11   1307.61   1318.46   1320.82   1326.23   1330.74   1349.63   1356.23   1361.35   1370.05
 1373.08   1375.85   1381.43   1384.85   1387.00   1387.57   1392.00   1405.88   1407.19   1408.46
 1409.76   1411.65   1416.25   1422.04   1423.67   1424.14   1424.98   1426.03   1428.66   1430.97
 1432.96   1434.03   1435.85   1437.24   1438.51   1441.52   1441.73   1459.38   1463.14   1478.59
 1484.24   1487.39   1513.50   1544.89   1552.24   1559.58   1568.57   1576.49   1589.04   1599.72
 1616.23   1657.45   1676.20   2952.36   2958.44   2964.92   2967.01   2972.86   2977.49   2978.83
 2978.95   2981.67   2981.95   2984.97   2987.60   2988.55   3043.47   3061.66   3065.69   3069.22
 3071.91   3073.55   3075.23   3077.61   3079.80   3082.75   3084.60   3094.02   3095.25   3103.16
 3104.30   3104.90   3105.78   3106.78   3110.65   3112.43   3113.40   3123.96   3125.07   3126.02
 3128.11   3156.60   3173.38   3175.99   3193.79   3719.59
Note: any frequencies below 100 cm-1 (including spurious imaginary ones) are upscaled to 100 cm-1
      for the calculation of thermodynamic properties.
      (Averkiev, Truhlar, Catal. Sci. Technol. 2011, 1, 1526)

Thermodynamics

Note: this script does not take into account the spin entropy
For more info, see eq. 3 of Inorg. Chem. 2002, 41, 6928-6935
(M. Reiher), https://doi.org/10.1021/ic025891l

Temperature is now: 298.150

Reporting max. of 0 frequencies (set by $GETFREQSMAX env. variable)


Reading 1 outputfiles

            ScnFrq(1)
---------------------
---------------------
(ZPVE)        427.325
(dH,0->T)      29.586
(-TS)         -75.255
(dGibbs)      381.656
=====================

Corresponding output files
 1 : frq_niohdmf2_-2_1.1328094.out
```

System niohdmf2_-2_3

```
Reading 1 outputfiles

       Pauli        Elstat        OrbInt       Disp.       Solv.         TOTAL          Erel    Symm.      <S2>
-----------------------------------------------------------------------------------------------------------------------
-----
       0.000         0.000         0.000       0.000       0.000    -12040.454         0.000    NOSYM     2.009
Quild(1)

Corresponding output files
 1 : opt_niohdmf2_-2_3.1327822.out

Coordinates (Angs)
  84
opt_niohdmf2_-2_3.1327822.out       -12040.4392
O        0.000000    0.000000    4.248922
C        3.357803   -3.079356   -1.428276
H        3.442255   -2.358927   -0.606482
H        4.363548   -3.386573   -1.734212
H        2.816756   -3.956635   -1.055061
C        3.347857   -1.177728   -3.045809
H        2.837106   -0.716828   -3.896652
H        4.378966   -1.409504   -3.338845
H        3.350597   -0.464258   -2.214686
C        2.522333   -3.452477   -3.755803
H        2.015632   -4.365873   -3.422922
H        3.525535   -3.719686   -4.108836
H        1.954351   -3.024302   -4.585578
C        2.611001   -2.448844   -2.604756
C        0.347496   -1.337031   -2.738727
C       -1.780481   -0.350346   -2.215978
C       -2.488912   -0.772995   -3.355019
H       -1.966651   -1.380779   -4.087172
C       -3.833951   -0.450590   -3.548686
H       -4.348265   -0.801011   -4.443884
C       -4.514849    0.302632   -2.594653
H       -5.570362    0.541084   -2.724988
C       -3.839177    0.754592   -1.459360
H       -4.392831    1.305113   -0.705268
C       -2.477879    0.472079   -1.246604
C       -2.022936    1.656033    0.877129
C       -2.910289    2.746586    0.835455
H       -3.450930    2.957752   -0.082351
C       -3.078095    3.596864    1.929671
H       -3.771907    4.434460    1.861838
C       -2.351442    3.370987    3.097929
H       -2.481188    4.024203    3.961070
C       -1.438634    2.316444    3.164342
H       -0.856540    2.155196    4.066018
C       -1.223706    1.455806    2.072804
C        0.371433   -0.072393    3.050504
C        2.383731   -1.535515    3.547229
C        1.653012   -2.645653    4.313482
H        0.854689   -2.222020    4.927967
H        2.353714   -3.182519    4.963713
H        1.217974   -3.364243    3.610814
C        3.084911   -0.589932    4.531109
H        3.632310    0.185687    3.983036
H        3.797123   -1.139092    5.158520
H        2.346776   -0.103878    5.175115
C        3.419354   -2.176231    2.620755
H        2.924909   -2.840535    1.902974
H        4.141664   -2.757749    3.203290
H        3.965115   -1.410688    2.056578
N        1.272095   -2.132859   -2.098112
H        1.258421   -1.996696   -1.095234
N       -0.510061   -0.732316   -1.860754
N       -1.734786    0.787498   -0.142866
N       -0.262564    0.484191    1.970816
N        1.472361   -0.801328    2.679959
H        1.735754   -0.719346    1.694396
O        0.338191   -1.227857   -3.989186
Ni       0.000000    0.000000    0.000000
O        1.922234    0.000000   -0.110718
H        2.186988    0.774815    0.406078
O        0.556936   -4.010025    0.894614
C       -0.411632   -3.299894    1.187955
N       -1.548971   -3.180243    0.480939
C       -2.641904   -2.365865    0.960807
C       -1.716592   -3.833625   -0.795939
H       -0.418450   -2.670639    2.095973
H       -2.928145   -1.633915    0.199530
H       -2.324422   -1.815411    1.850605
H       -3.511392   -2.990240    1.207770
H       -0.755635   -4.246884   -1.107888
H       -2.042814   -3.096588   -1.538002
H       -2.464441   -4.635775   -0.732640
O        2.111040    3.267484    0.465817
C        1.044752    3.343981   -0.160827
N        0.868441    3.042832   -1.457056
C       -0.425967    3.162987   -2.087276
C        1.924441    2.468286   -2.258852
```

```
H       2.053976    3.047806   -3.181354
H       1.673408    1.429316   -2.502916
H       2.851839    2.471952   -1.685372
H      -0.377575    3.870238   -2.925412
H      -1.160557    3.515760   -1.359595
H      -0.754772    2.186429   -2.459304
H       0.109493    3.684635    0.321866

Frequencies (cm-1)
  100.00    100.00    100.00    100.00    100.00    100.00    100.00    100.00    100.00    100.00
  100.00    100.00    100.00    100.00    100.00    100.00    100.00    100.00    100.00    100.00
  100.00    100.00    108.01    112.13    117.92    119.19    140.71    147.79    158.16    163.53
  173.66    179.56    190.43    194.76    200.24    202.92    209.32    213.25    227.23    238.99
  244.99    247.59    252.62    262.22    268.04    269.95    277.87    289.42    297.34    307.33
  316.69    316.97    317.91    330.62    336.43    342.85    345.98    350.45    352.66    364.25
  404.24    407.04    409.39    410.79    421.34    436.37    447.04    456.75    458.82    463.84
  472.64    478.03    485.03    496.86    557.71    561.05    563.48    582.18    596.81    601.33
  649.77    652.19    658.99    695.85    702.27    709.67    728.02    733.02    739.21    744.52
  753.35    771.59    788.97    801.42    806.03    808.12    818.32    833.61    837.78    852.87
  868.51    868.87    870.91    885.54    895.35    902.22    903.59    909.45    909.79    913.45
  920.07    924.51    925.98    938.30    942.25    964.04    969.09   1012.08   1014.07   1025.38
 1026.50   1033.55   1044.57   1046.30   1047.74   1048.86   1053.31   1076.96   1080.00   1082.58
 1083.24   1106.60   1116.34   1129.22   1130.72   1132.83   1133.49   1183.89   1198.90   1205.79
 1208.43   1212.09   1216.79   1224.37   1233.51   1252.02   1255.41   1260.19   1268.02   1273.57
 1318.62   1321.04   1324.94   1327.03   1328.45   1333.34   1340.33   1353.91   1358.74   1361.56
 1365.37   1373.84   1375.33   1384.16   1385.67   1386.93   1388.87   1404.39   1408.84   1409.56
 1412.71   1414.52   1415.52   1418.56   1424.46   1424.55   1426.27   1429.85   1431.68   1432.00
 1433.15   1433.90   1434.91   1436.93   1439.44   1441.18   1446.69   1462.44   1468.88   1470.59
 1485.48   1486.33   1486.62   1495.91   1536.61   1544.70   1549.22   1551.11   1567.93   1587.11
 1596.97   1666.72   1676.61   2945.57   2966.94   2970.61   2975.59   2975.93   2978.33   2979.39
 2981.28   2982.14   2983.11   2986.99   2990.79   3065.31   3066.14   3072.04   3072.11   3075.28
 3076.71   3082.90   3083.16   3085.31   3088.10   3092.78   3095.09   3099.77   3102.30   3103.90
 3104.28   3109.64   3109.72   3112.42   3113.43   3122.00   3123.43   3123.54   3130.51   3152.23
 3167.92   3169.57   3171.94   3296.32   3536.97   3730.43
Note: any frequencies below 100 cm-1 (including spurious imaginary ones) are upscaled to 100 cm-1
      for the calculation of thermodynamic properties.
      (Averkiev, Truhlar, Catal. Sci. Technol. 2011, 1, 1526)

Thermodynamics

Note: this script does not take into account the spin entropy
For more info, see eq. 3 of Inorg. Chem. 2002, 41, 6928-6935
(M. Reiher), https://doi.org/10.1021/ic025891l

Temperature is now: 298.150

Reporting max. of 0 frequencies (set by $GETFREQSMAX env. variable)


Reading 1 outputfiles

            ScnFrq(1)
---------------------
---------------------
(ZPVE)        426.796
(dH,0->T)      30.062
(-TS)         -76.270
(dGibbs)      380.588
=====================

Corresponding output files
 1 : frq_niohdmf2_-2_3.1328095.out
```

System Ni-OH_0_1

```
Reading 1 outputfiles

       Pauli         Elstat        OrbInt       Disp.       Solv.         TOTAL          Erel   Symm.      <S2>
-----------------------------------------------------------------------------------------------------------------
-----
   33149.867     -7014.970    -34779.054     -33.976     -12.263     -8690.563         0.000   NOSYM    -0.000
ADF(1)

Corresponding output files
 1 : opt_Ni-OH_0_1.1243052.out

Coordinates (Angs)
  60
opt_Ni-OH_0_1.1243052.out        -8690.5636
Ni      -0.048261    0.252601    0.535459
O        0.148919   -1.290333    1.482431
H       -0.643479   -1.426337    2.024301
C       -1.640495   -4.119535   -0.719711
H       -0.855208   -3.758454   -1.394193
H       -2.009189   -5.072965   -1.107997
H       -1.195965   -4.303873    0.265427
C       -3.857227   -3.634597    0.336226
H       -3.426559   -3.810415    1.328093
H       -4.265059   -4.580441   -0.035642
H       -4.675051   -2.916161    0.432743
C       -3.375624   -2.873731   -2.018790
H       -4.185958   -2.141738   -1.979997
H       -3.772139   -3.812079   -2.420396
H       -2.602641   -2.504410   -2.701426
C       -2.786856   -3.112778   -0.626028
C       -2.818134   -0.702819    0.130937
C       -2.354769    1.595096    0.742913
C       -3.631071    2.064321    1.119140
H       -4.432276    1.356266    1.287386
C       -3.825090    3.414721    1.325921
H       -4.802816    3.762984    1.653472
C       -2.777996    4.347324    1.180222
H       -2.948867    5.394662    1.416737
C       -1.530962    3.930980    0.776337
H       -0.710671    4.637754    0.721300
C       -1.315298    2.564077    0.505548
C        0.942205    2.552298   -0.483929
C        1.040870    3.842455   -1.051737
H        0.167232    4.480888   -1.111823
C        2.228009    4.244880   -1.611615
H        2.302714    5.222557   -2.081305
C        3.340063    3.373371   -1.618467
H        4.268626    3.703727   -2.080301
C        3.273592    2.105588   -1.091169
H        4.131471    1.447533   -1.135787
C        2.063560    1.642272   -0.510959
C        2.846156   -0.534844    0.157437
C        3.212420   -3.008479    0.371952
C        3.651594   -2.952351    1.838545
H        4.317768   -2.103928    2.015123
H        4.183438   -3.871893    2.104778
H        2.778457   -2.853612    2.492562
C        4.419613   -3.135684   -0.557305
H        4.095127   -3.177384   -1.602700
H        4.966423   -4.056278   -0.328574
H        5.096963   -2.287278   -0.436229
C        2.273862   -4.195803    0.162309
H        1.401642   -4.126735    0.823007
H        2.796159   -5.129889    0.387926
H        1.923013   -4.240394   -0.874926
N       -2.185670   -1.877902   -0.109244
H       -1.213320   -1.932431    0.174050
N       -1.948549    0.304988    0.586448
N       -0.163971    1.985279    0.054055
N        1.801950    0.413385   -0.011363
N        2.425192   -1.813061    0.019020
H        1.424294   -1.934527    0.120914
O       -4.022592   -0.512450   -0.067679
O        4.013822   -0.206021    0.382584

Frequencies (cm-1)
   100.00    100.00    100.00    100.00    100.00    100.00    100.00    100.00    100.00    100.00
   100.00    100.00    135.60    146.57    167.63    179.22    196.87    198.03    201.85    208.66
   217.94    229.96    242.78    245.92    247.98    269.62    276.04    279.20    289.51    309.55
   313.79    331.94    334.74    347.84    350.15    355.38    366.55    383.67    394.27    412.62
   415.79    433.52    446.42    452.51    461.53    465.61    466.79    472.72    497.90    510.46
   512.68    561.17    571.55    584.21    586.95    602.98    674.74    683.99    722.60    728.68
   732.60    737.09    746.96    748.27    757.55    773.68    802.01    817.53    830.44    840.52
   852.13    862.24    868.14    891.82    905.28    910.13    911.24    916.10    917.59    930.29
   931.10    932.91    939.37    943.65    949.15    967.45    970.02   1008.62   1016.49   1018.24
  1021.07   1022.01   1034.56   1037.00   1044.37   1126.83   1130.17   1133.82   1146.16   1192.18
  1198.35   1204.74   1208.72   1212.98   1215.84   1217.10   1232.99   1237.99   1257.41   1287.33
  1309.86   1332.95   1333.65   1337.56   1338.47   1342.62   1362.05   1362.54   1364.11   1381.97
  1391.31   1409.90   1410.88   1418.91   1423.93   1425.60   1425.96   1428.88   1430.71   1434.02
  1441.19   1442.39   1456.42   1458.22   1463.52   1464.31   1468.64   1515.81   1525.10   1539.86
  1548.20   1586.74   1599.05   1656.76   1667.48   2986.61   2989.42   2994.48   2994.94   2999.55
```

```
 3000.99   3081.80   3085.52   3092.69   3093.95   3094.89   3095.61   3106.74   3108.23   3111.77
 3113.67   3116.64   3120.04   3144.14   3144.31   3160.82   3161.81   3184.79   3198.76   3211.05
 3212.81   3458.99   3511.16   3722.71
Note: any frequencies below 100 cm-1 (including spurious imaginary ones) are upscaled to 100 cm-1
      for the calculation of thermodynamic properties.
      (Averkiev, Truhlar, Catal. Sci. Technol. 2011, 1, 1526)

Thermodynamics

Note: this script does not take into account the spin entropy
For more info, see eq. 3 of Inorg. Chem. 2002, 41, 6928-6935
(M. Reiher), https://doi.org/10.1021/ic025891l

Temperature is now: 298.150

Reporting max. of 0 frequencies (set by $GETFREQSMAX env. variable)


Reading 1 outputfiles

            ScnFrq(1)
---------------------
---------------------
(ZPVE)        301.747
(dH,0->T)      20.167
(-TS)         -55.896
(dGibbs)      266.018
=====================

Corresponding output files
 1 : frq_Ni-OH_0_1.1327865.out
```

System Ni-OH_0_3

```
Reading 1 outputfiles

       Pauli        Elstat        OrbInt       Disp.       Solv.         TOTAL          Erel   Symm.      <S2>
----------------------------------------------------------------------------------------------------------------------
-----
   33138.998     -7012.107    -34764.203     -34.572     -13.964     -8686.018         0.000   NOSYM     2.007
ADF(1)

Corresponding output files
 1 : opt_Ni-OH_0_3.1243053.out

Coordinates (Angs)
  60
opt_Ni-OH_0_3.1243053.out        -8686.0282
Ni     -0.026558    0.377493    0.800255
O       0.219207   -1.148756    1.819474
H      -0.358520   -1.034236    2.590994
C      -1.639842   -4.263553    0.694174
H      -0.607353   -4.099297    0.366581
H      -1.933033   -5.273517    0.394068
H      -1.664350   -4.208605    1.788175
C      -4.009355   -3.487148    0.578950
H      -4.045436   -3.402758    1.670414
H      -4.328837   -4.496827    0.300286
H      -4.709975   -2.767291    0.150175
C      -2.528545   -3.337221   -1.455393
H      -3.175123   -2.587673   -1.919562
H      -2.858719   -4.329584   -1.780158
H      -1.504123   -3.182244   -1.812405
C      -2.586588   -3.237601    0.072731
C      -2.656077   -0.733362    0.211891
C      -2.372071    1.661843    0.565686
C      -3.707788    2.048843    0.740639
H      -4.464786    1.296048    0.926962
C      -4.042800    3.393884    0.702534
H      -5.078697    3.690647    0.852007
C      -3.061351    4.372514    0.503342
H      -3.335116    5.425219    0.509183
C      -1.733968    4.017914    0.322610
H      -0.982649    4.791750    0.223070
C      -1.373755    2.661277    0.333811
C       1.043178    2.650962   -0.269261
C       1.266418    3.891527   -0.886487
H       0.447437    4.570885   -1.087774
C       2.551128    4.238199   -1.276016
H       2.720501    5.204756   -1.745138
C       3.629861    3.360742   -1.083836
H       4.628829    3.658598   -1.394374
C       3.433206    2.110612   -0.526652
H       4.262267    1.421423   -0.397098
C       2.137525    1.730038   -0.133202
C       2.686390   -0.568055    0.439931
C       2.959806   -2.947415   -0.349491
C       2.772326   -3.578898    1.031685
H       3.330069   -3.025841    1.792149
H       3.132871   -4.612422    1.019492
H       1.714657   -3.572456    1.313796
C       4.440010   -2.878237   -0.727306
H       4.564376   -2.419842   -1.714204
H       4.858097   -3.889447   -0.761006
H       5.003931   -2.293292    0.003432
C       2.194762   -3.744977   -1.402859
H       1.126798   -3.798477   -1.161551
H       2.578646   -4.767773   -1.447133
H       2.305116   -3.294108   -2.395471
N      -2.098278   -1.923533    0.508143
H      -1.188768   -1.888728    0.983583
N      -1.870139    0.375681    0.639964
N      -0.124984    2.115752    0.191343
N       1.754699    0.508828    0.322938
N       2.369467   -1.595563   -0.360612
H       1.522388   -1.475815   -0.899784
O      -3.715968   -0.579018   -0.400523
O       3.642980   -0.503467    1.208845

Frequencies (cm-1)
  100.00    100.00    100.00    100.00    100.00    100.00    100.00    100.00    100.00    100.00
  102.68    112.77    126.37    140.19    159.01    174.74    183.58    189.71    195.28    206.19
  212.95    219.46    239.91    241.98    242.12    250.43    261.32    264.58    284.85    315.75
  319.96    327.53    334.34    346.73    353.15    359.77    379.17    385.50    392.28    406.66
  415.80    435.69    447.43    457.59    464.68    466.96    470.65    482.19    492.53    516.18
  520.41    530.60    547.34    581.94    592.46    604.27    670.09    712.24    718.76    721.56
  727.76    730.90    737.09    757.44    770.27    798.05    815.47    824.38    830.45    835.49
  844.40    857.09    878.66    892.46    905.95    909.45    912.69    915.19    916.25    918.16
  919.41    922.32    930.64    933.08    946.90    955.26    957.84   1001.06   1015.92   1016.18
 1018.32   1027.05   1033.24   1038.49   1045.50   1119.85   1125.64   1141.56   1147.16   1192.92
 1201.74   1205.22   1209.03   1212.45   1216.39   1217.28   1237.35   1244.76   1260.31   1287.00
 1291.40   1330.95   1333.40   1337.73   1338.60   1341.14   1361.79   1364.01   1366.20   1372.68
 1380.35   1410.03   1410.57   1423.11   1425.06   1426.37   1427.92   1430.53   1432.11   1436.07
 1440.33   1443.02   1447.62   1453.95   1458.01   1459.74   1460.66   1491.96   1548.61   1552.53
 1557.08   1565.68   1579.02   1667.29   1691.97   2991.66   2992.75   2995.65   2998.85   3000.92
```

```
 3003.71   3086.62   3089.59   3092.72   3094.78   3098.67   3104.56   3109.19   3110.34   3111.73
 3112.21   3117.15   3120.53   3143.98   3145.39   3158.68   3159.34   3177.36   3192.09   3195.47
 3211.16   3220.99   3563.55   3718.05
Note: any frequencies below 100 cm-1 (including spurious imaginary ones) are upscaled to 100 cm-1
      for the calculation of thermodynamic properties.
      (Averkiev, Truhlar, Catal. Sci. Technol. 2011, 1, 1526)

Thermodynamics

Note: this script does not take into account the spin entropy
For more info, see eq. 3 of Inorg. Chem. 2002, 41, 6928-6935
(M. Reiher), https://doi.org/10.1021/ic025891l

Temperature is now: 298.150

Reporting max. of 0 frequencies (set by $GETFREQSMAX env. variable)


Reading 1 outputfiles

            ScnFrq(1)
---------------------
---------------------
(ZPVE)        301.227
(dH,0->T)      20.270
(-TS)         -56.125
(dGibbs)      265.372
=====================

Corresponding output files
 1 : frq_Ni-OH_0_3.1327864.out
```

System Ni-OH_-1_2

```
Reading 1 outputfiles

       Pauli        Elstat        OrbInt       Disp.       Solv.         TOTAL          Erel    Symm.      <S2>
-----------------------------------------------------------------------------------------------------------------
-----
   32989.491     -6981.552    -34713.711     -33.839     -47.231     -8786.990         0.000    NOSYM     0.754
ADF(1)

Corresponding output files
 1 : opt_Ni-OH_-1_2.1243051.out

Coordinates (Angs)
  60
opt_Ni-OH_-1_2.1243051.out        -8786.9759
Ni      -0.031290    0.231832    0.541893
O        0.133922   -1.341914    1.501773
H       -0.688394   -1.466705    1.997087
C       -1.640884   -4.103220   -0.824051
H       -0.855106   -3.745714   -1.499943
H       -2.024180   -5.049860   -1.215928
H       -1.189403   -4.298510    0.155862
C       -3.843437   -3.601515    0.249465
H       -3.406268   -3.796167    1.235346
H       -4.273005   -4.536049   -0.127862
H       -4.646028   -2.868179    0.362903
C       -3.369886   -2.833469   -2.102327
H       -4.169912   -2.090914   -2.052723
H       -3.779944   -3.765317   -2.506839
H       -2.597667   -2.468693   -2.788626
C       -2.772464   -3.080371   -0.714186
C       -2.774311   -0.679948    0.088806
C       -2.345762    1.593310    0.794261
C       -3.599033    2.036125    1.235361
H       -4.377896    1.309181    1.436001
C       -3.825332    3.393424    1.444581
H       -4.798338    3.722960    1.805013
C       -2.808141    4.328463    1.233642
H       -2.980970    5.382314    1.443301
C       -1.562476    3.917914    0.777603
H       -0.767289    4.646519    0.663863
C       -1.325721    2.556835    0.522946
C        0.953544    2.547103   -0.479880
C        1.025807    3.818090   -1.074102
H        0.142881    4.445630   -1.124339
C        2.208453    4.252391   -1.653981
H        2.254983    5.235113   -2.119087
C        3.325349    3.412775   -1.652984
H        4.254844    3.746746   -2.111917
C        3.269679    2.140699   -1.096699
H        4.139711    1.498736   -1.128467
C        2.079973    1.660129   -0.510971
C        2.835205   -0.561903    0.064152
C        3.199740   -3.017913    0.436980
C        3.782172   -2.853875    1.845207
H        4.462061   -1.998730    1.885995
H        4.337563   -3.752752    2.135267
H        2.976880   -2.695044    2.571277
C        4.312269   -3.246942   -0.587046
H        3.888383   -3.357894   -1.591504
H        4.861692   -4.162543   -0.342199
H        5.010958   -2.407801   -0.595684
C        2.244969   -4.212258    0.417888
H        1.433218   -4.077161    1.142189
H        2.783694   -5.129221    0.675309
H        1.801167   -4.340642   -0.575967
N       -2.152210   -1.859535   -0.196211
H       -1.196757   -1.945784    0.134069
N       -1.927453    0.283406    0.626648
N       -0.154996    1.972338    0.087143
N        1.816416    0.393543   -0.024412
N        2.382560   -1.851695    0.061467
H        1.413585   -1.939097    0.361230
O       -3.978128   -0.478967   -0.147169
O        4.044397   -0.280874    0.096590

Frequencies (cm-1)
  100.00    100.00    100.00    100.00    100.00    100.00    100.00    100.00    100.00    100.00
  100.00    100.00    141.14    145.44    170.18    184.10    196.01    199.34    201.30    212.05
  221.77    231.12    240.31    244.81    250.26    261.43    271.45    276.34    300.75    311.60
  314.10    333.35    343.98    345.69    352.17    355.34    366.95    374.32    383.44    411.24
  416.57    434.57    447.80    457.50    459.21    463.49    470.19    473.45    485.81    536.71
  541.53    542.92    590.13    596.28    605.80    612.05    676.06    676.42    718.78    726.05
  730.80    737.67    740.41    746.20    757.28    772.18    802.88    818.78    824.05    834.22
  840.92    857.34    865.94    890.19    903.88    906.73    906.85    907.47    913.97    915.09
  916.27    927.20    929.23    944.39    947.61    949.01    953.22   1014.62   1016.21   1019.29
 1024.58   1032.24   1043.79   1045.45   1051.83   1115.15   1122.01   1132.81   1139.03   1185.95
 1197.31   1204.17   1209.65   1212.24   1214.40   1219.90   1236.15   1251.56   1262.11   1278.75
 1288.48   1309.81   1328.53   1329.98   1334.70   1335.89   1354.16   1358.55   1359.91   1361.41
 1374.29   1408.44   1408.80   1423.12   1424.69   1424.90   1427.12   1429.41   1433.01   1433.77
 1435.86   1440.48   1440.68   1444.37   1457.87   1461.31   1463.32   1518.40   1539.38   1551.22
 1561.29   1568.80   1583.02   1625.19   1630.70   2985.25   2987.05   2989.99   2990.33   2996.12
```

```
 2996.32   3080.67   3083.72   3087.20   3087.41   3088.30   3089.92   3101.61   3104.23   3108.24
 3110.50   3114.86   3120.01   3129.18   3132.32   3147.92   3148.83   3175.87   3189.86   3191.72
 3211.57   3412.60   3457.83   3739.29
Note: any frequencies below 100 cm-1 (including spurious imaginary ones) are upscaled to 100 cm-1
      for the calculation of thermodynamic properties.
      (Averkiev, Truhlar, Catal. Sci. Technol. 2011, 1, 1526)

Thermodynamics

Note: this script does not take into account the spin entropy
For more info, see eq. 3 of Inorg. Chem. 2002, 41, 6928-6935
(M. Reiher), https://doi.org/10.1021/ic025891l

Temperature is now: 298.150

Reporting max. of 0 frequencies (set by $GETFREQSMAX env. variable)


Reading 1 outputfiles

            ScnFrq(1)
--------------------
--------------------
(ZPVE)        301.025
(dH,0->T)      20.144
(-TS)         -55.831
(dGibbs)      265.338
====================

Corresponding output files
 1 : frq_Ni-OH_-1_2.1327557.out
```

System Ni-OH_-2_1

```
Reading 1 outputfiles

       Pauli        Elstat        OrbInt       Disp.       Solv.         TOTAL          Erel   Symm.      <S2>
---------------------------------------------------------------------------------------------------------------------
-----
   32788.467     -6935.779    -34527.310     -35.071    -153.489     -8863.300         0.000   NOSYM     0.000
ADF(1)

Corresponding output files
 1 : opt_Ni-OH_-2_1.1243054.out

Coordinates (Angs)
  60
opt_Ni-OH_-2_1.1243054.out        -8863.3156
Ni      -0.095335    0.153716    0.623997
O        0.059935   -1.615651    1.349927
H        0.489218   -1.493001    2.207567
C       -1.913011   -4.222786   -0.015863
H       -0.853433   -3.989486    0.130401
H       -1.991069   -5.013478   -0.769534
H       -2.315604   -4.606418    0.928984
C       -4.152886   -3.352400   -0.675020
H       -4.591582   -3.710650    0.263438
H       -4.236765   -4.151089   -1.421662
H       -4.725630   -2.486472   -1.013463
C       -2.075095   -2.462867   -1.778034
H       -2.611014   -1.572937   -2.124591
H       -2.127081   -3.224535   -2.564617
H       -1.023998   -2.190675   -1.623186
C       -2.684468   -2.982434   -0.468526
C       -2.887761   -0.660518    0.418742
C       -2.400482    1.597606    1.001840
C       -3.629041    2.050717    1.480049
H       -4.380377    1.322202    1.774762
C       -3.895954    3.419288    1.593080
H       -4.860092    3.755862    1.971131
C       -2.915304    4.341242    1.233762
H       -3.100844    5.409715    1.339772
C       -1.681201    3.911389    0.742882
H       -0.925494    4.653487    0.510735
C       -1.404508    2.536491    0.600181
C        0.819816    2.477506   -0.497442
C        0.870299    3.722207   -1.149238
H       -0.002006    4.365921   -1.159152
C        2.013809    4.130711   -1.838820
H        2.025565    5.101476   -2.333254
C        3.120500    3.290364   -1.894370
H        4.020802    3.596463   -2.426293
C        3.084409    2.035462   -1.277418
H        3.945783    1.382554   -1.343460
C        1.945476    1.590269   -0.584744
C        2.748974   -0.605416   -0.011230
C        3.161405   -3.059753    0.278598
C        3.745071   -2.933176    1.691818
H        4.395801   -2.057471    1.762576
H        4.329759   -3.823876    1.949524
H        2.939059   -2.828886    2.427558
C        4.281316   -3.237965   -0.748729
H        3.859251   -3.333245   -1.755832
H        4.854667   -4.145729   -0.528363
H        4.955723   -2.379139   -0.736529
C        2.238844   -4.278962    0.224863
H        1.427531   -4.185518    0.955588
H        2.800927   -5.191676    0.447500
H        1.791084   -4.381295   -0.770469
N       -2.517580   -1.982033    0.606229
H       -1.567180   -2.020550    1.007412
N       -1.997702    0.258976    0.930110
N       -0.242786    1.931632    0.180267
N        1.716335    0.327740   -0.039191
N        2.318783   -1.905668   -0.064013
H        1.354075   -1.986645    0.302045
O       -3.956727   -0.356428   -0.154327
O        3.961187   -0.305770    0.018047

Frequencies (cm-1)
   100.00    100.00    100.00    100.00    100.00    100.00    100.00    100.00    100.00    100.00
   102.27    128.27    143.29    154.39    174.62    177.55    191.33    194.98    199.80    214.50
   223.35    230.19    231.23    244.42    247.38    252.75    264.92    275.28    299.28    302.27
   310.72    319.95    332.39    339.66    344.16    353.28    360.32    367.69    372.96    410.14
   418.12    434.97    441.87    452.34    457.63    463.54    467.17    483.77    486.67    493.30
   548.74    558.04    590.35    601.06    608.58    669.75    695.64    702.90    711.98    716.84
   721.42    725.57    734.72    752.83    769.00    773.38    793.37    799.20    813.75    844.88
   855.41    868.65    873.76    882.82    888.58    901.23    902.62    905.00    912.04    912.34
   914.99    917.78    922.42    924.06    925.87    969.88    982.35   1013.25   1016.32   1018.21
  1021.04   1032.22   1046.25   1047.49   1053.27   1103.59   1114.32   1129.65   1137.85   1185.18
  1196.43   1203.37   1204.68   1213.27   1214.46   1224.71   1231.63   1249.76   1256.77   1273.34
  1280.69   1296.27   1324.16   1328.32   1330.44   1333.04   1340.27   1355.73   1358.50   1374.59
  1382.86   1406.71   1407.10   1421.10   1421.82   1422.25   1425.41   1426.49   1430.84   1432.40
  1434.69   1437.34   1438.23   1458.57   1461.24   1473.79   1502.66   1537.05   1548.11   1556.94
  1561.60   1568.15   1578.11   1607.68   1615.60   2979.00   2983.59   2985.54   2987.23   2992.22
```

```
 2992.81   3078.45   3078.80   3080.68   3082.41   3084.71   3088.29   3090.24   3095.39   3101.54
 3102.54   3105.98   3116.29   3117.34   3117.49   3119.03   3134.65   3136.36   3154.63   3165.19
 3172.54   3192.35   3195.73   3754.15
Note: any frequencies below 100 cm-1 (including spurious imaginary ones) are upscaled to 100 cm-1
      for the calculation of thermodynamic properties.
      (Averkiev, Truhlar, Catal. Sci. Technol. 2011, 1, 1526)

Thermodynamics

Note: this script does not take into account the spin entropy
For more info, see eq. 3 of Inorg. Chem. 2002, 41, 6928-6935
(M. Reiher), https://doi.org/10.1021/ic025891l

Temperature is now: 298.150

Reporting max. of 0 frequencies (set by $GETFREQSMAX env. variable)


Reading 1 outputfiles

            ScnFrq(1)
---------------------
---------------------
(ZPVE)        299.789
(dH,0->T)      20.182
(-TS)         -55.805
(dGibbs)      264.167
=====================

Corresponding output files
 1 : frq_Ni-OH_-2_1.1327558.out
```

System Ni-OH_-2_3

```
Reading 1 outputfiles

       Pauli        Elstat        OrbInt       Disp.       Solv.         TOTAL          Erel    Symm.      <S2>
----------------------------------------------------------------------------------------------------------------------
-----
   32644.617     -6863.275    -34444.416     -35.172    -156.926     -8855.286         0.000    NOSYM     2.009
ADF(1)

Corresponding output files
 1 : opt_Ni-OH_-2_3.1243055.out

Coordinates (Angs)
  60
opt_Ni-OH_-2_3.1243055.out        -8855.2831
Ni      0.015250    0.231731    0.821557
O       0.157467   -1.117585    2.190906
H      -0.707425   -1.323590    2.569184
C      -1.666020   -4.332177    0.144652
H      -0.600018   -4.084671    0.102004
H      -1.810025   -5.284098   -0.375884
H      -1.944829   -4.466910    1.195869
C      -3.987683   -3.646771   -0.433603
H      -4.293104   -3.776299    0.610828
H      -4.142829   -4.595744   -0.959155
H      -4.619565   -2.880541   -0.887050
C      -2.082261   -3.064198   -1.969130
H      -2.665637   -2.272239   -2.447447
H      -2.230203   -3.992746   -2.532108
H      -1.021114   -2.795050   -2.026065
C      -2.514011   -3.240729   -0.507446
C      -2.738488   -0.758034   -0.094040
C      -2.421366    1.566038    0.404912
C      -3.756715    1.982812    0.513363
H      -4.537125    1.227808    0.514947
C      -4.091079    3.331291    0.638811
H      -5.137059    3.621766    0.730443
C      -3.086975    4.297568    0.670544
H      -3.336745    5.349917    0.800777
C      -1.750971    3.919010    0.544802
H      -0.985732    4.684018    0.618880
C      -1.382570    2.570853    0.373388
C       1.056372    2.678341   -0.093709
C       1.217042    3.988150   -0.589121
H       0.347047    4.614775   -0.749758
C       2.471068    4.492114   -0.931802
H       2.554298    5.506815   -1.319674
C       3.605032    3.698591   -0.772190
H       4.592650    4.092825   -1.009509
C       3.474301    2.382178   -0.329064
H       4.355594    1.754354   -0.244692
C       2.222241    1.827557   -0.016434
C       2.847817   -0.485121    0.041051
C       2.944306   -2.983984    0.277405
C       4.447915   -3.221119    0.124415
H       4.869507   -2.540567   -0.618046
H       4.635921   -4.254339   -0.189925
H       4.958525   -3.052774    1.079359
C       2.233469   -3.209644   -1.064093
H       1.159283   -3.019449   -0.963018
H       2.368275   -4.240763   -1.409989
H       2.629181   -2.532823   -1.826708
C       2.388909   -3.954819    1.319684
H       2.896785   -3.820270    2.281598
H       2.537434   -4.988807    0.992524
H       1.316981   -3.791421    1.477002
N      -2.271889   -2.015121    0.268066
H      -1.353565   -1.980508    0.702796
N      -1.980080    0.257495    0.410438
N      -0.115619    2.050608    0.247163
N       1.995038    0.527370    0.399690
N       2.668240   -1.633837    0.795136
H       1.824578   -1.599474    1.368620
O      -3.751804   -0.630721   -0.816778
O       3.705375   -0.438647   -0.868360

Frequencies (cm-1)
  100.00    100.00    100.00    100.00    100.00    100.00    100.00    100.00    100.00    100.00
  100.00    106.62    127.05    133.69    152.20    162.40    166.14    177.53    190.13    191.35
  197.25    213.41    215.46    233.42    239.76    247.07    259.50    263.60    264.88    290.89
  297.58    307.68    319.99    333.14    336.07    346.31    353.39    360.05    364.79    411.95
  413.89    435.91    441.96    450.30    457.31    464.78    468.57    472.45    479.35    490.11
  558.30    562.77    583.38    595.80    600.51    612.91    660.79    681.86    704.53    713.39
  716.60    731.92    732.90    739.44    741.74    753.14    770.83    805.52    810.40    816.90
  826.19    830.73    845.18    855.11    878.54    891.02    897.12    901.54    902.69    913.55
  913.61    920.50    923.91    924.58    928.27    939.73    941.79   1012.14   1013.45   1020.92
 1022.25   1033.77   1042.77   1043.66   1052.60   1106.86   1115.10   1130.28   1137.38   1180.71
 1193.38   1199.54   1204.64   1212.73   1213.23   1219.54   1230.42   1251.01   1251.56   1274.84
 1310.66   1323.54   1326.04   1327.29   1329.69   1332.64   1333.61   1357.50   1357.96   1359.34
 1360.58   1407.11   1407.71   1421.93   1423.10   1424.52   1425.60   1426.90   1428.49   1432.69
 1433.03   1437.25   1439.01   1460.17   1460.39   1464.74   1475.11   1488.28   1519.93   1548.20
 1552.97   1555.72   1571.50   1594.11   1602.79   2986.18   2986.56   2988.80   2990.97   2994.52
```

```
 2997.54   3082.39   3082.69   3085.72   3085.96   3088.09   3093.58   3098.54   3102.35   3103.55
 3105.05   3116.13   3117.88   3119.70   3121.15   3134.04   3135.51   3166.99   3169.87   3170.99
 3191.39   3383.87   3457.53
Note: any frequencies below 100 cm-1 (including spurious imaginary ones) are upscaled to 100 cm-1
      for the calculation of thermodynamic properties.
      (Averkiev, Truhlar, Catal. Sci. Technol. 2011, 1, 1526)

Thermodynamics

Note: this script does not take into account the spin entropy
For more info, see eq. 3 of Inorg. Chem. 2002, 41, 6928-6935
(M. Reiher), https://doi.org/10.1021/ic025891l

Temperature is now: 298.150

Reporting max. of 0 frequencies (set by $GETFREQSMAX env. variable)


Reading 1 outputfiles

            ScnFrq(1)
---------------------
---------------------
(ZPVE)        299.167
(dH,0->T)      20.656
(-TS)         -57.005
(dGibbs)      262.818
=====================

Corresponding output files
 1 : frq_Ni-OH_-2_3.1327559.out
```

System nioh-Cs_0_1 (closed-shell singlet, C_S symmetry)

```
Reading 1 outputfiles

       Pauli         Elstat         OrbInt       Disp.       Solv.          TOTAL          Erel    Symm.       <S2>
-----------------------------------------------------------------------------------------------------------------------
-----
   33224.370     -7007.900     -34857.082     -34.263     -13.132      -8688.174         0.000     C(S)      0.000
ADF(1)

Corresponding output files
 1 : opt_nioh-Cs_0_1.1320454.out

Coordinates (Angs)
  60
opt_nioh-Cs_0_1.1320454.out        -8688.1699
Ni      0.540223    0.343618    0.000000
C       0.905179   -4.174756    2.132493
H       0.877055   -4.100410    1.040631
H       0.624846   -5.195024    2.409608
H       1.934833   -3.997431    2.462275
C      -1.482755   -3.443999    2.282505
H      -2.186218   -2.726027    2.712873
H      -1.798952   -4.452659    2.568863
H      -1.531753   -3.366293    1.190736
C       0.022817   -3.306045    4.297840
H       1.041382   -3.103880    4.646537
H      -0.247466   -4.324739    4.595482
H      -0.657771   -2.605405    4.785919
C      -0.055450   -3.176557    2.775030
C      -0.215975   -0.684206    2.665177
C       0.045822    1.680866    2.294009
C      -0.052933    2.100382    3.644155
H      -0.011214    1.359925    4.433688
C      -0.168251    3.435321    3.938729
H      -0.221583    3.751920    4.978503
C      -0.198636    4.407583    2.914975
H      -0.286310    5.460444    3.171490
C      -0.105547    4.039653    1.595447
H      -0.119004    4.819307    0.851129
C      -0.002019    2.671648    1.243113
C      -0.002019    2.671648   -1.243113
C      -0.105547    4.039653   -1.595447
H      -0.119004    4.819307   -0.851129
C      -0.198636    4.407583   -2.914975
H      -0.286310    5.460444   -3.171490
C      -0.168251    3.435321   -3.938729
H      -0.221583    3.751920   -4.978503
C      -0.052933    2.100382   -3.644155
H      -0.011214    1.359925   -4.433688
C       0.045822    1.680866   -2.294009
C      -0.215975   -0.684206   -2.665177
C      -0.055450   -3.176557   -2.775030
C       0.022817   -3.306045   -4.297840
H      -0.657771   -2.605405   -4.785919
H      -0.247466   -4.324739   -4.595482
H       1.041382   -3.103880   -4.646537
C      -1.482755   -3.443999   -2.282505
H      -1.531753   -3.366293   -1.190736
H      -1.798952   -4.452659   -2.568863
H      -2.186218   -2.726027   -2.712873
C       0.905179   -4.174756   -2.132493
H       1.934833   -3.997431   -2.462275
H       0.624846   -5.195024   -2.409608
H       0.877055   -4.100410   -1.040631
N       0.386424   -1.844038    2.338567
H       1.027214   -1.788407    1.534860
N       0.225925    0.410924    1.877760
N       0.025757    2.105797    0.000000
N       0.225925    0.410924   -1.877760
N       0.386424   -1.844038   -2.338567
H       1.027214   -1.788407   -1.534860
O      -1.092643   -0.555806    3.526800
O      -1.092643   -0.555806   -3.526800
O       1.645365   -1.170014    0.000000
H       2.554651   -0.835272    0.000000

Frequencies (cm-1)
  100.00    100.00    100.00    100.00    100.00    100.00    100.00    100.00    100.00    100.00
  104.13    108.86    112.16    128.77    148.15    181.05    186.88    194.91    197.30    200.86
  202.04    211.03    223.14    233.14    237.31    255.38    261.15    268.29    275.32    283.85
  327.24    327.83    336.12    337.41    341.83    354.33    357.73    367.66    379.85    406.78
  414.83    417.97    441.31    459.31    462.04    466.52    472.77    473.29    494.53    499.95
  525.19    556.19    578.65    581.28    601.87    668.11    687.10    710.71    715.00    731.04
  733.60    745.04    749.18    758.47    770.12    779.73    795.22    810.89    824.33    828.42
  846.26    850.60    867.20    882.15    899.97    906.00    907.90    914.19    916.81    917.45
  925.91    928.26    929.64    931.52    940.71    966.09    967.44    999.19   1013.42   1015.12
 1015.55   1021.09   1030.77   1037.53   1048.84   1131.83   1134.91   1141.23   1157.03   1198.79
 1205.19   1207.08   1210.07   1210.82   1216.81   1222.70   1224.41   1232.87   1258.22   1299.75
 1315.43   1331.70   1333.28   1337.67   1338.70   1352.51   1362.02   1363.38   1366.10   1376.09
 1396.38   1409.46   1410.85   1421.25   1421.97   1423.42   1423.96   1426.61   1431.07   1432.44
 1438.67   1441.71   1456.60   1459.96   1463.19   1468.10   1477.99   1539.78   1548.93   1554.79
 1567.09   1592.07   1604.10   1655.38   1661.34   2991.46   2992.98   2993.82   2995.62   2999.42
```

```
 3001.16   3089.35   3090.59   3091.89   3092.67   3094.40   3097.73   3105.17   3108.80   3109.73
 3110.20   3122.70   3122.73   3141.18   3141.28   3156.54   3156.93   3175.87   3202.80   3202.82
 3212.05   3228.65   3273.06   3745.31
Note: any frequencies below 100 cm-1 (including spurious imaginary ones) are upscaled to 100 cm-1
      for the calculation of thermodynamic properties.
      (Averkiev, Truhlar, Catal. Sci. Technol. 2011, 1, 1526)

Thermodynamics

Note: this script does not take into account the spin entropy
For more info, see eq. 3 of Inorg. Chem. 2002, 41, 6928-6935
(M. Reiher), https://doi.org/10.1021/ic025891l

Temperature is now: 298.150

Reporting max. of 0 frequencies (set by $GETFREQSMAX env. variable)


Reading 1 outputfiles

            ScnFrq(1)
--------------------
--------------------
(ZPVE)        300.904
(dH,0->T)      20.394
(-TS)         -56.489
(dGibbs)      264.810
====================

Corresponding output files
 1 : frq_nioh-Cs_0_1.1320484.out
```

System nioh-Cs-2_0_1 (open-shell singlet, C_S symmetry)

```
Reading 1 outputfiles

       Pauli        Elstat        OrbInt       Disp.       Solv.         TOTAL          Erel   Symm.      <S2>
-----------------------------------------------------------------------------------------------------------------
-----
   33184.508     -7009.542    -34811.638     -34.407     -12.372     -8683.619         0.000    C(S)     1.009
ADF(1)

Corresponding output files
 1 : opt_nioh-Cs-2_0_1.1320455.out

Coordinates (Angs)
Working on opt_nioh-Cs-2_0_1.1320455.out
  60
opt_nioh-Cs-2_0_1.1320455.out        -8683.6194
Ni      0.637050    0.351323    0.000000
C       0.899797   -4.189156    2.170849
H       0.906965   -4.133956    1.077145
H       0.595959   -5.200190    2.456844
H       1.920843   -4.020501    2.530495
C      -1.480171   -3.420711    2.230210
H      -2.189162   -2.691827    2.631751
H      -1.815856   -4.423422    2.514999
H      -1.494060   -3.352772    1.137038
C      -0.041209   -3.267976    4.291733
H       0.968853   -3.080097    4.671519
H      -0.343517   -4.274649    4.599103
H      -0.723603   -2.544448    4.743135
C      -0.065876   -3.166563    2.765482
C      -0.191272   -0.679576    2.598862
C       0.115856    1.696569    2.280325
C       0.052469    2.079854    3.628997
H       0.154673    1.328815    4.404313
C      -0.127114    3.411808    3.957384
H      -0.166723    3.707323    5.003418
C      -0.254814    4.380359    2.953096
H      -0.399829    5.424402    3.221094
C      -0.186576    4.031443    1.615271
H      -0.280456    4.813290    0.876924
C       0.011527    2.687126    1.249026
C       0.011527    2.687126   -1.249026
C      -0.186576    4.031443   -1.615271
H      -0.280456    4.813290   -0.876924
C      -0.254814    4.380359   -2.953096
H      -0.399829    5.424402   -3.221094
C      -0.127114    3.411808   -3.957384
H      -0.166723    3.707323   -5.003418
C       0.052469    2.079854   -3.628997
H       0.154673    1.328815   -4.404313
C       0.115856    1.696569   -2.280325
C      -0.191272   -0.679576   -2.598862
C      -0.065876   -3.166563   -2.765482
C      -0.041209   -3.267976   -4.291733
H      -0.723603   -2.544448   -4.743135
H      -0.343517   -4.274649   -4.599103
H       0.968853   -3.080097   -4.671519
C      -1.480171   -3.420711   -2.230210
H      -1.494060   -3.352772   -1.137038
H      -1.815856   -4.423422   -2.514999
H      -2.189162   -2.691827   -2.631751
C       0.899797   -4.189156   -2.170849
H       1.920843   -4.020501   -2.530495
H       0.595959   -5.200190   -2.456844
H       0.906965   -4.133956   -1.077145
N       0.413838   -1.847581    2.324103
H       1.065344   -1.815819    1.526066
N       0.308129    0.415255    1.824990
N       0.109548    2.134485    0.000000
N       0.308129    0.415255   -1.824990
N       0.413838   -1.847581   -2.324103
H       1.065344   -1.815819   -1.526066
O      -1.110497   -0.519938    3.406390
O      -1.110497   -0.519938   -3.406390
O       1.663193   -1.228260    0.000000
H       2.583201   -0.922963    0.000000

Frequencies (cm-1)
   100.00    100.00    100.00    100.00    100.00    100.00    100.00    100.00    100.00    100.00
   110.89    114.28    139.06    152.46    181.50    189.24    193.05    198.83    202.15    203.37
   206.59    211.23    228.69    236.25    237.10    259.08    260.71    264.00    280.58    300.22
   329.64    331.67    336.43    343.14    343.67    356.09    357.52    371.86    384.36    412.48
   417.56    425.45    453.50    459.92    464.68    468.16    470.49    479.49    505.08    510.19
   517.35    527.23    580.28    597.25    602.46    670.09    694.18    712.30    720.90    724.68
   732.53    733.73    750.97    767.84    771.36    781.87    809.27    810.92    822.36    836.75
   842.50    856.74    886.39    888.36    904.77    907.83    908.40    908.97    916.91    917.73
   918.66    927.58    929.70    940.76    944.52    955.07    957.21   1002.59   1008.16   1014.61
  1015.97   1030.61   1033.05   1035.10   1054.49   1125.85   1132.40   1139.49   1156.06   1200.72
  1202.97   1206.47   1210.69   1215.27   1223.54   1223.55   1231.31   1252.97   1260.59   1275.11
  1296.57   1308.22   1332.51   1334.30   1338.82   1340.15   1343.91   1361.81   1364.30   1366.88
  1382.52   1404.85   1409.98   1411.58   1422.46   1424.23   1424.92   1427.36   1431.94   1433.33
  1438.89   1440.80   1442.33   1451.64   1457.72   1460.95   1464.24   1549.04   1554.88   1555.81
```

```
 1566.80   1567.88   1580.07   1666.84   1668.42   2992.27   2993.70   2994.27   2995.96   2999.56
 3001.62   3090.33   3091.78   3092.98   3093.36   3094.90   3097.53   3104.85   3106.32   3111.47
 3113.22   3120.32   3120.37   3142.21   3142.40   3151.85   3156.56   3156.82   3186.95   3192.08
 3192.10   3216.51   3255.71   3738.58
Note: any frequencies below 100 cm-1 (including spurious imaginary ones) are upscaled to 100 cm-1
      for the calculation of thermodynamic properties.
      (Averkiev, Truhlar, Catal. Sci. Technol. 2011, 1, 1526)

Thermodynamics

Note: this script does not take into account the spin entropy
For more info, see eq. 3 of Inorg. Chem. 2002, 41, 6928-6935
(M. Reiher), https://doi.org/10.1021/ic025891l

Temperature is now: 298.150

Reporting max. of 0 frequencies (set by $GETFREQSMAX env. variable)


Reading 1 outputfiles

            ScnFrq(1)
---------------------
---------------------
(ZPVE)        300.914
(dH,0->T)      20.188
(-TS)         -55.864
(dGibbs)      265.238
=====================

Corresponding output files
 1 : frq_nioh-Cs-2_0_1.1327824.out
```

System nioh-Cs_0_3 (triplet, C_S symmetry)

```
Reading 1 outputfiles

       Pauli        Elstat        OrbInt       Disp.       Solv.         TOTAL          Erel    Symm.      <S2>
-------------------------------------------------------------------------------------------------------------------
-----
   33192.190     -7009.404    -34822.325     -34.382     -12.211     -8686.300         0.000    C(S)      2.008
ADF(1)

Corresponding output files
 1 : opt_nioh-Cs_0_3.1320428.out

Coordinates (Angs)
  60
opt_nioh-Cs_0_3.1320428.out        -8686.2998
Ni      0.639923    0.349772    0.000000
C       0.901478   -4.182427    2.166644
H       0.900821   -4.131454    1.072792
H       0.609693   -5.195279    2.458568
H       1.923165   -4.002177    2.518751
C      -1.485391   -3.436176    2.238123
H      -2.198154   -2.711301    2.640253
H      -1.810562   -4.440188    2.530334
H      -1.506444   -3.374078    1.144691
C      -0.034920   -3.262171    4.290178
H       0.975444   -3.061791    4.662653
H      -0.324326   -4.270724    4.603608
H      -0.722283   -2.544357    4.743252
C      -0.070471   -3.167396    2.763663
C      -0.214829   -0.680824    2.594967
C       0.110552    1.697452    2.279604
C       0.045343    2.078034    3.630289
H       0.136511    1.323504    4.403729
C      -0.126939    3.409096    3.960438
H      -0.171777    3.703325    5.006612
C      -0.242961    4.379924    2.956140
H      -0.381232    5.424603    3.225672
C      -0.170409    4.034246    1.617734
H      -0.252560    4.818675    0.880488
C       0.024101    2.690332    1.248989
C       0.024101    2.690332   -1.248989
C      -0.170409    4.034246   -1.617734
H      -0.252560    4.818675   -0.880488
C      -0.242961    4.379924   -2.956140
H      -0.381232    5.424603   -3.225672
C      -0.126939    3.409096   -3.960438
H      -0.171777    3.703325   -5.006612
C       0.045343    2.078034   -3.630289
H       0.136511    1.323504   -4.403729
C       0.110552    1.697452   -2.279604
C      -0.214829   -0.680824   -2.594967
C      -0.070471   -3.167396   -2.763663
C      -0.034920   -3.262171   -4.290178
H      -0.722283   -2.544357   -4.743252
H      -0.324326   -4.270724   -4.603608
H       0.975444   -3.061791   -4.662653
C      -1.485391   -3.436176   -2.238123
H      -1.506444   -3.374078   -1.144691
H      -1.810562   -4.440188   -2.530334
H      -2.198154   -2.711301   -2.640253
C       0.901478   -4.182427   -2.166644
H       1.923165   -4.002177   -2.518751
H       0.609693   -5.195279   -2.458568
H       0.900821   -4.131454   -1.072792
N       0.392865   -1.845341    2.313973
H       1.055645   -1.809397    1.526126
N       0.280290    0.416278    1.821629
N       0.141014    2.141805    0.000000
N       0.280290    0.416278   -1.821629
N       0.392865   -1.845341   -2.313973
H       1.055645   -1.809397   -1.526126
O      -1.133869   -0.524703    3.402742
O      -1.133869   -0.524703   -3.402742
O       1.681068   -1.220565    0.000000
H       2.600460   -0.913671    0.000000

Frequencies (cm-1)
   100.00     100.00     100.00     100.00     100.00     100.00     100.00     100.00     100.00     100.00
   111.63     113.76     134.31     150.88     160.96     184.84     186.27     193.38     196.90     198.33
   205.79     206.59     227.21     232.91     236.12     252.53     257.27     261.98     276.50     297.16
   329.77     331.08     334.55     341.52     341.97     355.26     356.73     372.08     379.35     412.16
   417.10     426.42     451.16     459.85     464.34     467.96     469.11     478.70     503.99     509.15
   520.71     527.06     582.72     597.10     602.75     669.03     697.62     711.59     719.64     724.63
   731.41     732.73     751.44     767.11     768.88     781.96     807.27     808.71     821.96     834.22
   843.30     852.73     881.00     883.60     904.74     907.30     908.46     910.49     917.56     918.04
   918.54     927.11     929.23     937.25     939.19     956.10     958.00     991.14    1007.19    1014.25
  1015.67    1030.09    1030.48    1034.56    1053.56    1125.50    1132.09    1144.97    1154.23    1199.55
  1204.13    1206.83    1210.89    1214.85    1219.18    1223.51    1231.47    1243.25    1253.37    1296.87
  1303.66    1312.14    1332.50    1334.28    1338.69    1339.96    1363.62    1364.28    1366.58    1366.90
  1381.30    1409.58    1411.15    1422.16    1423.93    1424.53    1427.16    1431.29    1432.88    1434.84
  1439.24    1442.29    1442.35    1451.86    1457.34    1460.55    1463.92    1550.80    1553.64    1555.73
  1566.54    1569.77    1582.73    1666.78    1669.75    2992.76    2994.03    2994.85    2996.16    2999.87
```

```
 3001.87   3090.87   3092.26   3093.41   3093.94   3095.65   3097.68   3105.47   3107.01   3111.55
 3113.27   3120.20   3120.25   3140.59   3140.80   3155.00   3155.30   3161.82   3190.14   3190.15
 3196.70   3215.43   3253.92   3740.27
Note: any frequencies below 100 cm-1 (including spurious imaginary ones) are upscaled to 100 cm-1
      for the calculation of thermodynamic properties.
      (Averkiev, Truhlar, Catal. Sci. Technol. 2011, 1, 1526)

Thermodynamics

Note: this script does not take into account the spin entropy
For more info, see eq. 3 of Inorg. Chem. 2002, 41, 6928-6935
(M. Reiher), https://doi.org/10.1021/ic025891l

Temperature is now: 298.150

Reporting max. of 0 frequencies (set by $GETFREQSMAX env. variable)


Reading 1 outputfiles

            ScnFrq(1)
---------------------
---------------------
(ZPVE)        300.896
(dH,0->T)      20.229
(-TS)         -55.896
(dGibbs)      265.230
=====================

Corresponding output files
 1 : frq_nioh-Cs_0_3.1320485.out
```
