# Supplementary material for: Enhanced Proton-Coupled Electron-Transfer Reactivity by a Mononuclear Nickel(II) Hydroxide Radical Complex
Source: Inorg Chem. 2024 Dec 16;63(52):24453–65. doi: 10.1021/acs.inorgchem.4c03370 (PMC11688665; doi:10.1021/acs.inorgchem.4c03370)
Supplement: Supplementary file 2 — ic4c03370_si_002.zip [file ic4c03370_si_002.zip › CompData/Reviewers-Link-Iochembd.pdf]

Reviewer’s link to the open data on iochem-bd

https://iochem.udg.edu/browse/review-collection/100/6283/6d2ba49c0ae908798d5f8c7b

The URL provided in the supporting information leads to the final URL, which will be opened up to the general public after the manuscript has been accepted.
Until that time the data is under embargo for the general public, but not for the reviewers. Reviewers have access to all data using the link above.
